# SNP-Density Crossover Maps of Polymorphic Transposable Elements and HLA Genes Within MHC Class I Haplotype Blocks and Junction

**DOI:** 10.3389/fgene.2020.594318

**Published:** 2021-01-18

**Authors:** Jerzy K. Kulski, Shingo Suzuki, Takashi Shiina

**Affiliations:** ^1^Faculty of Health and Medical Sciences, Medical School, The University of Western Australia, Crawley, WA, Australia; ^2^Division of Basic Medical Science and Molecular Medicine, Department of Molecular Life Science, Tokai University School of Medicine, Isehara, Japan

**Keywords:** MHC, haplotypes, snps, retroelements, crossovers, polymorphisms, indels

## Abstract

The genomic region (~4 Mb) of the human major histocompatibility complex (MHC) on chromosome 6p21 is a prime model for the study and understanding of conserved polymorphic sequences (CPSs) and structural diversity of ancestral haplotypes (AHs)/conserved extended haplotypes (CEHs). The aim of this study was to use a set of 95 MHC genomic sequences downloaded from a publicly available BioProject database at NCBI to identify and characterise polymorphic human leukocyte antigen (HLA) class I genes and pseudogenes, *MICA* and *MICB*, and retroelement indels as haplotypic lineage markers, and single-nucleotide polymorphism (SNP) crossover loci in DNA sequence alignments of different haplotypes across the *Olfactory Receptor* (*OR*) gene region (~1.2 Mb) and the MHC class I region (~1.8 Mb) from the *GPX5* to the *MICB* gene. Our comparative sequence analyses confirmed the identity of 12 haplotypic retroelement markers and revealed that they partitioned the *HLA-A/B/C* haplotypes into distinct evolutionary lineages. Crossovers between SNP-poor and SNP-rich regions defined the sequence range of haplotype blocks, and many of these crossover junctions occurred within particular transposable elements, lncRNA, *OR12D2, MUC21, MUC22, PSORS1A3, HLA-C, HLA-B*, and *MICA*. In a comparison of more than 250 paired sequence alignments, at least 38 SNP-density crossover sites were mapped across various regions from *GPX5* to *MICB*. In a homology comparison of 16 different haplotypes, seven CEH/AH (*7.1, 8.1, 18.2, 51.x, 57.1, 62.x*, and *62.1*) had no detectable SNP-density crossover junctions and were SNP poor across the entire ~2.8 Mb of sequence alignments. Of the analyses between different recombinant haplotypes, more than half of them had SNP crossovers within 10 kb of *LTR16B/ERV3-16A3_I, MLT1, Charlie*, and/or *THE1* sequences and were in close vicinity to structurally polymorphic *Alu* and *SVA* insertion sites. These studies demonstrate that (1) SNP-density crossovers are associated with putative ancestral recombination sites that are widely spread across the MHC class I genomic region from at least the telomeric *OR12D2* gene to the centromeric *MICB* gene and (2) the genomic sequences of MHC homozygous cell lines are useful for analysing haplotype blocks, ancestral haplotypic landscapes and markers, CPSs, and SNP-density crossover junctions.

## Introduction

The human major histocompatibility complex (MHC), also referred to as human leukocyte antigen (HLA), is investigated continuously because of its importance in the regulation of the innate and adaptive immune system, autoimmunity, and transplantation (Dawkins et al., 1999; Vandiedonck and Knight, 2009; Lokki and Paakkanen, 2019). The genomic region of the human MHC encompasses approximately 160 coding genes including three distinct structural regions: class I with the classical and non-classical HLA class I genes (*HLA-A, -B, -C, -F, -G*, and *-E*) and ~39 non-HLA genes, class II with the classical and non-classical HLA class II genes (*HLA-DRB1, -DRA, -DQA1, -DQB1, -DQA2, -DQB2, -DPA1*, and *-DPB1*) and class III that harbours more than 60 genes including the complement genes, *TNF, NFKBIL2*, and many other genes that code for cytokines, transcription factors, structural and developmental proteins (Shiina et al., 2004, 2009). The MHC class I and class II gene clusters contain numerous sequence duplications, insertions and deletions and considerable sequence diversity or polymorphisms (Trowsdale and Knight, 2013) that have accumulated into distinct multilocus haplotypes with relatively high population frequencies (>1%) (Awdeh et al., 1983; Degli-Esposti et al., 1992; Dawkins et al., 1999; Yunis et al., 2003; Goodin et al., 2018). These date from at least the beginning of human expansion and dispersal out of Africa, 50,000–100,000 years ago (Henn et al., 2012; López et al., 2015). The MHC multilocus haplotypes have been associated strongly with many diseases (Lokki and Paakkanen, 2019). On the basis of the large number of known *HLA-B* alleles, more than 20,000 different MHC multilocus haplotypes might be distributed worldwide in human populations, with less than a hundred in certain localised populations such as the Europeans (Steele and Lloyd, 2015; Jensen et al., 2017; Goodin et al., 2018). The common Northern European HLA haplotype *HLA-A1-B8-C7-DRB3-DQ2* (*8.1AH*) is estimated to have diverged from a single common ancestor about 23,500 years ago (Smith et al., 2006).

Although the MHC is highly polymorphic for single-nucleotide polymorphisms (SNPs), the degree of polymorphism (SNP density per 100 kb) depends on which haplotypes (haploid genotypes) are compared. There are at least two main types of genomic haplotype blocks that are studied for SNP variations: (1) those that are constructed on the basis of linkage disequilibrium (LD) statistical tests of a contiguous set of SNP markers (Ahmad et al., 2003; Walsh et al., 2003; Miretti et al., 2005; Blomhoff et al., 2006) and (2) those constructed from alignments of SNP density maps or genotyped alleles that identify well-defined haplotype blocks or segmental structures without using LD tests (Alper et al., 1983, 2006; Degli-Esposti et al., 1992; Dawkins et al., 1999; Aly et al., 2006; Smith et al., 2006; Lam et al., 2015; Alper and Larsen, 2017). If employed independently of each other, the two methods can result in unrelated single and/or multilocus haplotype block patterns (Yunis et al., 2003; Alper et al., 2006; Jensen et al., 2017). Homologous haplotype sequences have only a few detectable SNPs extended over a long-range of multilocus regions (Smith et al., 2006), whereas a large number of SNPs of varying density are detected in comparisons between different MHC class I haplotypes (Gaudieri et al., 1999, 2000; Miretti et al., 2005; Shiina et al., 2006, 2009; Jensen et al., 2017; Norman et al., 2017). The absence of SNPs over megabases of continuous sequence within the same MHC haplotypes is described as conserved sequence polymorphisms (CSPs) within conserved extended haplotypes (CEHs) (Yunis et al., 2003; Alper et al., 2006) and/or ancestral haplotypes (AHs) (Degli-Esposti et al., 1992; Dawkins et al., 1999), such as *8.1CEH/AH* (Price et al., 1999; Aly et al., 2006; Smith et al., 2006; Gambino et al., 2018), *7.1CEH/AH* (Gaudieri et al., 1997; Dunn et al., 2005), *57.1CEH/AH* (Dunn et al., 2005), *38.1CEH/AH* (Romero et al., 2007) and the Sardinian haplotype *18.2CEH/AH* (Contu et al., 1989; Bilbao et al., 2006). The non-LD, SNP-poor, long-range haplotypic sequences are mainly contained within polymorphic frozen blocks (PFBs) (Gaudieri et al., 1997; Dawkins et al., 1999) or fixed (conserved) haplospecific blocks (Alper et al., 2006; Barquera et al., 2020).

SNPs within many different recombinant haplotypes are absent for relatively much shorter distances ranging between 10 and 1,000 kb such as those found within PFBs of 60–300 kb (Gaudieri et al., 1997; Dawkins et al., 1999) and/or SNP-LD-blocks of ~18–50 kb (Daly et al., 2001; Jeffreys et al., 2004; Miretti et al., 2005; Blomhoff et al., 2006). The SNP-LD-block based on statistical associations between the frequencies of two or more genotyped loci in population studies cannot map the classical CEH/AH or PFB architectural structures directly or reliably (Schaid et al., 2002; Alper et al., 2006; Slatkin, 2008), whereas reliable linkage mapping is usually dependent on pedigree studies of particular genotyped markers to evaluate their linkage or segregation in meiosis or on phased genomic sequences (Alper and Larsen, 2017) such as those that have been sequenced or genotyped using multilocus HLA-captured haplotype phasing (Guo et al., 2006), *de novo* assembled trios (Jensen et al., 2017), MHC homozygous cell lines (Dorak et al., 2006; Horton et al., 2008; Norman et al., 2017), sperm (Cullen et al., 2002; Kirkness et al., 2013) or single chromosomes (Murphy et al., 2016). SNP-LD analyses often fail to detect linkage of multiple loci within the conserved haplotype structure as effectively as the genes that may be involved in disease susceptibility or resistance because of the use of non-haplotypic SNP markers (Alper et al., 2006; Slatkin, 2008; Alper and Larsen, 2017). Nevertheless, the SNP-LD-blocks that were identified by LD or long-range haplotype (LRH) and extended haplotype homozygosity (EHH) tests (Traherne, 2008) of the MHC genomic regions include a variety of genotyped haplotypic microsatellites (Karell et al., 2000; Doxiadis et al., 2007), SNPs (Ahmad et al., 2003; de Bakker et al., 2006; Shiina et al., 2006; Smith et al., 2006; Romero et al., 2007; Lam et al., 2013), and indels (WGS500 Consortium et al., 2014; Jensen et al., 2017; Huang et al., 2019) as well as structural dimorphic retroelements (REs), such as *Alu, SVA, LTR*, and *HERVs* (Kulski and Dunn, 2005; Kulski et al., 2011).

Segmental shuffling is a meiotic recombination or crossing over process between different haplotypes (Gaudieri et al., 1997; Traherne et al., 2006) that often occurs within nucleotide sequences in regions between the alpha, beta, epsilon and delta frozen polymorphic blocks (Dawkins et al., 1999; Traherne et al., 2006; Romero et al., 2007), although breakpoints have been reported also within and between the HLA class I genes within the alpha (Lam et al., 2013) and beta blocks (Nair et al., 2006) and between the HLA class II genes within the delta block (Jeffreys et al., 2004; Larsen et al., 2014). Many MHC recombinant haplotypes appear to have originated in relatively recent times (Smith et al., 2006; Lam et al., 2013) due to the pressures of bottlenecks, migrations and gene flow, inbreeding, and outbreeding in various times of abundance and deprivation (van Oosterhout, 2009; Lobkovsky et al., 2019; Wang et al., 2020). With the formation of new human MHC haplotypes during and/or after speciation, many of the high-frequency (>1%) AHs were preserved over numerous generations and migrations; even across different ethnic populations as deduced from the European (Contu et al., 1989; Aly et al., 2006; Bilbao et al., 2006; Smith et al., 2006) and Asian haplotypes (Lam et al., 2013, 2015, 2017).

Much of genomic sequence diversity, including haplotype diversity, is driven by molecular mechanisms such as DNA repair, replication, single point mutations, indels, recombination, duplication, conversion, transposition, and segmental rearrangements (Gu et al., 2008; Brawand et al., 2014; Lin and Gokcumen, 2019). In addition, interspersed repeat sequences that contribute to >50% of the human genomic content (de Koning et al., 2011) have been implicated in a variety of these DNA molecular processes (Moolhuijzen et al., 2010; George and Alani, 2012; Raviram et al., 2018; Lu et al., 2020). The identification of transposable elements (TEs) near the junctions of duplicated genes (Kulski et al., 1997, 1999b, 2004) and at ectopic and meiotic recombination sites (Myers et al., 2010; Altemose et al., 2017; Kent et al., 2017) emphasise their role in driving genomic diversity. Interspersed REs, because of their mobility, hypermutability, and potential role in meiotic recombination, are an integral part of molecular drive (Dover, 1982) that together with point mutations, gene conversion (Madrigal et al., 1993; Adamek et al., 2015) and balancing selection (van Oosterhout, 2009) with a component of multiplicative fitness (Lobkovsky et al., 2019) probably have generated and maintained haplotypic polymorphisms in the MHC class I regions. This multifunctional role for active REs is evidenced in part by the structural biallelic *Alu, SVA, LTR*, and *HERVs* located near to or within putative recombination hotspots throughout the MHC class I, II, and III genomic regions (Kulski et al., 2011). In recent years, the proposed broad roles for TE and polymorphisms in the regulation of meiotic recombination (a mechanism that undoubtedly generated the MHC haplotype diversity in humans) has gained increasing attention (Myers et al., 2008; Zamudio et al., 2015; Altemose et al., 2017; Kent et al., 2017; Bourgeois and Boissinot, 2019).

Both first-generation and second-generation sequencing methods have produced phased genomic sequences of representative MHC haplotypes by using MHC homozygous cell lines (Horton et al., 2004, 2008; Stewart et al., 2004; Traherne et al., 2006; Norman et al., 2017). These phased MHC genomic sequences are important reference DNA sequences that provide representative haplotypes for better informed large population studies and for mapping heterozygous sequence reads such as by inference graphs (Dilthey et al., 2016), SNP-LD based haplotype frequencies (Romero et al., 2007) and EEH tests (Lam et al., 2015), especially for disease associations (Alper and Larsen, 2017; Lokki and Paakkanen, 2019). Although Norman et al. (2017) produced an important database for 95 MHC homozygous cell lines of assembled and resolved MHC genomic sequences, they limited their own analysis to the multilocus alleles and haplotypes of the HLA classical class I and class II genes, *MUC22* and the structural diversity of *C4* duplications. Missing from their analysis are the many REs, repeats and retrotransposable subfamilies, as well as the amplified and duplicated members of the genomic DNA that make up >50% of the human DNA content and that contribute to disease (Ayarpadikannan and Kim, 2014; Payer et al., 2017; Payer and Burns, 2019), gene regulation and recombination (Moolhuijzen et al., 2010; Myers et al., 2010; Altemose et al., 2017; Chuong et al., 2017) and to the duplicated segmental organisation of the human and other primate MHC genomic structures (Kulski et al., 1997, 1999a,b; Anzai et al., 2003; Kulski et al., 2004).

The purpose of the present study was to extend the Norman et al. (2017) analysis by investigating the haplotypic linkages between the MHC class I genic and intergenic regions including *HLA-F, HLA-G, MICA, MICB*, eight HLA pseudogenes (*HLA*-*V, -P, -H, -T, -K, -U, -W*, and *-J*) and a set of previously published biallelic REs, *AluOR* (Kulski et al., 2014), *AluHF, AluHG, AluHJ, AluTF, AluMICB* (Kulski and Dunn, 2005; Kulski et al., 2019), *HERVK9* (Kulski et al., 2008), *MER9* (Kulski et al., 2009) and four biallelic SVA haplotypic markers, *SVA-HA, SVA-HC, SVA-HB*, and *SVA-HF* (Kulski et al., 2010, 2011). A further aim was to identify and characterise the ancestral SNP-density crossover (XO) loci in DNA sequence alignments of different haplotype blocks or segments from the *GPX5* gene in the *OR* gene region telomeric of *HLA-F* to the centromeric *MICB* gene within the MHC class I genomic region. The overall results of the study suggest that the SNP XOs are indicators of haplotype XO, which in turn point to putative ancestral recombination sites that are widely distributed across the 2-Mb-MHC class I genomic region from telomeric of *HLA-F* to centromeric of *MICB*.

## Materials and Methods

The haplotype data of 95 MHC genomic sequences sequenced and assembled from HLA-homozygous cell lines by Norman et al. (2017) at NCBI BioProject with the accession number PRJEB6763 (https://www.ncbi.nlm.nih.gov/bioproject/) were downloaded as Fasta files and used for the analyses described below. The other MHC genomic sequences used in haplotype analyses were the GRChr38.p13 (GCF_000001405.39) of the chromosome 6 reference NC_000006.12 at the NCBI (https://www.ncbi.nlm.nih.gov/assembly/GCF_000001405.39/), UCSC (https://genome.ucsc.edu/cgi-bin/hgGateway) and eEnsembl (http://asia.ensembl.org/Homo_sapiens/Info/Index) browsers and databases, the eight human reference haplotypes described by Horton et al. (2008), the chimpanzee sequence of Anzai et al. (2003) and the gorilla sequence of Wilming et al. (2013). All of the Fasta sequences downloaded from the public archives were submitted to the RepeatMasker webserver (http://www.repeatmasker.org/cgi-bin/WEBRepeatMasker) for output files of annotated members of the interspersed repetitive DNA families, their locations in the sequence and their relative similarity or identity in comparison to reference sequences of SINEs, LINEs, LTRs, ERVs, DNA elements, small RNA, and simple repeats. For the online analysis, RepeatMasker used the Dfam database (3.0) for the repeat sequence comparisons (Hubley et al., 2016) (http://www.dfam.org) because since 20th May 2019, it no longer had access to the RepBase library of repetitive elements (Bao et al., 2015) previously provided by GIRI (https://www.girinst.org/repbase/). The main difference between the Dfam database and RepBase for our analysis was that Dfam listed many Alu-like short sequences as *SVA*, whereas we were interested only in the *SVA* mosaic of 500 to 1,800 bp in RepBase with structures similar to those described by Shen et al. (1994). Thus, we used four dimorphic *SVA* sequences (*SVA-HA, SVA-HC, SVA-HB*, and *SVA-MIC*) previously reported by Kulski et al. (2010) and added three new dimorphic *SVA* sequence markers to this analysis (Table 1).

**Table 1:** Dimorphic retroelements (absent or present) and STR analysed in this study.

**Retroelement or microsat**	**Nearest flanking (/) genes**	**Location within genome reference Ch38/hg38, Chr 6**	**Popln frequencies caucasian/Japanese (*****n*** **= 88–260)**		**References**
AluOR	OR12D2 intron	29396132–29396263	0.14	0.32	Kulski et al., 2014
AluOR1	3′OR12D1	29416044			Present study
AluHF	ZFP57/HLA-F	~29710985^*^	0.23	0.06	Dunn et al., 2002
AluHG	HLA-G/HLA-H	~29850749^*^	0.30		Kulski et al., 2001
			0.30	0.21	Dunn et al., 2002
AluHJ	HLA-J/ETF1P1	~30030620^*^	0.25	0.38	Dunn et al., 2002
AluTF	MUC21/MUC22	~31003947^*^	0.11	0.08	Dunn et al., 2003
AluP5	MICA/MICB	~31470733^*^			Present study
AluMICB	MICB intron 1	~31498446^*^	0.12		Kulski et al., 2002a
			0.16	0.12	Kulski and Dunn, 2005
HERVK9	HLA-G/HLA-H	29875649–29881829	0.37	0.59	Kulski et al., 2008
sMER9 (1)	HLA-G/HLA-H	~29881317^*^	0.66	0.41	Kulski et al., 2008
LTR13	HLA-K/HLA-U	29929971–29930908			Present study
sMER9 (2)	HLA-U/HLA-A	29936175–29936676			Kulski et al., 2009
LTR5L	TRIM26/HLA-L	~30221451			Present study
MER5/LTR33	HLA-C/HLA-B	~31313186			Present study
HAL1/MER5A	MICA/MICB	31418238–31418519			Present study
LTR9	MICA/MICB	31423445–31424086			Present study
SVAOR	3′GPX6	28501515–28503131			Present study
SVA-HF	LTR16/HLA-F	29717873–2972077	0.14	0.00	Kulski et al., 2010
SVA-16^**^	HLA-H/HLA-T	29895386–29896449	fixed		Present study
SVA-HA	HLA-K/HLA-A	29932087–29933753	0.26	0.06	Kulski et al., 2010
SVA-T26^**^	TRIM26/HLA-L	30221503–30222724			Present study
SVA-ER^**^	MICC/HLA-E	30474489–30475999			Present study
SVA-EG^**^	HLA-E/GNL1	30498159–30499333			Present study
SVA-M21^**^	MUC21/MUC22	30992538–30993994			Present study
SVA-M22^**^	MUC22/C6orf15	31066602–31068056			Present study
SVA-HC	HCG27/HLA-C	31243860–31245322	0.10	0.03	Kulski et al., 2010
SVA-CB	HLA-C/HLA-B	~31310982^*^			Present study
SVA-HB	HLA-C/HLA-B	~31329940^*^	0.65	0.25	Kulski et al., 2010
SVA-MIC	MICA/MICB	31453745–31456553			Kulski et al., 2010
9.5-kb del	HLA-C/HLA-B	~31298645^*^			Present study
(ATAG)n	HLA-G/MICF	29838629–29838750			Present study
(CAGAGA)n	HLA-G/MICF	29838997–29839045			Present study
(ATAA)n	HLA-A/HLA-W	29949553–29949592			Present study
(ATTT)n	HLA-A/HLA-W	29949590–29949639			Present study
(TTTA)n	TRIM26/HLA-L	30221462–30221500			Present study
(GAGG)n	MUC22/C60rf15	~ 31066254			Present study
(TTTC)n	HCG27/HLA-C	31236405–31236481			Kulski et al., 1997
(ACA)n	HCG27/HLA-C	31239846–31239880			Kulski et al., 1997
(TTCC)n	HCG27/HLA-C	31241314–31241352			Kulski et al., 1997
(TTAT)n	HLA-C/HLA-B	31321185–31321227			Kulski et al., 1997
(CTG)n	within MICA	31412369–31412393			Mizuki et al., 1997
(TGT)n	within MICA	~31412394^*^			Present study

* Approximate location because these deletions, retroelements or STR are absent from the Ch38/hg38 Genome Reference that has the HLA haplotype of HLA-A*03:01:01:01/ B*07:02:01:01/C*07:02:01:03/.

** *SVA were present in all 95 haplotypes of the Norman et al. (2017) sequences and chimpanzee (Anzai et al., 2003), and therefore are fixed in humans*.

Norman et al. (2017) provided the alleles of the *HLA-A, -B*, and *-C* class I genes for all the 95 cell line sequences shown in Supplementary Table 1. We confirmed the alleles of the HLA class I genes and included the alleles of *HLA-E, -F*, and -*G*, and the *MICA* and *MICB* genes and eight HLA-A class I pseudogenes (Supplementary Table 2) in the 95 cell line sequences by comparing them to the IMGT HLA allele sequences (IMGTRelease 3.38.0) using the DNA sequence assembly software Sequencher ver.5.0 (Gencode http://www.genecodes.com). The alleles that were not in the IMGT HLA allele databases (Robinson et al., 2019) at https://www.ebi.ac.uk/ipd/imgt/hla/ are reported here as “new” without providing any further information about the novel nucleotide or amino acid differences. We also found that the FTQW01000001.1 sequence provided by Norman et al. (2017) as the chimpanzee “Clint” (*Pan troglodytes* genome assembly, contig: 1_COX_Oct2016_Scaffold, whole genome shotgun sequence) has strong identity with the COX cell line sequence that harbours the *8.1AH* haplotype A^*^01:01:01:01/ B^*^08:01:01:01/ C^*^07:01:01:01 (Horton et al., 2008).

We added a laboratory identifier number (ID_1 to ID_95) to each of the Norman et al. (2017) sequences (Supplementary Table 2) for ease of identification in comparative sequence analysis. A shorthand identifier for the MHC CEH/AH haplotypes based on the *HLA-B* allele such as *7.1CEH/AH, 8.1CEH/AH, 13.1CEH/AH* was used as previously described (Degli-Esposti et al., 1992, Dawkins et al., 1999, Dorak et al., 2006). The alleles of the HLA class I genes, MIC genes, HLA class I pseudogenes and HLA class II genes were determined also for the GRChr38p13 genomic reference sequence, which corresponds to the *7.1AH* of the PGF homozygous cell line (Horton et al., 2008), shown in Supplementary Table 3. The dimorphic RE and microsatellite markers that were searched for and identified by RepeatMasker in the 95 MHC genomic sequences are shown in Table 1. The RE dimorphisms (absence or presence) were easily recognised in each of the RepeatMasker outputs because of their positions within or close proximity to other TE elements and short tandem repeats (STRs). For example, the *MER9/HERVK9-int/MER9* insertion at nucleotide positions (nts) 160655 to 166834 in Supplementary Table 4 is flanked by a string of telomeric *LTR16B2/MLT1F1/ STR/AluY/STR/L1ME3* elements and a string of centromeric *Charlie9* (nts, 89–303)*/Charlie9* (nts, 1283–1803)*/L1PA10/LMLT1F1/THE1C* elements that are easily identified in the RepeatMasker outputs with the solitary *MER5* and *HERVK9-int* deletion at their corresponding locations.

Comparative sequence alignments between two or more sequences to evaluate SNP densities and determine XO regions between SNP-poor regions (SPR) of <20 SNPs per 100 kb and SNP-rich regions (SRR) of >100 SNPs per 100 kb were performed with the web-based MultiPipMaker alignment program (http://pipmaker.bx.psu.edu/cgi-bin/multipipmaker) by uploading the Fasta sequence files, a RepeatMasker output file and using the MultiPipMaker setting for single coverage as described by Schwartz et al. (2000) to generate the optimal sequence alignment. SNPs in the alignments were counted twice manually, and an average number was presented in the results. Obvious assembly errors, polynucleotides, simple microsatellite repeats and indels were not counted as SNPs. Also, a series of many adjoining SNPs (e.g., >5 SNPs in a string of 50 nucleotides) or SNPs within 50 bp of obvious sequencing errors with runs of unspecified nucleotides (Ns) and/or inconsistent long strings of deletions were not counted. The length of sequence alignments usually ranged between 50 and 500 kb depending on (1) the segments targeted for the analysis and the ease of SNP manual counting in the pdf outputs of the nucleotide alignments and/or (2) the length of the Percentage Identity Plot (PIP) output for reproduction as a convenient and readable image. The targeted sequences were selected and trimmed from the Fasta files that had been previous downloaded from the NCBI BioProject, accession number PRJEB6763. The software program Genetyx ver.20 (GENETYX Co., Tokyo, Japan) was used with the Selector function set to select and trim to obtain the required Fasta file sequences with the genomic sequence target positions taken from those listed in the RepeatMasker output text file (Supplementary Table 3). The T-Coffee multiple sequence alignment tool at EMBL-EBI (https://www.ebi.ac.uk/Tools/msa/tcoffee/) was used to construct multiple sequences of *ERV3-16A3-int* in the Fasta format and the CLUSTALW (1.83) format.

## Results

### MHC Haplotype Sequences

Of the 95 human MHC haplotypes sequenced by Norman et al. (2017), 82 differed at least at one of the 9 loci, *HLA-A, -C, -B, -DRB1, -DRB345, -DQA1, -DQB1, -DPA1*, and *-DPB1*. However, there were 46 sequences representing 18 haplotypes that had the same combination of *HLA-A, -C*, and *-B* alleles for at least one haplotype pair. Furthermore, 70 sequences represented 19 different *HLA-C/-B* haplotypes and 67 sequences represented 23 different *HLA-A/-C* haplotypes (Supplementary Table 1) with a homologous alignment for at least one haplotype pair.

In this study, the haplotypic alleles of 56 loci were analysed, ranging between the *OR* gene region and the MHC class I region including the classical *HLA-A, -B*, and *-C* loci, the non-classical *HLA-F, -G*, and *-E* loci, 8 HLA pseudogenes, *MICA* and *MICB*, 8 *Alu* loci, 13 *SVA* loci, 2 *MER9* loci, the *HERVK9* locus, 5 LTR or *MER5* loci, and 12 STR loci (Tables 1–3, Supplementary Table 2). The 9.5-kb *MER5/LTR33* indel between the *HLA-C* and *HLA-B* loci also contained within its sequence a string of different *L1* fragments, *ERVL-E-int* fragments, *MER3, MIR, AluJ, MLT1B, LTR84b, MLT1G3, AluSx*, and *MLT2C1* beside the *MER5* and *LTR33* elements (Supplementary Figure 1). There were numerous other indels ranging between 1 and 40 kb within the beta block sequences (Supplementary Figures 2–4) that were not included as allelic markers in this study. To assess the MHC class I haplotypic integrity of the 95 cell lines, the additional allelic haplotype combinations that we typed were sorted and grouped according to alpha block haplotypes (Table 2, Supplementary Table 5) and beta block haplotypes (Table 3, Supplementary Table 6) and then used for SNP XO studies across ~3 Mb of sequence between *GPX5* and *MICB* (Tables 4–**8**).

**Table 2:** Alpha block haplotypes and alleles from *AluHF* to *AluHJ* including *HLA-F, -G, -H, -A*, and *-J* alleles.

**Hap ID**	**No. Hap**	***AluHF***	***HLA-F***	***HLA-G***	***AluHG***	***ERVK9***	***HLA-H***	***HLA-A***	***HLA-J***	***AluHJ***
1	5	1	01:01:01:09	01:01:02	1	1	02:01	01:01:01	01:01:01:02	2
2	9	1	01:01:01:09	01:06	1	1	02:01	01:01:01	01:01:01:02	2
3	13	1	01:01:01:01	01:01:01	2	1	01:01	02:01:01	01:01:01:05	1
4	1	1	01:01:01:01	01:01:01	2	1	01:01	02:01:01	01:01:01:04	1
5	1	1	01:01:01:01	01:01:01	2	1	01:01	02:01:01	01:01:01:05	1
6	1	1	01:01:01:04	01:01:01	2	1	01:01	02:01:01	01:01:01:05	1
7	3	1/2	01:01:01:08	01:01:01	2	1	01:01	02:01:01	01:01:01:05	1
8	2	1/2	01:01:01:08	01:01:01	2	1	01:01	02:01:01	01:01:01:02	1
9	1	1	01:01:01:09	01:01:01	2	1	01:01	02:01:01	01:01:01:05	1
10	1	1	01:04:01:02	01:01:01	2	1	01:01	02:01:01	01:01:01:05	1
11	1	1	01:01:02:07	01:03:01	1	1	New	02:05:01	New	1
12	1	1	01:01:01:09	01:01:01	2	1	01:01	02:01:01	01:01:01:05	1
13	1	1	01:04:01:02	01:01:01	2	1	01:01	02:01:01	01:01:01:05	1
14	1	1	01:01:02:07	01:03:01	1	1	New	02:05:01	New	1
15	1	1	01:01:01:18/19	01:01:22	1	1	02:04	03:01:01	01:01:01:02	2
16	6	1	01:03:01:01/04	01:01:01	1	2	02:04	03:01:01	01:01:01:04	1
17	2	1	01:01:02:09/12	01:01:03	1	2	New	11:01:01	01:01:01:04	1
18	1	1	01:03:01:03	01:04:04	1	2	Deletion	23:01:01	01:01:01:04	1
19	1	1	01:01:01:08	01:04:01	1	2	Deletion	24:02:01	01:01:01:02	2
20	3	1	01:01:01:09	01:04:01	1	2	Deletion	24:02:01	01:01:01:02	2
21	1	1	01:01:01:09	01:04:01	1	2	Deletion	24:02:01	01:01:01:04	1
22	1	1	01:01:02:10	01:04:01	1	2	Deletion	24:02:01	01:01:01:02	2
23	3	1	01:01:01:18/19	01:01:02	1	2	Deletion	24:02:01	01:01:01:02	2
24	3	1/2	01:01:01:08	01:01:02	1	1	01:02	26:01:01	01:01:01:08	1
25	4	2	01:01:01:08	01:01:01	1	1	02:02	29:02:01	01:01:01:01	1
26	1	1	01:01:01:09	01:01:02	1	1	New	30:01/A68	New	1
30	1	1	01:01:01:09	01:05N	1	1	New	30:01:01	New	1
31	2	1	01:01:01:01/17	01:01:01	2	1	New	30:02:01	01:01:01:04	1
32	1	1	01:01:01	01:03:01	2	1	01:01	31:01:02	01:01:01:05	1
33	3	1	01:01:01:11	01:03:01	1	2	New	31:01:02	New	1
34	2	1	01:01:02:06/10	01:01:22	1	1	02:03	32:01:01	01:01:01:06	2
35	1	1	01:01:02:06	01:01:12	1	1	02:03	32:01:01	01:01:01:06	2
36	2	1	01:01:02:07	01:03:01	1	2	New	33:01:01	New	1
37	1	1	01:01:02:10	01:04:01	1	2	New	33:01:01	New	1
38	1	1	01:01:01:08	01:01:02	1	1	01:02	66:01:01	New	1
39	1	2	01:01:01:18/19	01:01:02	1	1	New	68:02:01	New	1

*For AluHF, AluHG, AluHJ, and ERVK9, allele 1 is the absence of the element and allele 2 is the presence of the element. This nine-marker table is a summary of the more detailed Supplementary Table 4 with 21 markers*.

**Table 3:** Beta block haplotypes.

**Hap ID**	**No. Haps**	***SVA-HC***	***HLA-C***	***SVA-BC***	**9.5 kb indel**	***SVA-HB***	***HLA-B***	***SVA-MIC***	***MICA***	***MICB***	**CEH/AH**
1	2		01:02:01	2	2	1	46:01:01	2	010:01	005:02	46
2	1	1	01:02:01	2	2	1	51:01:01	2	010:01	005:02	51
3	1	1	01:02:01	2	2	1	54:01:01	1	012:01	005:02	54
4	1	1	01:02:01	2	2	1	56:01:01	1	012:01	005:02	56
5	1	1	01:02:01	2	2	1	15:01:01:01	2	010:01	006	15
6	1	1	03:04:01:01	1	2	1	40:01:02	2	008:04	002:01	40
7	5	1	05:01:01:01	1	2	1	18:01:01:01	1/2	001	005:02	18.2
8	5	1	05:01:01:02	1	2	1	44:02:01:01	1	008:01	005:02	44.1
9	2	1	07:01:01:01	1/2	2	1	18:01:01:02	1/2	018:01	002:01	18.
10	1	1	07:01:01:01	2	2	1	49:01:01	1	004	005:02	49.x
11	1	1	07:01:01:01	2	2	1	57:01:01	2	017	003	57.1
12	8	2	07:02:01:03	1	2	1	07:02:01	2	008:04	004:01	7.1
13	1	1	08:02:01:01	1	2	1	14:01:01	2	019:01	005:02	14.x
14	2	1	08:02:01:01	1	2	1	14:02:01	1	011	005:02	14.y
15	1	1	12:02:02	1	2	0	52:01:01	2	009:01	002:01	52.1
16	1	1	14:02:01	2	2	1	51:01:01	2	049	005:02	51
17	1	1	14:03	2	2	1	44:03:01	2	004	005:02	44
18	1	1	15:02:01	1	2	1	51:01:01	2	009:01	002:01	51.y
19	2	1	15:02:01	1	2	1	51:01:01	2	009:01	005:02	51.x
20	2	1	01:02:01	1	2	2*	27:05:02	1	007:01	005:02	27.1
21	1	1	02:02:02:01	1	2	2	27:05:02	1	007:01	005:02	27.x
22	2	1	02:02:02:01	1	2	2	40:02:01	2	027	005:02	40.x
23	1	1	02:02:02:01	1	2	2	40:02:01	2	027	013	40.y
24	2	1	03:03:01	1	2	2	15:01:01:01	1/2	010:01	002:01	15.x
25	1	1	03:03:01	1	2	2	15:01:01:01	2	010:01	005:02	15.y
26	3	1	03:04:01:01	1	2	2*	15:01:01:01	2	010:01	002:01	62.1
27	1	1	03:04:01:01	1	2	2	40:01:02	2	008:04	002:01	60.x
28	1	1	03:04:01:01	1	2	2	40:01:02	2	008:04	004:01	60.y
29	1	1	03:04:01:01	1	2	2	40:01:02	2	008:04	014	60.z
30	1	1	C*04:01:01:01	1	2	2*	15:26N	2	010:01	005:02	15.n
31	1	1	C*04:01:01:01	1	2	2	35:01:01:01	0	002:01	005:02	35.x
32	1	1	C*04:01:01:01	1	0	2	35:01:01:01	2	017	003	35.y
33	1	1	C*04:01:01:01	0	2	2*	35:01:01:02	1	002:01	002:01	35.2
34	2	1	C*04:01:01:01	1	2	2	35:02:01	2	016	005:01	35.z
35	1	1	C*04:01:01:01	1	2	2	35:03:01	1	002:01	005:02	35.w
36	1	1	C*04:01:01:01	1	2	2*	35:08:01	2	016	002:01	35.v
37	1	1	C*04:01:01:01	1	2	2*	53:01:01	1	002:01	006	53.x
38	3	1	06:02:01:01	1	0	2	13:02:01	2	008:01	005:02	13.1
39	1	1	06:02:01:01	1	0	2*	37:01:01	2	010:01	002:01	37.x
40	1	1	06:02:01:01	1	0	2	40:01:02	2	008:04	004:01	40.x
41	1	1	06:02:01:01	1	0	2	47:01:01:	2	008:01	004:01	47.1
42	4	1	06:02:01:01	1	0	2	57:01:01	2	017	003	57.1
43	1	1	06:02:01:02	1	0	2	50:01:01	1	009:02	005:06	50.1
44	5	1	07:01:01:01	1	2	2/2*	08:01:01	1	008:01	008	8.1
45	1	1	07:01:01:01	1	2	2	08:01:01	2	008:04	004:01	8.x
46	1	1	07:18	1	2	2*	58:01:01	1	002:01	008	58.1
47	3	1	12:02:02	1	2	2/2*	52:01:01	2	009:01	005:03	52.1
48	1	1	12:03:01:01	1	0	2	35:03:01	1	002:01	005:02	35.2
49	3	1	12:03:01:01	1	0	2/2*	38:01:01	1	002:01	002:01	38.x
50	1	1	12:03:01:01	1	0	2*	51:01:01	2	006	005:02	51.x
51	3	1	16:01:01	1	2	2	44:03:01	1	004	005:02	44.2
52	1	1	16:01:01	1	2	2	45:01:01	2	015	002:01	45.x
53	1	1	17:01:01:02	1	2	2	41:01:01	1	004	005:02	41.x
54	1	1	17:01:01:02	1	2	2	42:01:01	1	004	002:01	42.1

SVA-HB allele 2^*^ is a SVA-HB duplicated sequence within LTR10/HERVI/LTR10 rearrangements that do not correlate with any particular HLA-C lineage and therefore may be sequence assembly errors. For SVA, Alu, and the indel, allele 1 is the absence of the element and allele 2 is the presence of the element.

**Table 4:** SNP-poor (SP) and SNP-rich (SR) haplotypes in the MHC class I region from *GPX5* to *MICB*.

**Lab ID**	**CEH**	**Haplotype**	**SP region**	**SNPs/100 kb**	**Sequence length kb**
4	7.1	A*03:01/C*07:02/B*07:02			2,939
6	7.1	A*03:01/C*07:02/B*07:02	SP across whole region	0.816	1,962
51	7.1	A*03:01/C*07:02/B*07:02	SP across whole region	0.749	2,938
75	7.1	A*03:01/C*07:02/B*07:02	SP across whole region	1.124	2,937
90	7.1	A*03:01/C*07:02/B*07:02	SP across whole region	1.054	2,942
27	8.1	A*01:01/C*07:01/B*08:01			2,996
11	8.1	A*01:01/C*07:01/ B*08:01	SP across whole region	0.716	2,933
12	8.1	A*01:01/C*07:01/B*08:01	SP across whole region	0.86	3,023
16	8.1	A*01:01/C*07:01/B*08:01	SP across whole region	1.662	2,948
19	8.1	A*01:01/C*07:01/B*08:01	SP across whole region	1.181	2,963
25	18.2	A*30:02/C*05:01/B*18:01			2,988
26	18.2	A*30:02/C*05:01/B*18:01	SP across whole region	0.68	2,940
67	51.x	A*02:04/C*15:02/B*51:01			2,952
76	51.x	A*02:04/C*15:02/B*51:01	SP across whole region	0.443	2,937
37	57.1	A*02:01/C*06:02/B*57:01			2,984
58	57.1	A*02:01/C*06:02/B*57:01	SP across whole region	0.546	2,932
17	62.x	A*02:01/C*03:03/B*15:01			2,944
32	62.x	A*02:01/C*03:03/B*15:01	SP across whole region	0.24	2,921
40	62.1	A*02:01/C*03:04/B*15:01			2,941
85	62.1	A*02:01/C*03:04/B*15:01	SP across whole region	0.769	2,990
41	62.1	A*02:01/C*03:04/B*15:01	SR/SP at LINC00243	SR & SP	2,922
49	65.1	A*33:01/C*08:02/B*14:02			2,940
87	65.1	A*33:01/C*08:02/B*14:02	SP from MASIF to MICA	9.1	1,923
78	44.2	A*29:02/C*16:01/B*44:03			2,920
79	44.2	A*29:02/C*16:01/B*44:03	SP from MASIF to MICB	0.847	1,772
83	44.2	A*29:02/C*16:01/B*44:03	SP from HLA-F to MICB	SR & SP	2,975
23	35.5	A*01:01/C*04:01/B*35:02			2,938
45	35.5	A*01:01/C*04:01/B*35:02	SP from HLA-F to MICB	SR & SP	2,938
39	27.1	A*02:01/C*01:02/B*27:05:02			2,943
47	27.1	A*02:01/C*01:02/B*27:05:02	SP from MUC21 to MICB	SR & SP	2,944
24	44.1	A*02:01/C*05:01/B*44:02			2,921
60	44.1	A*02:01/C*05:01/B*44:02	SP from HLA-C to MICB	SR & SP	1,792
74	44.1	A*02:01/C*05:01/B*44:02	SP from HLA-F to MICB	SR & SP	2,937
30	18.x	A*02:01/C*07:01/B*18:01			1,912
33	18.x	A*02:01/C*07:01/B*18:01	SP from HLA-E to MICB	SR & SP	2,929
62	52.1	A*24:02/C*12:02/B*52:01			2,893
93	52.1	A*24:02/C*12:02/B*52:01	SP from HLA-L to MICB	SR & SP	2,887
13	60.1	A*02:01/C*03:04/B*40:01:02			2,749
86	60.1	A*02:01/C*03:04/B*40:01:02	4 SNP crossover regions	SR & SP	2,947
9	44.x	A*32:01/C*05:01/B*44:02			2,937
72	44.x	A*32:01/C*05:01/B*44:02	4 SNP crossover regions	SR & SP	2,976

*For details of sequence alignments between Lab ID and CEH, see Supplementary Table 8*.

### Allelic Lineages Within Alpha Block Haplotypes

Supplementary Table 5 shows the 46 alpha block haplotypes and *HLA-A* and RE allelic lineages of 95 homozygous cell lines (Norman et al., 2017) and the MHC sequence on chromosome 6 of the reference human genome (NC_000006.12, NCBI) using 21 genic and non-genic allelic markers from the telomeric locus of *AluHF* to the centromeric locus of *AluHJ* including *HLA-F, HLA-G*, eight *HLA* pseudogenes and 14 *HLA-A* allelic lineages. Of the *HLA-A* allelic lineages, only seven represented more than three sequence samples: HLA-A^*^01 (*n*, 14), -A^*^02 (*n*, 29), -A^*^03 (*n*, 8), -A^*^24 (*n*, 9), -A^*^29 (*n*, 4), -A^*^30 (*n*, 4), and -A^*^31 (*n*, 4). All seven *HLA-A* haplotype lineages were differentiated by haplotypic and/or haplospecific markers: most of the *Alu, SVA, HERVK9*, and *MER9* within the alpha block (Table 1) were haplotypic and linked to particular *HLA-A* allelic lineages as well as to those of *HLA-F, HLA-G* and the HLA pseudogenes (*HLA*-*V, -P, -H, -T, -K, -U, -W*, and *-J*). Table 2 presents a summary of Supplementary Table 5 and shows the linkages of *AluHF, AluHG, AluHJ*, and *HERVK9* with the *HLA-F, -G, -H, -A* and *-J* alleles in 39 alpha block haplotypes.

(1) All 14 HLA-A^*^01:01:01:01 alleles were linked to the haplospecific *AluHJ* insertion, HLA-J^*^01:01:01:02, HLA-H^*^02:01:01:01, HLA-F^*^01:01:01:09, and the *ERV3-16/(ATAA)*_42_*/(ATTT)*_34_ microsatellite.

(2) Twenty-seven of 28 HLA-A^*^02 haplotype lineages were linked to the haplotypic *AluHG* insertion, the *(CAGAGA)n* microsatellite deletion, the *ERV3-16/(ATAA)*_46_*/(ATTT)*_35_ microsatellite, HLA-G^*^01:01:01:01 and HLA- H^*^01:01:01:01.

(3) A single sequence sample with the HLA-A allele A^*^02:05:01 had no *AluHG* insertion, but had a variant *(CAGAGA)n* microsatellite number, and different alleles for all the alpha block HLA pseudogenes except for HLA-U^*^01:03.

(4) The *AluHG* insertion linked to the *(CAGAGA)n* microsatellite deletion was haplospecific for HLA-A^*^02/ G^*^01:01:01:01/H^*^01:01:01:01, whereas the *AluHJ* insertion was haplotypic for HLA-A^*^01/ G^*^01:01:02:01 or HLA-A^*^24/ G^*^01:01:02:01/G^*^01:04:01:01, respectively.

(5) The *AluHG* insertion with the *(CAGAGA)n* microsatellite deletion was linked also to HLA-A^*^30 in one haplotype and to HLA^*^A31 with the *HERVK9* deletion in another haplotype, but not to the other three with the *HERVK9* insertion, probably as a result of past recombinations or conversions.

(6) The *AluHJ* insertion in the HLA-A^*^01, HLA^*^24 haplotypes and the occasional HLA-A^*^02 or HLA-A^*^03 haplotypes was linked to all of the J^*^01:01:01:02 alleles (25 samples) and to J^*^01:01:01:06 in three samples of the A^*^32:01:01 haplotype.

(7) The *AluHF* was linked to 8 of 14 HLA-F^*^01:01:01:08 alleles, in 2 of 29 HLA-A^*^02 haplotypes, 2 of 3 HLA-A^*^26 haplotypes, all 4 HLA-A^*^29 haplotypes and 1 HLA-A^*^68:02 haplotype.

(8) The *HERVK9* insertion was present in 25 cell lines, whereas the other 70 cell lines had the signatory deletion marker, a solitary *MER9* that is the deletion product of a recombination between the *5*′*MER9* and *3*′*MER9* flanking the 6-kb *HERVK9* internal sequence.

(9) The *HERVK9* insertion was haplotypic for seven of eight HLA-A^*^03/G^*^01:01:01:05/H^*^02:04 samples, all nine HLA-A^*^24 samples, three of four HLA-A^*^31, both HLA-A^*^11 and HLA-A^*^33 samples, and the single HLA-A^*^23 sample.

(10) Both HLA-A^*^11 sequence lineages from the cell lines WT100BIS (Lab ID1) and KGU (Lab ID21) were linked to the *HERVK9* insertion, and to C^*^04:01 and B^*^35:03:01 as extended haplotypes.

(11) All nine HLA-A^*^24:02:01:01 lineages and the single A^*^23:01:01 lineage had a ~55-kb deletion of *HLA-H, SVA-16, HLA-T, HLA-K, LTR13A, HLA-U*, and *sMER9*, ranging from centromeric of the *HERVK9* in the *HLA-H* segment to the *Charlie9* element at the telomeric end of the *MER9* sequence of the *HLA-A* segment (Supplementary Figure 5).

(12) Eight of the nine HLA-A^*^24 haplotypes acquired HLA-J^*^01:01:01:02 with the *AluHJ* insertion, while the other acquired J^*^01:01:01:04 without the *AluHJ* insertion that is similar to the two HLA-A^*^11 haplotypes and the one HLA-A^*^23 haplotype.

### Allelic Lineages Within Beta Block Haplotypes

Supplementary Table 6 shows the genic and non-genic allelic markers for the beta block haplotype sequences of 95 homozygous cell lines from the telomeric locus of *HERVK9/MER9* microsatellite *(TTTC)n* known as *M13* (Kulski et al., 1997) to the centromeric locus of *MICB* including five other microsatellite loci (*M11, M9, Msx, MSa*, and *Msb*), six dimorphic indels (*SVA-HC, SVA-BC, 9.5-kb indel, SVA-HC*, and *AluP5*) and four SNP loci (*HLA-C* and *-B* alleles and *MICA* and *MICB* alleles). There are 12 *HLA-C*, 21 *HLA-B*, 16 *MICA*, and 7 *MICB* allelic lineages that are linked together to form at least 54 *HLA-C/HLA-B/MIC* haplotype lineages. These haplotype lineages were sorted in the sequential order for the absence (allele 1) and presence (allele 2) of the *SVA-HB* insertion, and the alleles of *HLA-C, HLA-B, MICA*, and *MICB*, respectively (Table 3). The *SVA-HB* insertion (allele 2) is missing from the chimpanzee and gorilla MHC (data not shown), and its absence is assumed to be the ancestral allele.

Fifty-six of the 95 sequenced cell lines had the *SVA-HB* insertion. The *HLA-C* haplotypic lineages with no *SVA-HB* insertion were 6 C^*^01:02:01, 1 C^*^03:04:01 linked to *HLA*-B^*^40:01:02, 10 C^*^05, 2 C^*^07:01, 8 C^*^07:02, 3 C^*^08, 1 C^*^12:02 linked to HLA-B^*^52, 2 C^*^14 and 3 C^*^15. The *HLA-C* lineages with the *SVA-HB* insertion were 2 C^*^01:02:01 linked to HLA-B^*^27, 3 C^*^02, 9 C^*^03, 9 C^*^09, 11 C^*^06, 6 C^*^07:01 and 1 C^*^07:18, 9 C^*^12, 4 C^*^16, and 2 C^*^17. Only C^*^01, C^*^03 and C^*^07 had crossed over to be represented by both the absence and presence of the *SVA-HB*. Eighteen of 56 *SVA-HB* positive sequences contained *SVA-HB* duplications and *LTR10/HERVI/LTR10* rearrangements that did not correlate with any particular *HLA-C* lineage or haplotype, suggesting that these variants were likely sequencing assembly errors. Nevertheless, there are two distinct haplotype evolutionary histories for the beta block that are based on the absence or presence of the *SVA-HB* insertion.

The *SVA-HC* insertion was specific for the eight HLA-C^*^07:02:01/B^*^07:02:01/MICA^*^008:04/ MICB^*^008:04 haplotypes, whereas the *SVA-BC* insertion was linked to six C^*^01:02 samples with various *HLA-B* alleles, three of four C^*^07:01 alleles and both C^*^14 alleles (Table 3, Supplementary Table 1). The *SVA-BC* and the *SVA-HC* insertions were present only in samples without the *SVA-HB* insertion. In comparison, the *SVA-MIC* insertion was linked to various *HLA-B* alleles both with and without linkage to the *SVA-HB* insertion. The *(ACACAT)*_101_ and the *(ACACAT)*_161_ simple repeats located between *HLA-C* and *HLA-B* further subdivide these *HLA-B* haplotypic lineages (data not shown). Three different haplotype families with *(ACACAT)*_101_ had no *SVA-HB* insertion, three HLA-B^*^14, seven HLA-B^*^18 and five of nine HLA-B^*^44 (Supplementary Table 6). The five lineage haplotypes with the microsatellite *(ACACAT)*_161_, but without the *SVA-HB* insertion, were B^*^07, B^*^46, B^*^51, B^*^54 and B^*^56. Single examples of B^*^15, B^*^40, B^*^44, B^*^52 and B^*^57 with *(ACACAT)*_161_ were either with or without *SVA-HB* (Supplementary Table 6). The different *HLA-B* lineage haplotypes with the *SVA-HB* insertion were partitioned further into another two lineages: those with the 9-kb deletion between *AluY-(AT)n* and *AluJb-(TTAT)n* and those without the 9-kb deletion (Table 3, Supplementary Figure 1). The 18 *HLA-B* haplotypes with the 9-kb deletion were all linked to either HLA-C^*^06:02:01 or -C^*^12:03:01 (Table 3).

### Segmental Exchanges and SNP XO Within the MHC Class I Region

**Figure 1:**
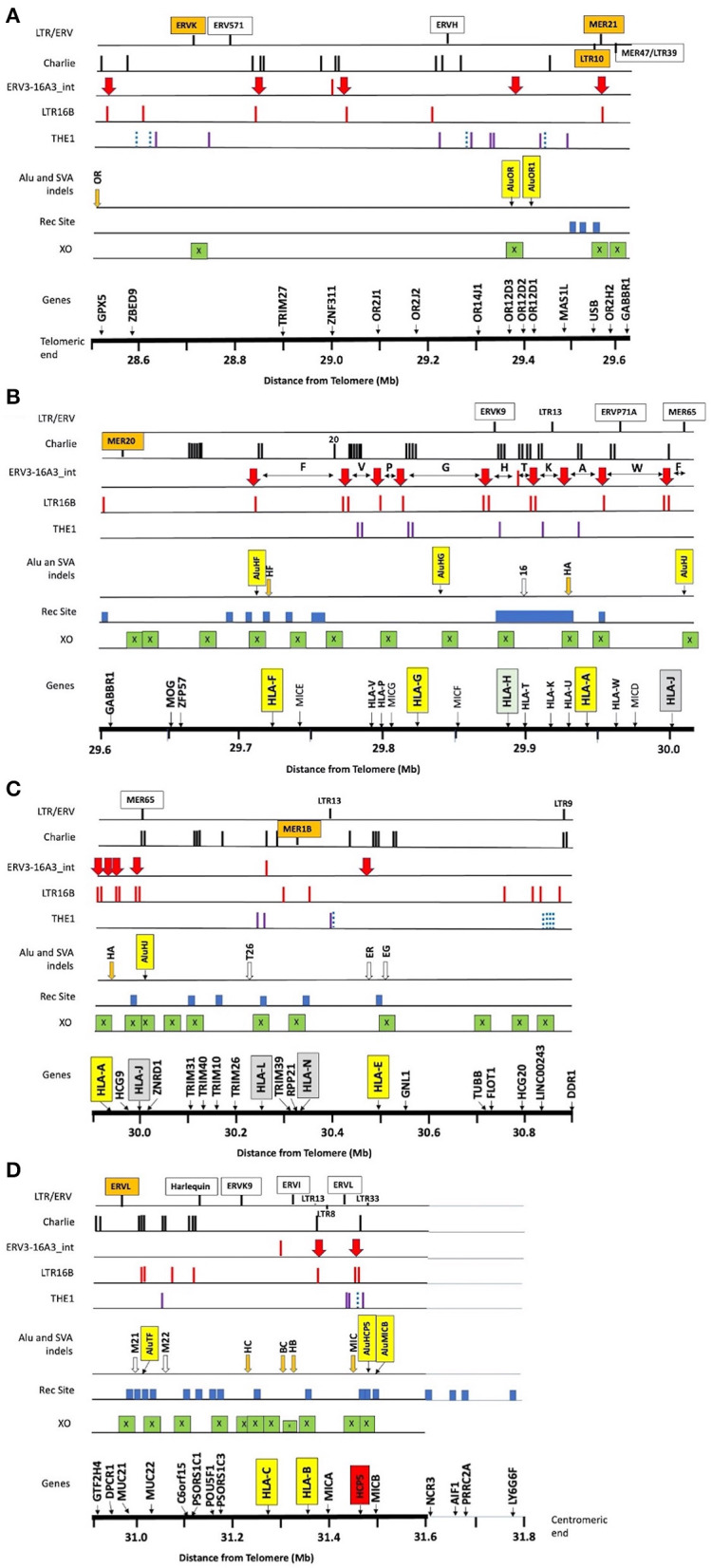
Summary of the locations of gene markers (Genes), 38 crossover sites (XO), 31 recombination sites (Rec Site), 8 *Alu* and 13 *SVA* indels, and the repeat elements *THE1, LTR16, ERV3-16A3_in*t (aka *HERV16*), *Charlie*, and other labelled LTR/ERV elements within ~3 Mb of OR/MHC class I genomic sequence from *GPX5* to *MICB* and the nucleotide position 28.5–31.6 (Mb distance from telomere on chromosome 6, GRCh38.p12 Primary Assembly NC_000006.12; NCBI, UCSC or ENSEMBL browsers on the Web). **(A)** The OR genomic region from 28.5 to 29.6 Mb, **(B)** the OR and MHC alpha block region from 29.6 to 30 Mb with the location of the 10 HLA segmental duplications segment F to segment J indicated by the horizontal double arrows between the *ERV3-16A3_int* insertions (solid red vertical arrow), **(C)** the *TRIM* gene cluster, *HLA-L* and *HLA-N* pseudogenes, non-classical *HLA-E* gene, and structural and regulatory gene marker (*TUBB* to *DDR*1) from 29.9 to 30.9 Mb, **(D)** the regulatory gene region (*GTF2H4* to *PSORS1C3*) and the beta block of *HLA-C* to *MICB* from 29.9 to 31.6 Mb. The *NCR3* to *LY6G6F* gene markers from 31.6 to 31.8 Mb are near the putative recombination sites identified by Lam et al. (2013) at the start of the MHC class III region. The X within green boxes indicate crossover sites, the blue solid blocks are the recombination sites reported by Lam et al. (2013), and the vertical orange (indels) and open arrows (fixed) are the *SVA* listed in Table 1. The Alu indels such as *AluOR* and *AluOR1* in Table 1 are indicated as labelled yellow vertical boxes in the Alu or SVA indel row. The location of the *THE1, LTR16B*, and *Charlie* are indicated by the vertical bars in their respective rows. The blue dotted bars represent the *THE1B* subfamily and the purple vertical bars represent the other subfamilies, *THE1A, THE1C*, and *THE1D*.

Supplementary Table 7 shows 39 examples of segmental shuffling between HLA class I genes *A, B, C* and *E*, pseudogene *HLA-J* and the *MICA* and *MICB* genes of 59 different representative AH and subtypes using *HLA-B* alleles as AH anchor points. Most of the MHC haplotypes within the homozygous cell lines are Caucasoids from Europe, North America, South Africa and Australia. The exceptions are one cell line from a North American Hispanic (MGAR), five Oriental cell lines (SA, ISH3, HOR, AKIBA, and KAWASAKI) and five South American Indian cell lines (LZL, AMALA, SPL, RML, and KRC005). The four RE dimorphic structural markers *AluHG, AluHJ*, and *HERK9* within the alpha block and *SVA-HB* within the beta block further subdivided some of these AH. It is noteworthy that the Sardinian 18.2AH, HLA-A^*^30:C^*^05:B^*^18 (Contu et al., 1989, Bilbao et al., 2006) in the cell lines EJ32B and DUCAF has two specific dimorphic Alu insertions, *AluOR* and *AluOR1* (Supplementary Table 2), located ~300 kb from the *HLA-F* gene (Figure 1). This finding confirms that the CPS of some MHC class I haplotypes and AH like the *18.2AH* extend well into the *OR* gene cluster telomeric of the *HLA-F* gene and the MHC alpha block by at least 1,185 kb. The *AluOR* insertion was found also in one of two HLA-A^*^29 sequenced samples (cell lines PITOUT and MOU, respectively), the HLA-A^*^02:05:01 cell line WT49, the HLA-A^*^11:01 cell line WT100BIS and the HLA-A^*^23:01 cell line WT51 (Supplementary Table 2).

The four Warao South American Indian cell lines LZL, AMALA, SPL and RML (Supplementary Tables 1, 2) present an interesting ethnic contrast to the Caucasoid cell lines (Supplementary Table 7). The Warao people who inhabit the rainforests of Orinoco Delta of northeastern Venezuela and western Guyana are an ancient ethnic minority with an extant population of ~50,000 people. The Warao *62.xAH* and *51.xAH* have the *HLA-A* alleles A^*^2:17:02, A^*^02:04, and A^*^02:12 rather than the common Caucasoid A^*^02:01:01, but they also carry the *AluHG* insertion that is linked to most of the Caucasoid A^*^02 lineages (Table 2). One of the Warao cell lines (SPL, ID73) has the HLA-A^*^31:02:02 allele linked to the *HERVK9* insertion and *SVA-HB* deletion, which is markedly different to the Caucasoid and Oriental *62.xAHs*, but with an alpha block haplotypic structure similar to two Caucasoid HLA-A^*^31:02 lineages represented by the English cell line JHAF (ID46) and the Australian cell line MT14B (ID82) (Supplementary Tables 1, 2). The other HLA-A^*^31:02 haplotype represented by the European Caucasoid cell line DEU (ID35) is with an *AluHG* insertion, a *HERVK9* deletion (Table 2) and an *SVA-HB* insertion (Table 3), suggesting that a more modern HLA-A^*^31 AH was subsequently generated by segmental shuffling exchanges.

Since the exact SNP XO regions between different MHC haplotypes are poorly defined in regard to the intergenic and genic distribution of repeat elements, a detailed comparative examination of DNA sequence alignments of similar and different haplotypes was undertaken using the PIP method of Schwartz et al. (2000). We started with an examination of ~3 Mb of genomic sequence of the same haplotypes that included the class I region from *HLA-F* to *MICB* and the *OR* gene cluster that included the *GPX5, ZNF311, OR2H2, GABBR1*, and *MOG* genes (Table 4, Supplementary Table 8). We then performed a more detailed examination of SNP densities and XOs within the 1.2-Mb *OR* gene region (Table 5), the 310-kb alpha block (Table 6), the 1,172-kb inter alpha and beta blocks (Supplementary Tables 9, 10) and the 307-kb beta block (Tables 7, 8) of the same and/or different *HLA-A, -C* and *-B* haplotypes. The alignments and SNP counts were analysed manually across the entire 3 Mb and in 50-kb to 500-kb segments connecting the various segments as a sliding window. Figure 1 summarises the findings of our analysis of more than 250 sequence alignments between different and the same haplotypes with the identification of at least 38 ancestral SNP XO sites between SNP-poor and SNP-rich regions within ~2.8 Mb from *GPX5* to *MICB*.

**Table 5:** SNP crossover (XO) loci in the extended MHC class I OR gene region.

**Haplotype sequence alignment**			**OR GENE CLUSTER REGION/GABBR1/MOG**				**Total SNPs**	**XO point in haplotype sequence ID_HAP1**	**Within or between (/) RE**	**XO between (/) genes**
		**GPX5 to OR2H1**	**MAS1L & LINC01015**	**USB & OR2H2**	**GABBR1**	**MOG & ZFP57**				
		**AA**	**A**	**B**	**C**	**D**	**A-D**			
**ID_HAP1**	**ID-HAP2**	**960 kb**	**SNP/50 kb**	**SNP/50 kb**	**SNP/50 kb**	**SNP/60 kb**	**SNP/210 kb**			
49_A*33-C*08:02	50_A*33-C*14:03	SRR	87	56	17	216 (XO)	376	208524 A/G	ERV3-16A3	F segment
15_A*26-C*05	64_A*26-C*12:03	SRR	39	44	21	214 (XO)	318	211345 A/G	ERV3-16A3	F segment
15_A*26-C*05	69_A*66-C*12:03	SRR	45	34	11	19 (XO)	109	210115 G/A	LTR43/ERV3	F segment
2_A*01-C*06	11_A*01-C*07	SRR	105	68	26	2 (XO) 1	202	165747 T/C	Charlie4/L3b	ZFP57/HLA-F
2_A*01-C*06	16_A*01-C*07	SRR	93	64	21	2 (XO) 1	181	165747 T/C	Charlie4/L3b	ZFP57/HLA-F
4_A*03-C*07:02	7_A*03-C*06:02	SRR	97	43	4 (XO)	3	147	121130 A/C	L3b/MER5	GABBR1/MOG
9_A*32-C*05	22_A*32-C*12:03	SRR	90	51	17(XO) 0	2	160	143183 T/C	AluY/L2	GABBR1/MOG
9_A*32-C*05	72_A*32-C*05	SRR	91	49	15 (XO) 0	2	157	143183 T/C	AluY/L2	GABBR1/MOG
10_A*02-C*12:03	17_A*02:17-C*03	SRR	93	30 (XO) 0	2	5	130	92214 G/A	AluY(Sc8)	OR2H2/GABBR1
10_A*02-C*12:03	92_A*02:12-C*01	SRR	91	30 (XO) 0	2	3	126	92214 G/A	AluY(Sc8)	OR2H2/GABBR1
5_A*02-C*05	10_A*02-C*12:03	SRR	77	32 (XO) 0	0	2	111	75758 T/C	MER21C	USB/OR2H2
10_A*02-C*12:03	5_A*02-C*05	SRR	77	32 (XO) 0	0	2	111	75792 C/T	MER21C	USB/OR2H2
34_A*24-C*03:04	68_A*24-C*04	SRR	97	15 (XO) 0	1	0	113	59547 C/T	LTR10A	USB/OR2H2
46_A*31-C*15:02	73_A*31-C*01:02	SRR	58	9 (XO) 2	2	6	75	59532 T/C	LTR10A	USB/OR2H2
15_A*26-C*05	84_A*26-C*07	SRR	37	20 (XO) 1	2	0	59	66738 A/T	Tigger2b-Pri	USB/OR2H2
22_A*32-C*12:03	72_A*32-C*05	(XO)	2	1	4	0	7	136748 T/C	L2/L1	OR12D3/OR12D2
4_A*03-C*07:02	6_A*03-C*07:02		6_missing seq	4	0	0	4	Undetected		SPR from block AA to block D
78_A*29-C*16	79_A*29-C*16		79_missing seq	0	0	1	1	Undetected		SPR from block AA to block D
11_A*01-C*07	16_A*01-C*07	7	0	0	1	0	1	Undetected		SPR from block AA to block D
4_A*03-C*07:02	51_A*03-C*07:02	8	0	0	0	1	1	undetected		SPR from block AA to block D
34_A*24-C*03:04	94_A*24-C*03:04	0	0	0	2	1	3	Undetected		SPR from block AA to block D
25_A*30-C*05	26_A*30-C*05	0	0	1	0	0	1	Undetected		SPR from block AA to block D
46_A*31-C*15:02	82_A*31-C*03:04	0	0	2	1	4	7	Undetected		SPR from block AA to block D
15_A*26-C*05	78_A*29-C*16	SRR	45	37 (XOa) 0	3	4 (XOb)	89	91066 G/A	L2/MS	OR2H2/GABBR1
2_A*01-C*06	17_A*02:17-C*03	SRR	11	58	48	197	314			
25_A*30-C*05	34_A*24-C*03:04	SRR	49	40	85	191	365			
25_A*30-C*05	46_A*31-C*15:02	SRR	49	35	27	287	398			
25_A*30-C*05	22_A*32-C*12:03	SRR	83	42	13	51	189			

*SRR is SNP-rich region, SPR is SNP-poor region, XO is crossing over, and numbers in the block columns AA to A–D are SNP counts per block*.

**Table 6:** SNP counts and crossovers (XO) within alpha block segments 1 to 10 between different haplotype sequence alignments (ID_Hap1 and ID_Hap 2).

**Segment number**			**1**	**2**	**3**	**4**	**5**	**6**	**7**	**8**	**9**	**10**	**1 to 10**		
**Segment name**		**OR end**	**F**	**V**	**P**	**G**	**H**	**T**	**K**	**A**	**W**	**J**	**F to J**	**Position and XO SNP**	**XO within or between (/) RE**
**Segment size**		**2.2 kb**	**57 kb**	**25 kb**	**23 kb**	**53 kb**	**23.4 kb**	**20.4 kb**	**19 kb**	**15 kb**	**51 kb**	**34 kb**	**320.8**		
**Hap sequence comparisons**															
**ID_Hap 1**	**ID_Hap 2**														
11_A^*^01:01	4_A^*^03:01	12	284	134	198	382	340	330	245	107	180	40	2,240		
2_A^*^01:01	4_A^*^03:01	11	280	144	200	388	367	374	248	110	187	38	2,336		
10_A^*^02:01	4_A^*^03:01	12	262	32	35	133	328	321	248	343	380	30	2,112		
2_A^*^01:01	10_A^*^02:01	9	340	135	194	371	103	55	164	346	380	35	2,123		
5_A^*^02:01	1_A^*^11:01	15	280	35	30	517	356	259	80	240	211	27	2,035		
15_A^*^26:01	78_A^*^29	0	4 XO 30	137	182	208	69	203	232	148	727	217	2,157	[F] 56590 G/C	Charlie20a
25_A^*^30	46_A^*^31	13	206	111	152	264	355	260	297	335	491	173	2,644		
**SNP density**	**average: SNPs/kb**	**4.7**	**4.2**	**4.2**	**6.2**	**6.1**	**11.7**	**12.6**	**11.4**	**15.5**	**7.2**	**2.4**	**7.0**		
4_A^*^03:01	46_A^*^31	13	SRR	SRR	SRR	SRR	SRR	SRR	SRR	SRR	SRR	SRR	SRR		
4_A^*^03:01	49_A^*^33	2	279	191	166	236	65	106	268	331	476	169	2287		
46_A^*^31	49_A^*^33	15	245	196	164 XO 0	16	7	9	11	11	33	15	707	[P] 97693 C/T	MICG
4_A^*^03:01	48_A^*^24/B^*^15:26N	11	255	18	27	502	92/XO/del	del	del	del/XO/175	171	22	1262	[H] 168684 del	L1/AAAGA/MLT1F1
4_A^*^03:01	94_A^*^24	10	279	135	194	440	91/XO/del	del	del	del/XO/175	219	38	1571	[H] 168684 del	L1/AAAGA/MLT1F1
5_A^*^02:01	8_A^*^02:05	11	268	106	156	371	27 XO	14	9	Assembly errors		28	952	[H] 168492 G/C	L2/HLA-H/L2
10_A^*^02:01	8_A^*^02:05	11	SRR	SRR	SRR	SRR	SRR XO	5	7	13	XO 199	24	SRR/SPR	[H] 168959 G/C	L2/HLA-H/L2
10_A^*^02:01	8_A^*^02:05									13	XO 199	24	SPR/SRR	[W] 224700 C/T	ERV3-16A3
34_A^*^24	48_A^*^24	9	73	139	200	229/XO/0	1/XO/del	del	del	del/XO/2	82	39	763	[G] 141380 A/G	HAL1/MICF
34_A^*^24	59_A^*^24	2	132	141	199	229/XO/1	0/XO/del	del	del	del/XO/2	5	1	710	[G] 141380 A/G	HAL1/MICF
48_A^*^24	68_A^*^24	9	86	132	199	331/XO/1	0/XO/del	del	del	del/XO	89	41	879	[G] 144028 G/A	HAL1/MICF
48_A^*^24	94_A^*^24	9	88	132	199	334/XO/1	0/XO/del	del	del	del/XO	88	42	884	[G] 144028 G/A	HAL1/MICF
59_A^*^24	94_A^*^24	2	132	136	197	284/XO/1	3/XO/del	del	del	del/XO	4	1	760	[G] 141500 G/A	HAL1/MICF
10_A^*^02:01	13_A^*^02:01	10	171+XO	0	0	3	2	1	1	0	0	0	178	[F] 53540 G/T	Charlie20a
10_A^*^02:01	67_A^*^02:04	10	155+XO	1	1	2	2	0	0	1	0	0	152	[F] 53540 G/T	Charlie20a
10_A^*^02:01	76_A^*^02:04	10	155+XO	1	1	2	2	0	0	1	0	0	152	[F] 53540 G/T	Charlie20a
5_A^*^02:01	13_A^*^02:01	11	166+XO	0	0	6	1	3	3	Assembly errors		3	7	[F] 53275 G/T	Charlie20a
4_A^*^03:01	18_A^*^03/A^*^24	5	137	78	126	179+XO	0	0	2	7/XO	131	37	692	[G} 150337 C/T	Tigger1/Charlie20a
														[A] 229198 G/T	L2/HLA-A/L2
48_A^*^24	59_A^*^24	11	139 XO 0	1	0	0	0/XO/del	del	del	del/XO	87	42	269	[F} 53185 T/C	Tigger1/Charlie20a
34_A^*^24	68_A^*^24	0	0	0	1	1	XO/del	del	del	del/XO	1	0	2	[H] del [A]	
34_A^*^24	94_A^*^24	0	0	0	1	1	XO/del	del	del	del/XO	0	0	1	[H] del [A]	
10_A^*^02:01	32_A^*^02:17	Seq missing		0	1	4	1	0	0	3	0	0	9	OR cluster	
5_A^*^02:01	10_A^*^02:01	1	3	0	0	5	3	2	2	Assembly errors		3	18	OR cluster	
11_A^*^01:01	16_A^*^01:01	0	0	0	0	0	0	0	0	0	1	21.5 kb del	1	OR cluster	
2_A^*^01:01	16_A^*^01:01	0	1	1	0	1	3	3	3	32	3	21.5 kb del	47	OR cluster	
2_A^*^01:01	11_A^*^01:01	0	1	1	0	1	3	3	3	1	3	2	18	OR cluster	
10_A^*^02:01	17_A^*^02:17	0	0	0	1	2	1	0	0	3	0	0	7	OR cluster	
10_A^*^02:01	92_A^*^02:12	0	1	0	0	1	0	0	1	2	1	0	6	OR cluster	
4_A^*^03:01	6_A^*^03:01	0	1	0	0	0	1	0	0	0	1	0	3	OR cluster	
4_A^*^03:01	7_A^*^03:01	0	0	0	1	0	0	1	1	0	2	1	6	OR cluster	
15_A^*^26	64_A^*^26	0	1	0	1	2	3	0	1	0	4	1	13	OR cluster	
15_A^*^26	84_A^*^26	0	1	0	0	3	1	0	0	0	2	0	7	OR cluster	
15_A^*^26	69_A^*^66	2	10	3	1	15	7	6	1	3	9	8	63	OR cluster	
78_A29	79_A29	0	0	0	0	1	0	0	0	0	0	0	1	OR cluster	
25_A^*^30	26_A^*^30	0	0	0	0	1	0	0	0	0	0	0	1	OR cluster	
46_A^*^31	73_A^*^31	1	1	1	0	0	1	0	0	0	1	0	4	OR cluster	
46_A^*^31	82_A^*^31	1	0	0	0	0	0	0	0	0	0	1	1	OR cluster	
9_A^*^32	22_A^*^32	0	0	0	0	0	0	0	0	0	0	0	0	OR cluster	
9_A^*^32	72_A^*^32	0	0	1	0	1	1	2	1	0	1	1	8	OR cluster	
49_A^*^33	50_A^*^33	0	0	0	0	1	0	0	0	0	0	0	1	OR cluster	

* *The SRR to SPR XO in Seg G (4) at A/G 141380 HAL1 and (ATAAT)n is near the AluHG insertion locus and the MICF pseudogene. XO is the abbreviation for crossover; SRR, SNP-rich region; SPR, SNP-poor region. The numbers before and/or after XO are the number of counted SNPs before and/or after the observed XO. There are XO points outside the alpha block in the telomeric OR gene region and the centromeric non-HLA region between HLA-J and HLA-E. The bold values here show the SNP density and the average number of SNPs/kb for the top 7 haplotype sequence comparisons in the table*.

**Table 7:** SNP counts and crossover (XO) loci between linked HLA-C and HLA-B alleles using different combinations of haplotype pairs.

**Alignments between haplotypes**		**Number of SNPs per section**								**XO**	**SNP or**	**closest**	**XO nt distance**	**XO in or**
**Lab ID numbers precede haplotypes**		**0–7 k**	**7–10 kb *HLA-C***	**10–20 k**	**20–40 k**	**40–60 k**	**60–80 k**	**80–93 k *HLA-B***	**0–93 k**	**Location bp**	**Indel at XO**	**Repeat**	**To end of HLA-B exon 8**	**Between (/) HLA-C and -B**
**Haplotype 1**	**Haplotype 2**													
**(A) Different HLA-C/HLA-B haplotypes**														
66_C*02/B*27:05	49_C*08:02/B*14:02	33	32	85	114	455	805 + MI	269	1,811	none	0	nd	0	SRR
55_C*06:02/B*13:02	95_C*07/B*57	15	88	85	112	247 + MI	768	185	1,500	none	0	nd	0	SRR
**(B) Same HLA-C/ HLA-B haplotypes**														
28_C*03:03/B*15	17_C*03:03/B*15	0	0	0	1	0	2	2	5	0	0	nd	0	SPR
54_C*04/B*35	23_C*04/B*35	0	0	0	1	1	0	0	2	0	0	nd	0	SPR
55_C*06:02/B*13:02	91_C*06:02/B*13	0	0	1	0	0	0	0 + indels	1	0	0	nd	0	SPR
6_C*07/B*07:02	4_C*07/B*07:02	0	0	0	0	0	0	0	0	0	0	nd	0	SPR
11_C*07/B*08	12_C*07/B*08	0	0	0	0	0	0	0	0	0	0	nd	0	SPR
65_C*08:02/B*14:01	49_C*08:02/B*14:02	0	0	0	0	0	0	77	77	0	0	nd	0	SPR
**(C) Same HLA-C allele–different HLA-B allele**														
6_C*07/B*07:02	30_C*07/B*18	12	9 + XO	68	89	SRR	SRR	SRR	SRR	HLA-C*7	0	nd	nd	HLA-C
30_C*07/B*18	6_C*07/B*07:02	12	9 + XO	68	89	SRR	SRR	SRR	SRR	HLA-C*7	0	nd	nd	HLA-C
6_C*07/B*07:02	8_C*07:18/B*58	19	9 + XO	122	163	SRR	SRR	SRR	SRR	HLA-C*7	0	nd	nd	HLA-C
8_C*07:18/B*58	6_C*07/B*07:02	19	9 + XO	122	163	SRR	SRR	SRR	SRR	HLA-C*7	0	nd	nd	HLA-C
94_C*12:03/B*51	56_C*12:02/B*52	41	XO + 24	76	195	SRR + MI	SRR	41	SRR	HLA-C*12	0	nd	nd	HLA-C
94_C*12:03/B*51	53__C*12:02/B*52	41	2 + XO	70	217	SRR	SRR	41	SRR	HLA-C*12	0	nd	nd	HLA-C
11_C*07/B*08	8_C*07:18/B*58	169	4	2+XO+2	163	32	277 + MI	387	1036	19961	A/G	L1PA13	72,286	HLA-C/HLA-B
11_C*07/B*08	30_C*07/B*18	159	0	0+XO+2	91	327 + MI	348 + MI	341	1268	19961	A/G	L1PA13	72,286	HLA-C/HLA-B
11_C*07/B*08	42_C*07/B*49	159	0	0+XO+2	89	329 + MI	348 + MI	376	1303	19961	A/G	L1PA13	72,286	HLA-C/HLA-B
11_C*07/B*08	95_C*07/B*57	1	0	0+XO+1	91	327 + MI	348 + MI	373	1141	19961	A/G	L1PA13	72,286	HLA-C/HLA-B
92_C*01/B*51	39_C*01/B*27:05	0	0	1	XO+30	367	578 + 5k MI	240	1216	33326	C/T	MIR/L1MB8	55,903	HLA-C/HLA-B
39_C*01/B*27:05	92_C*01/B*51	0	0	1	XO+SRR	SRR	SRR	SRR	SRR	32511	C/T	MIR/L1MB8	60,958	HLA-C/HLA-B
39_C*01/B*27:05	70_C*01/B*46	2	1	2	XO+SRR	SRR	SRR	SRR	SRR	35360	T/A	AluY/MLT1D	58,109	HLA-C/HLA-B
39_C*01/B*27:05	89_C*01/B*54	26	0	2	XO+SRR	SRR	SRR	SRR	SRR	35360	T/A	AluY/MLT1D	58,109	HLA-C/HLA-B
39_C*01/B*27:05	59_C*01/B*56	2	0	2	XO+SRR	SRR	SRR	SRR	SRR	35360	T/A	AluY/MLT1D	58,109	HLA-C/HLA-B
39_C*01/B*27:05	73_C*01/B*15	1	1	2	XO+SRR	SRR	SRR	SRR	SRR	35360	T/A	AluY/MLT1D	58,109	HLA-C/HLA-B
11_C*07/B*08	4_C*07/B*07:02	158	7	67	0	0 + XO +217	SRR + MI	SRR	SRR	47841	C/T	MIR	44,406	HLA-C/HLA-B
11_C*07/B*08	6_C*07/B*07:02	158	7	67	0	0 + XO + 217	SRR + MI	SRR	SRR	47841	C/T	MIR	44,406	HLA-C/HLA-B
55_C*06:02/B*13:02	31_C*06:02/B*40	0	0	0	0	0	XO+529	210	739	60351	G/T	HERVI	21,449	HLA-C/HLA-B
5_C*05/B*18	9_C*05/B*44:02	0	0	0	1	0	0	XO+87	88	89798	T/C	MLT1N2	3,827	3′HLA-B
95_C*07/B*57	30_C*07/B*18	160	0	1	0	1	2	XO+147	319	87226	A/C	MLT1N2/MER5	2,767	3′HLA-B
28_C*03:03/B*15	13_C*03:04/B*40:01	5	1	2	2	5	3	XO+184	204	88616	indel 36bp	MLT1N2/MER5	2,478	3′HLA-B
28_C*03:03/B*15	34_C*03:04/B*40:01	5	2	2	2	5	MI + 3	XO+185	205	88616	indel 36bp	MLT1N2/MER5	2,478	3′HLA-B
54_C*04/B*35	48_C*04/B*15:26N	0	0	MI + 0	1	0	17	XO+152	170	88190	C/T	MLT1N2/MER5	2,440	3′HLA-B
54_C*04/B*35	61_C*04/B*53	0	0	1	1	0	1	XO+7	10	88194	T/C	MLT1N2/MER5	2,436	3′HLA-B
50_C*14:03/B*44:03	88_C*14:02/B*51	0	0	2	6	1	2	5+XO+158	174	99804	C/A	MLT1N2/MER5	2,273	3′HLA-B
30_C*07/B*18	95_C*07/B*57	160	0	1	0	1	2	XO+147	319	93101	A/C	MLT1N2/MER5	2,268	3′HLA-B
94_C*12:03/B*51	22__C*12:03/B*38	0	0	0	0	0	0	XO+182	182	80294	G/A	MLT1N2/MER5	2,226	3′HLA-B
66_C*02:02/B*27:05	38_C*02/B*40:02	0	0	1	0	0	0	XO+33	34	88896	indel 5bp	MLT1N2/MER5	2,165	3′HLA-B
55_C*06:02/B*13:02	77_C*06:02/B*47	0	0	0	0	1	0	XO+119	120	80217	G/C	L2	1,583	3′HLA-B
55_C*06:02/B*13:02	36_C*06:02/B*57	0	0	0	0	0	2	XO+187	189	81180	G/A	L2	620	3′HLA-B
55_C*06:02/B*13:02	2_C*06:02/B*57	0	0	0	0	0	3	XO+187	190	81180	G/A	L2/HLA-B	620	3′HLA-B
55_C*06:02/B*13:02	43_C*06:02/B*37	0	0	0	0	0	3	XO+190	193	81180	G/A	L2/HLA-B	620	3′HLA-B
92_C*01/B*51	73_C*01/B*15	0	0	1	0	0	0	XO+125	126	88668	G/A	L2/HLA-B	560	3′HLA-B
92_C*01/B*51	59_C*01/B*56	0	0	1	0	0	1	XO+241	242	88668	G/A	L2/HLA-B	560	3′HLA-B
92_C*01/B*51	70_C*01/B*46	0	0	1	1	0	0	XO+127	129	88668	G/A	L2/HLA-B	560	3′HLA-B
92_C*01/B*51	89_C*01/B*54	18	0	0	0	0	0	XO+168	207	88787	indel 22bp	L2/HLA-B	441	3′HLA-B
83_C*16/B*44:03	80_C*16/B*45	0	0	0	2	0	2	XO+133	137	91097	indel	L2/HLA-B	503	3′HLA-B
29_C*17/B*41	14_C*17/B*42	0	0	0	1	0	2	XO+205	208	86647	G/A	HLA-B exon 3	−2,542	HLA-B (ex 3)
**(D) Different HLA-C allele/same HLA-B allele**														
92_C*01/B*51	94_C*12/B*51			176	132	79 + MI	791 + MI	190 + XO	1368	89594	G/A	HLA-B exon 8	−186	HLA-B exon 8
5_C*05/B*18	30_C*07/B*18			SRR	SRR	SSR + MI (5.2kb)	SRR	SRR +XO	SRR + MI	93767	G/A	HLA-B exon 8	−142	HLA-B exon 8
9_C*05/B*4402	83_C*16/B*4403			SRR	SRR	SRR	SRR + MI	SRR +XO	SRR + MI	92080	C/A	L2/HLA-B	139	3′HLA-B
92_C*01/B*51	46_C*15/B*51			141	295	222	149	140 + XO	947	89065	A/T	L2/HLA-B	163	3′HLA-B
9_C*05/B*4402	50_C*14/B*4403			SRR	SRR	SRR + MI	SRR + MI	SRR +XO	SRR + MI	91971	T/C	L2/HLA-B	248	3′HLA-B
73_C*01/B*15	28_C*03/B*15			SRR	SRR	SRR	SRR + MI	SRR + XO	SRR + MI	87246	A/G	L2/HLA-B	640	3′HLA-B
73_C*01/B*15	48_C*04/B*1525N			SRR	SRR	SRR	SRR + MI	SRR + XO	SRR + MI	87246	A/G	L2/HLA-B	640	3′HLA-B
2_C*06/B*57	95_C*07/B*57			SRR	SRR	SRR + MI	SRR	SRR +XO	SRR + MI	83390	A/G	L2/HLA-B	650	3′HLA-B
54_C*04/B*35	20_C*12/B*35			SRR + MI	SRR + MI	SRR + MI	SRR	SRR +XO	SRR + MI	89662	C/A	L2	968	3′HLA-B
39_C*01/B*27:05	66_C*02/B*27:05			175	79+XO+0	0	0	0	254	35005	T/A	L1MB8/MLT1D	58523	HLA-C/HLA-B
38_C*02/B*40:02	34_C*03/B*40:02			SRR	SRR	SRR	SRR + MI	SRR	SRR	nd	0	nd	nd	SRR
38_C*02/B*40:02	77_C*06/B*40:01			SRR	SRR	SRR + MI	SRR	SRR	SRR	nd	0	nd	nd	SRR

*SRR is SNP-rich region that is estimated to be >100 SNP without manual counts, SPR is SNP-poor region (<10 SNP), and MI is major indel (>1 kb usually <6 kb). XO in columns is crossover and XO + number presents the number of SNPs before or after the crossover in each of the 20-kb genomic sections. nd, not determined*.

**Table 8:** SNP crossover (XO) loci within intergenic regions between *HLA-B* and *MICA* or *MICB* in alignments of different haplotype pairs.

**Alignments between paired haplotype sequences**		**XO distance from HLA-B**	**XO**	**XO within gene or within and between (/) repeat elements**
**Lab ID numbers precede haplotypes**			**SNP**	
**Haplotype 1**	**Haplotype 2**	**SPR/SRR**		
65_B*14:01/MICA*019/MICB*502	49_B*14:02/MICA*011/MICB*502	8796	T/C	L1
65_B*14:01/MICA*019/MICB*502	87_B*14:01/MICA*11/MICB*005:02	8822	G/A	L1
72_B*44/MICA*008/MICB*005	83_B*44:03/MICA*004/MICB*005	14436	A/G	L1M5/L1ME3
72_B*44/MICA*008/MICB*005	50_B*44:03/MICA*004/MICB*005	14436	A/G	L1M5/L1ME3
25_B*18/MICA*001MICB*5002	30_B*18/MICA*018/MICB*201	16711	G/T	L1
19_B*08/MICA*008/MICB*008	84_B*08/MICA*008/MICB*004	30243	C/T	MLT2C1/Charlie9
94_B*51/MICA*006/MICB*005	92_B*51/MICA*010/MICB*005	44271	A/G	LTR8A
94_B*51/MICA*006/MICB*005	46_B*51/MICA*009/MICB*002	44618	C/T	LTR8A/AluJb
94_B*51/MICA*006/MICB*005	67_B*51/MICA*009/MICB*005	52042	G/A	(CTC)n/L1M3
88_B*51/MICA*049/MICB*005	92_B*51/MICA*010/MICB*005	54075	A/G	LTR8A
54_B*35/MICA*002/MICB*005	68_B*35/MICA*016/MICB*002	66656/MICA	C/T	L1MB2/MIR in MICA
46_B*51/MICA*009/MICB*002	67_B*51/MICA*009/MICB*005	95787	C/T	L1PA3/Tigger3b
86_B*40/MICA*008/MICB*002	82_B*40/MICA*008/MICB*004	96012	C/T	L1PA3/Tigger3b
88_B*51/MICA*049/MICB*005	94_B*51/MICA*006/MICB*005	100093	G/A	THE1D/ L1M2
32_B*15/MICA*010/MICB*002	28_B*15/MICA*010/MICB*005	101267	A/C	MER2
32_B*15/MICA*010/MICB*002	73_B*15/MICA*010/MICB*006	101267	A/C	MER2
86_B*40/MICA*008/MICB*002	13_B*40/MICA*008/MICB*014	102370	C/T	SVA-MIC indel
21_B*35/MICA*002/MICB*005	1_B*35/MICA*002/MICB*002	105577	G/C	MER21C/MER4
54_B*35/MICA*002/MICB*005	21_B*35/MICA*002/MICB*005	113437	C/G	MER21C/MER4
68_B*35/MICA*016/MICB*002	23_B*35/MICA*16/MICB*005	116829	G/A	5′-ERV3-16A3_I (HCP5)
54_B*35/MICA*002/MICB*005	1_B*35/MICA*002/MICB*002	119556	A/G	5′-ERV3-16A3_I
88_B*51/MICA*049/MICB*005	46_B*51/MICA*009/MICB*002	123534	G/C	5′-ERV3-16A3_I
88_B*51/MICA*049/MICB*005	67_B*51/MICA*009/MICB*005	123570	C/T	5′-ERV3-16A3_I
38_B*40/MICA*027/MICB*005	71_B*40/MICA*027/MICB*013	124806	A/G	THE1A/LTR33
28_B*15/MICA*010/MICB*005	73_B*15/MICA*010/MICB*006	undetected	SPR	SPR HLA-B to MICB gene
19_B*08/MICA*008/MICB*008	27_B*08/MICA*008/MICB*008	no XO	SPR	SPR from HLA-B to MICB
65_B*1401/MICA*019/MICB*502	49_B*1402/MICA*011/MICB*502	SRR/112318/SPR	A/G	5′-ERV3-16A3 (HCP5)
65_B*1401/MICA*019/MICB*502	87_B*14:01/MICA*11/MICB*005:02	SRR/146518/SPR	C/T	L1
38_B*40/MICA*027/MICB*005	86_B*40/MICA*008/MICB*002	undetected	SRR	SRR from HLA-B to MICB
38_B*40/MICA*027/MICB*005	82_B*40/MICA*008/MICB*004	undetected	SRR	SRR from HLA-B to MICB
54_B*35/MICA*002/MICB*005	35_B*35/MICA*017/MICB*003	undetected	SRR	SRR from HLA-B to MICB

*SRR is SNP-rich region, SPR is SNP-poor region, and XO is crossover*.

#### SNP Densities Within MHC Class I Homologous Haplotypes

We grouped and aligned 41 sequences to evaluate the variations of SNP density and the degree of homology within the same CEHs/AHs (Table 4 and Supplementary Table 8). The homologous sequence alignments for 12 of 16 different CEHs/AHs revealed a scarcity of SNPs ranging over ~1.8 Mb from *HLA-F* to *MICB* with <150 SNPs over the entire region at an average of 20 SNPs for 17 sequence alignments. Seven of the 16 different CEHs/AHs were SNP poor (<150 SNPs over ~3 Mb) from the *GPX5* locus in the *OR* gene region to the *MICB* gene in the beta block region. These highly homologous sequence runs represented the seven sequences of *8.1AH*, five sequences of *7.1AH*, two sequences each of *18.2AH, 51.1AH, 57.xAH* and *62.xAH*, and two of three sequences classified as *62.1AH*s. The SNP counts over the same range for the alignment of different haplotypes such as between the *7.1 CEH/AH* and *8.1 CEH/AH* were >2,000 for ~3 Mb.

Six CEHs/AHs had regions of substantial diversity that were SNP-rich between the alpha and beta blocks and/or in the *OR* gene region at the telomeric end of the alpha block. These haplotypes consisted of different-sized, SNP-poor recombinant blocks interspersed between SNP-rich recombinant blocks. The most surprising results were for the comparison between the three sequences of the *62.1 CEH/AH* and the three sequences of the *44.1 CEH/AH*. The sequence of LAB ID_41 had varying regions of SNP density with three SNP XOs, whereas ID_40 and ID_85 had few detectable SNPs (0.77 SNPs per 100 kb) and no XO SNPs in their alignment from *GPX5* to *MICB*. Similarly, the ID_60 sequence with the *44.1 CEH/AH* had at least two SNP XO events, one in the region between *HLA-J* and *HLA-E* and another in the region between *MUC21* and *HLA-C*. In comparison, the ID_74 sequence of *44.1 CEH/AH* had few SNPs and no detectable SNP XO in the 1.8-Mb sequence block from *HLA-F* to *MICB*.

#### Crossing Over Within the *OR* Extended Gene Region

The SNP XOs for some CEHs/AHs were detected in the *OR* gene regions hundreds of kilobases telomeric of the *HLA-F* gene (Table 5). For the sequence comparison between haplotype pairs, the *OR* genomic region was divided into four segmental blocks of 210–300 kb each, ranging from the telomeric *GPX*5 gene to the *ZFP57* gene that are 1,118.4 kb and 42.1 kb telomeric of the *HLA-F* locus, respectively. The average SNP/210 kb for four different haplotype pairs was 317 SNPs within the genomic region between *MAS1L* and the start of the alpha block F segment. In paired sequence comparisons of 23 similar haplotypes, a SNP XO was found in 15 pairs within 237 kb between *MAS1L* and *HLA-F*. A SNP XO was found near to or within *ERV3-16A3*, an ancient *HERV-16* element at the junction of the F segment in five haplotype pairs, within *LTR10A* of two haplotype pairs, within *MER21C* of two haplotype pairs, within *AluY/Sc8* of two haplotype pairs and within *Tigger2b* of one haplotype pair. Most SNP XO were found in loci between *ZFP57* and *HLA-F* (five haplotype pairs), *USB* and *OR2H2* (five haplotype pairs), *GABBR1* and *MOG* (three haplotype pairs) and *OR2H2* and *GABBR1* (two haplotype pairs), revealing the variability of the SNP XO junctions that were involved with ancestral recombinations.

The SNP XO in a region between the *OR12D3* and *ORD12D*2 genes in the sequence alignment of 22_A^*^32-B^*^38 and 72_A^*^32-B^*^44:02 is ~332.2 kb from the *HLA-F* gene. Moreover, this SNP XO site is in close proximity to the young Alu indel, *AluOR*, that was detected in Japanese and Caucasians at a frequency of 0.32 and 0.14, respectively (Table 1). This SNP XO and active Alu insertion site appear to mark a hotspot for meiotic and insertion recombinations. Of the seven other sequence comparisons, no SNP XO was detected in two pairs (ID4 v ID6 and ID78 v ID79) of sequences that ended at the *MA1L/LINC01015* segmental block and in five pairs (ID11 v ID16, ID4 v ID51, ID34 v ID94, ID25 v ID26, and ID46 v ID82) that ended near the *GPX5* locus because of the absence of sequence for further analysis. The comparisons between two *7.1AH* (ID4 v ID51) and two *8.1AH* (ID11 v ID16) were striking because the SNP-poor region extended from the alpha block to at least the *GPX5* gene that is ~1118 kb from the *HLA-F* gene. Both of the extended A^*^30-B^*^18 haplotypes (ID25 v ID26) carried the haplospecific *AluOR* insertion and the novel *AluOR1* insertion (Table 1).

#### SNP Density XOs Within the Alpha Block of Different *HLA-A* Haplotypes

The alpha block was divided into 10 segments containing the duplicated HLA genes and pseudogenes from segment F with the *HLA-F* gene to segment J with the *HLA-J* pseudogene (Dawkins et al., 1999; Kulski et al., 1999a,b) for SNP counts and XO analysis (Table 6) as shown in Supplementary Figure 6 and Supplementary Table 3. The SNP counts were zero to less than 20 SNPs over 320.3 kb of sequence for 20 of 35 similar alpha block haplotype pairs (Table 6). In contrast, the SNP counts were much greater in the sequence alignments of seven different haplotype pairs ranging between 2,035 and 2,644 SNPs per 320.3 kb at an average of 7 SNPs/kb. The highest average SNP density was 16 SNPs/kb in the A segment and the lowest density was 2 SNPs/kb in the J segment. Because of deletions or XO within the alpha block, some recombinant haplotypes had intermediate SNP numbers such as 692 to 884 SNPs per 320.8 kb for six of the HLA-A^*^24 haplotypes, 269 SNPs for the 48_A^*^24 vs. 59_A^*^24 haplotypes and 707 SNPs for the 46_A^*^31 vs. 49_A^*^33 haplotypes. The smallest amount of SNP diversity within the alpha blocks of different *HLA-A* allelic lineages was between the HLA-A^*^26 and the HLA-A^*^66 haplotypes with only 66 SNP differences across the 320.8 kb alpha block (one SNP per 4.9 kb compared to an average of one SNP per 0.14 kb for an average of seven haplotype pairs). The biggest SNP difference among the different haplotypes was in the 53-kb G-segment and the smallest was within the 15-kb A segment that had only a total of three SNP differences. A previous analysis of the HLA-A^*^26/A^*^66 loci indicated that the HLA-A^*^66 was a product of a gene conversion (Madrigal et al., 1993). Alternatively, the relatively small number of SNP differences across the entire alpha block of the HLA-A^*^26/A^*^66 haplotypes suggests that they might have evolved from the same AH and diverged slightly by gene conversions and mutagenesis over time because of their age.

An intermediate amount of SNP diversity was detected between HLA-A^*^31 and HLA-A^*^33 with 707 SNPs within the 320.8-kb alpha block. However, the first 100 kb of the alpha block including the F, V and P duplicated segments was 605 SNPs and the remaining 220 kb of the alpha block from the G segment to the J segment was only 102 SNPs including 11 SNPs in the A segment. This segmental division with sequence homogeneity at the centromeric end of the HLA-A^*^31 and HLA-A^*^33 haplotype sequences and large diversity at the telomeric end of their alpha blocks has an ancestral SNP XO at the centromeric end of the P segment within the *MICG* pseudogene (C/T). In comparison to the 707 SNPs across the alpha block of the HLA-A^*^31 and -A^*^33 haplotypes, there were 2,287 SNPs across the alpha block of the HLA-A^*^03 and -A^*^33 haplotypes. Coincidently, the HLA-A^*^03, -A^*^31, and -A^*^33 haplotypes all have an *HERVK9* insertion. Thus, two thirds of the HLA-A^*^31/^*^33 alpha blocks have the same haplotype lineage whereas the other third are evidently from different haplotype lineages.

The largest difference among different *HLA-A* haplotypes was between HLA-A^*^30 and HLA-A^*^31 with 2,644 SNPs in the alpha block, whereas on average there were 2,235 SNPs in the alpha block for seven different sequence pairs. The biggest differences between the same *HLA-A* haplotypes were obtained for the HLA-A^*^24 pairs, suggesting that their alpha blocks may have undergone numerous shuffling and exchanges with various other *HLA-A* haplotypes. No SNPs were detected in the alpha blocks of the HLA-A^*^32 haplotype pair and only one SNP was detected in the alpha blocks of an HLA-A^*^24 haplotype pair and the HLA-A^*^29, -A^*^30, and -A^*^31 haplotype pairs.

**Figure 2:**
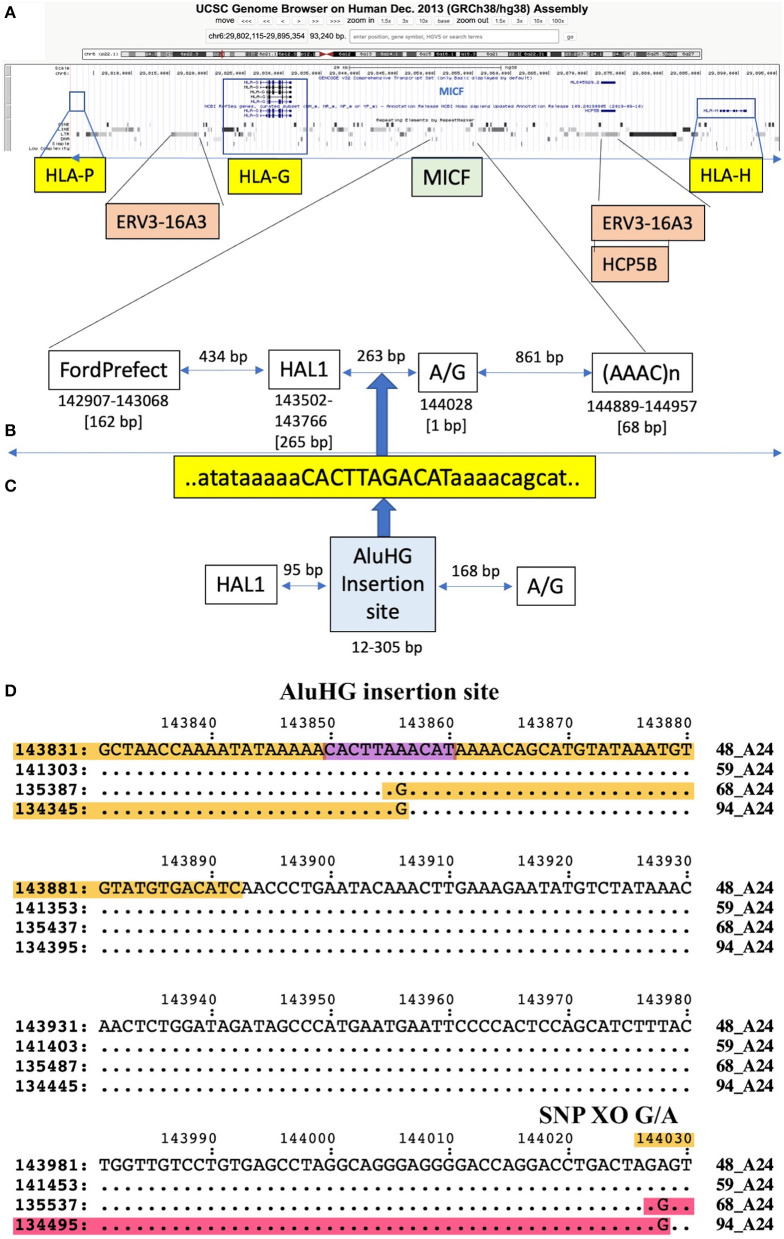
Putative recombination site in the *MICF* pseudogene region with a SNP rich to SNP poor crossover in segment G at 144028 *G/A*, which is 168 bp from the site of an *AluHG* insertion site in the sequence alignment of ID48_A24, ID59_A24, ID68_A24, and ID94_A24 (Table 6). **(A)** Location of the *MICF* pseudogene in segment G relative to the *HLA-P, HLA-G*, and *HLA-H* genes as depicted in the UCSC browser. **(B)** Magnification of the *MICF* pseudogene region showing the positions of the flanking TE, *FordPrefect* and *HAL1*, and the *A/G* SNP crossover located between *HAL1* and the (*AAAC*)n microsatellite. **(C)** The *AluHG* insertion site and sequence *CACTTAGACAT* located between *HAL1* and the *A/G* SNP position. **(D)** A 200-bp nucleotide sequence showing the location of the *AluHG* insertion site and the SNP *G/A* crossover (XO) site in a comparison between four different haplotypes (48, 59, 68, and 94) with the same HLA-A*24 allelic lineage. The structural biallelic *AluHG* insertion is linked to HLA-A*02, but is absent in HLA-A*24 (Table 2). The *AluHG* TD insertion site *CACTTAAACAT* (Kulski et al., 2001) is outlined in purple.

The SNP XOs for the same haplotypes at the telomeric end of the alpha block were variable depending on which haplotypes were compared (Table 6), but mostly involved the F segment with the *HLA-F* gene and sites within or between the *Tigger1* and *Charlie20* DNA elements. In genomic sequence comparisons between seven similar HLA-A^*^24 haplotype pairs, all of them had the 54-kb deletion of the T and K segments between the H and A segments (Supplementary Figure 5). Also, some XOs occurred in the G segment at A/G 141380 between *HAL1* and *(ATAAT)*n, which is near the *AluHG* insertion locus and the *MICF* pseudogene (ID34 v ID48, ID34 v ID59, ID48 v ID68, and ID48 v ID94) (Figure 2).

The sequence comparison between the HLA-A^*^02:01:01 and HLA-A^*^02:05:01 haplotypes revealed large SNP differences within five segments from F to H of the alpha block (ID5 v ID8 and ID10 v ID8). This difference indicates that an exchange of segments T, K and A had occurred in the HLA-A^*^02:05:01 haplotype (Italian cell line WT49) that lacks the haplospecific A^*^02 lineage marker *AluHG* within the G segment (Supplementary Table 2). The sequence comparison between 4_A^*^03:01:01:01 and 18_A^*^03:01:01:01/A^*^24:02:01 with a low SNP density within the segments H to A is noteworthy because it reveals that the 18_A^*^03:01:01:01/A^*^24, if assembled correctly from the heterozygous Australian Caucasoid cell line LO081785, is an atypical and highly divergent A^*^03 haplotype with a *HERVK9* deletion (Hap ID19.A^*^03.2 in Supplementary Table 6 and ID_18 in Supplementary Table 2). The SNP XO was within the G segment at nucleotide position 150337 C/T located between the *Tigger1* and *Charlie20a* DNA elements (Table 6).

#### XO in Regions Between *HLA-J* and *HLA-C*

**(a) SNP XOs within *HLA-A* haplotypes**. The analysis of XO junctions in the genomic sequences between *HLA-J* and *HLA-C* outside the alpha and beta blocks using the same *HLA-A* and different *HLA-C* alleles was limited to only 15 comparisons (Supplementary Table 9). Of these, the XO was between *HLA-A* and *HLA-J* in two comparisons, between *HLA-J* and *HLA-E* in six pairs, and between *HLA-E* and *MUC21* in six pairs. In the sequence comparison between 11_A^*^01-C^*^07-B^*^08 and 2_A^*^01-C^*^06-B^*^57 (example 6^*^ in Supplementary Table 9), at least five different XO junctions were detected in different regions of segments B to E, including the transition from SPR to SRR in block B, two separate XO transitions in block C, a SRR to SPR XO in block D and a SPR to SRR XO in block E. Multiple XOs were observed also for analyses numbered 5 to 8 in Supplementary Table 9. The entire SNP-poor region (20 SNPs) was extended from *HLA-J* to *HLA-C* in the sequence alignment of cell lines with the same haplotype, A^*^01-C^*^06.

**(b) SNP XOs within *HLA-C* haplotypes**. Supplementary Table 10 shows the XO regions for 44 recombinant sequence pairs with the same *HLA-C*, but different *HLA-A* allelic lineages. A control homologous sequence pair (analysis 45) with the same *HLA-A* and *HLA-C* allelic lineages, 2_A^*^01-C^*^06-B^*^57 and 31_A^*^01-C^*^06-B^*^40, was SNP poor with only 20 SNPs counted over 1,322.9 kb of sequence. In comparison, the other sequences were heterologous (SNP rich) over most of the genomic region between the *HLA-A* and *HLA-C* genes. XOs occurred from SNP rich to SNP poor within the *HLA-C* gene or the 3′ non-coding region (NCR) of *HLA-C* in a comparison of 14 haplotype pairs, implying that recombinations occurred within the coding region of the ancestral HLA-C^*^07:18 and some other ancestral HLA-C^*^07 allelic lineages. The *HLA-C* haplotypic alleles that transitioned from SNP-poor to SNP-rich regions within 12 kb of the 3′end of exon 8 of *HLA-C* included C^*^01, C^*^04, C^*^05, C^*^07, C^*^08, C^*^12, and C^*^14 depending on their linkage with *HLA-A* alleles. The C^*^03/HLA-A^*^01, -A^*^02, -A^*^24, and the C^*^17/HLA-A^*^01, -A^*^30 combinations were SNP poor until the *PSORS1C3* gene that is located about 100 kb from the *HLA-C* gene. Some of the HLA-C^*^06 and HLA-C^*^07 alleles within different recombinant haplotypes (analysis numbers 31 to 34) were homologous (SNP-poor) in the region from the *HLA-C* locus to the *HCG22* locus. This long-range, homologous genomic segment of ~215 kb between *HLA-C* and *HCG22* includes the various psoriasis candidate genes *HCG27, PSORS1C3, POU5F1, TCF19, CCHCR1, PSORS1C2, PSORS1C1, CDSN*, and *C6orf15* (Nair et al., 2006). However, in combinatorial analyses of recombinant risk haplotypes and alleles genotyped in 678 families with early-onset psoriasis, Nair et al. (2006) determined that HLA-C^*^06 was the solitary risk allele that conferred susceptibility to early-onset psoriasis. Other *HLA-C/HLA-A* recombinant haplotypes were SNP poor over even greater distances ranging between 156 kb from the *HLA-C* gene to C6orf15 (C^*^01) and 730 kb from the *HLA-C* gene to a region between the *LINC02569* and *GNL1* loci. XO between SPR and flanking SRRs also occurred within or near to the *MUC22* and *MUC21* genes that are 234 kb and 279 kb from the *HLA-C* gene locus, respectively.

#### SNP-density XOs in Regions Between Different *HLA-C* and *HLA-B* Alleles

**Figure 3:**
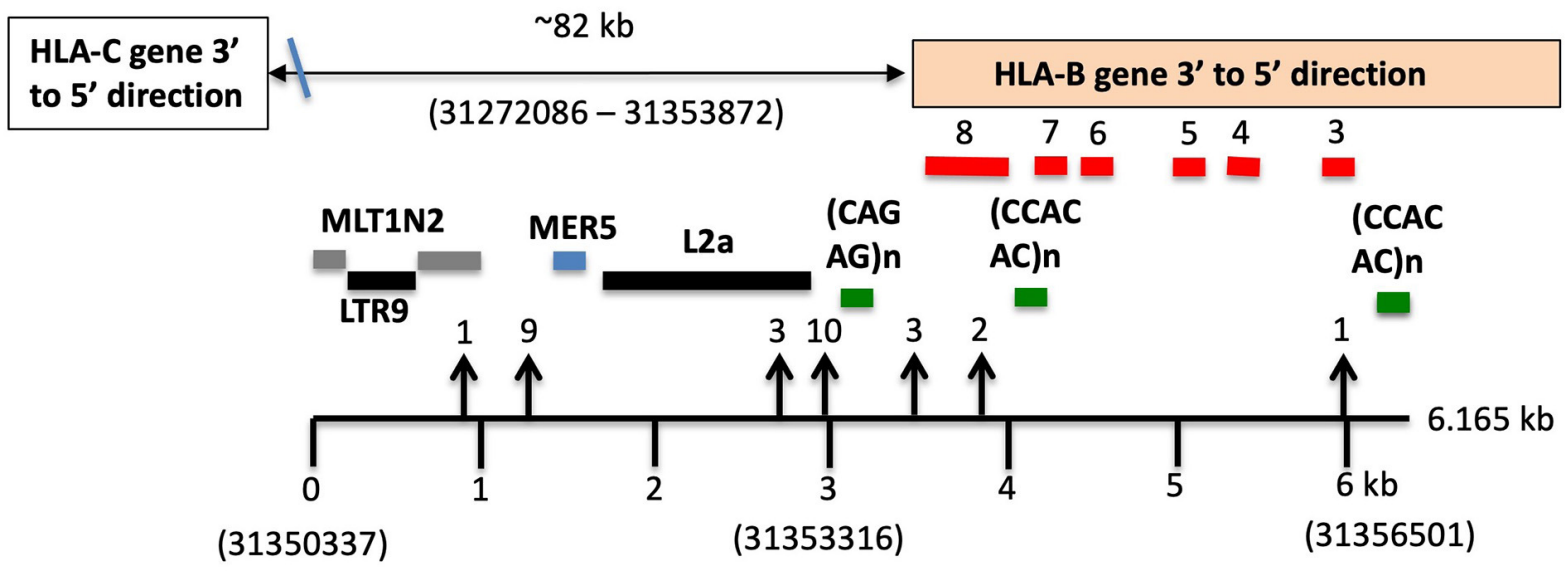
SNP crossover sites within a 3-kb *HLA-B* extended 3′non-coding region (3′-NCR) of a 6-kb genomic region indicated by the lower horizontal line. Repetitive elements (grey, blue, and black horizontal lines) and STRs (green horizontal lines) are labelled. Numbers in brackets are the Reference Genome positions on chromosome 6 (UCSC = GRCh38/hg38 assembly—https://genome.ucsc.edu). The location of the *HLA-B* gene is boxed and the relative exons labelled 3 to 8 are indicated above the red bars. The *HLA-C* gene is ~82 kb telomeric of the *HLA-B* gene. SNP crossover loci are indicated by vertical arrows and were found within 29 of the 51 *HLA-C/HLA-B* haplotype alignments shown in **(C,D)** of Table 7. Numbers above the arrowheads represent the number of different haplotype alignments revealing the SNP crossovers. Although more than half of the crossover sites were found within the 3-kb 3′-NCR of *HLA-B*, six were found within HLA-C and another 13 were outside the 3-kb 3′-NCR of *HLA-B* and downstream towards *HLA-C*.

Table 7 shows the results of SNP counts and XO loci for 59 *HLA-C* and *HLA-B* haplotype sequence alignments using various combinations of similar and different haplotypes. As controls for comparing the various *HLA-C/HLA-B* recombinants, two were different *HLA-C* and *HLA-B* haplotypes and six were the same *HLA-C* and *HLA-B* haplotypes. The different haplotype pairs yielded an average of 1,656 SNP counts for ~93 kb, whereas the same haplotype pairs produced a few or no SNPs over the same distance between *HLA-C* and *HLA-B* loci. There were 39 sequence pairs of *HLA-C/HLA-B* recombinants with the same *HLA-C* allele and a different *HLA-B* allele, whereas 12 pairs of *HLA-C/HLA-B* recombinants had the same *HLA-B* allele and different *HLA-C* allele. Most SNP XO occurred near to or within the *HLA-B* or *HLA-C* coding region depending on which *HLA-C/HLA-B* recombinant haplotype sequences were aligned. SNP XOs occurred within the 3′ NCR or coding region of the *HLA-B* gene and within a 3-kb portion between a *MLT1N2* element and the *HLA-B* gene (Figure 3) for 28 of 51 *HLA-C/HLA-B* haplotype alignments (Table 7). The SNP XOs in the intermediate loci regions were (1) within the *L1PA13* fragment that is ~9 kb from *HLA-C* and ~72 kb from *HLA-B*, (2) between the *L1MB8* and *MLT1D* elements ~53 kb from *HLA-B*, (3) a *MIR* element ~44 kb from *HLA-B*, and (4) within the *HERVI* ~21.5 kb from the *HLA-B* gene.

#### SNP-density XOs in Regions Between Various *HLA-B* and *MIC* Gene Alleles

**Figure 4:**
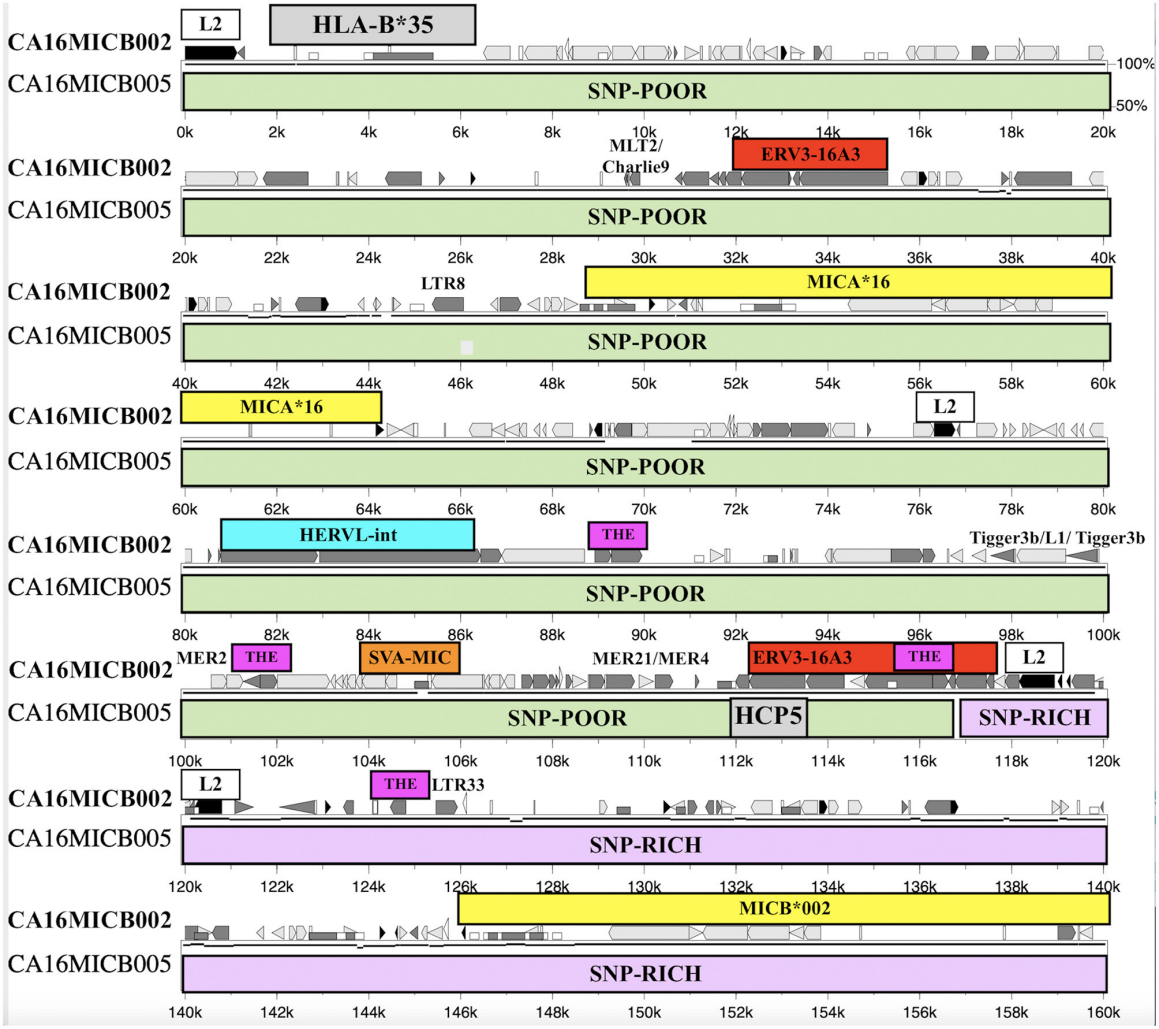
Percent Identity Plot and SNP crossover site within the *ERV3-16A3* sequence and 3′ of the *HCP5* long coding RNA locus of sequence alignment from *HLA-B* (grey box) to *MICB* (lowest yellow box) between the haplotypes ID68_B*35-MICA*016-MICB*002 (upper) and ID23_B*35-MICA*16-MICB*005 (lower). The SNP-poor region (green rows) and SNP-rich region (lilac rows) are indicated below the upper sequence that also has the labelled locations of HLA-B*35, MICA*16, MICB*002, and repeat elements *L2, ERV3-16A3, HERVL, SVA-MIC, MLT2/Charlie9, LTR8, Tigger3b/L1/Tigger3b, MER2, MER21/MER4, THE1*, and *LTR33*. The other repeats in the upper sequence such as the *Alu, MIR*, and *L1* fragments are unlabelled but are indicated with the symbols used by Schwartz et al. (2000).

The SNP XOs in the genomic region between *HLA-B* and *MICA* and between *MICA* and *MICB* for 31 recombinant haplotypes are listed in Table 8. There are 12 examples of a SNP XO within the genomic region of ~46.4 kb between *HLA-B* and *MICA*, and 13 examples of a SNP XO in the 94.5 kb genomic region between *MICA* and *MICB*. A SNP XO was detected in the *MICA* gene for the comparison between 54_B^*^35/MICA^*^002/MICB^*^005 and 68_B^*^35/MICA^*^016/MICB^*^002. The SNP XOs in the genomic region between *MICA* and *MICB* were within or near to putative recombinant hotspot REs: *Tigger3b, MER2, THE1, MER21, LTR33*, and *ERV3-16A3_I* of the *HCP5 lncRNA* gene (Figure 4). A SNP XO was found also within the insertion sequence of the *SVA-MIC* indel (Table 1) for the haplotypes 86_B^*^40/MICA^*^008/MICB^*^002 and 13_B^*^40/MICA^*^008/ MICB^*^014. SNP XO locations were not identified in five sequence comparisons because the genomic region from *HLA-B* to *MICB* was either too SNP-rich (comparisons 29 to 31) or too SNP-poor (comparisons 25 and 26).

## Discussion

The comparative sequence analyses of the 95 genomic sequences using RepeatMasker (Hubley et al., 2016) and PIP (Schwartz et al., 2000) confirmed the identity and HLA class I allelic linkages of 12 haplotypic RE markers (Table 1) that had been genotyped previously in population frequency studies and MHC homozygous and heterozygous cell lines (Kulski and Dunn, 2005; Kulski et al., 2011). We identified another four novel REs and indels; three of them were annotated as *AluOR1, AluP5*, and *SVA-BC* indels (Table 1, Figure 1). A fourth indel was 9.5 kb in size and composed of the *MER5B* and *LTR33* subfamily members (Supplementary Figure 1). The deletion variant of the 9.5-kb indel that is located between the *SVA-BC* and *SVA-HB* loci within the beta block was linked to all 11 HLA-C^*^06:02 alleles, all 4 HLA-C^*^12:03 alleles, and 1 of 9 C^*^04:01 alleles (Table 3). The 9.5-kb indel, *SVA-BC* and *SVA-HB* are all located within a relatively strong recombination hotspot of 82 kb between the *HLA-B* and *HLA-C* genes (Figures 1, 3). This intergenic recombination hotspot was identified previously in sperm studies (Cullen et al., 2002) and HapMap studies (Lam et al., 2013). Interestingly, a SNP XO was detected within the *SVA-MIC* insertion between the *MICA* and *MICB* loci (Figure 4) of the A^*^02/C^*^03:04/B^*^40 haplotype pair ID13 and ID86 (Table 3), suggesting that this insertion locus is near a recombination site that exchanged the MICB^*^002:01 allele with MICB^*^014.

We included eight HLA class I pseudogenes (*V, P, H, T, K, U, W*, and *J*) as haplotypic markers in our analysis of the alpha block haplotype diversity even though most of them may have no physiological functions or regulatory roles. However, the *HLA-H* gene is polymorphic and has transcriptional activity, and the signal peptide of the membrane-bound HLA-H molecule can mobilise HLA-E to the cell surface of mononuclear cells, bronchial epithelial cells and lymphoblastoid cell lines (Jordier et al., 2020). The HLA-H^*^02:07 allele was found at a frequency of 19.6% in some East Asian populations (Paganini et al., 2019) and encodes a full-length HLA protein that may have tolerogenic activity (Jordier et al., 2020) in comparison to immunosuppressive activity of its neighbouring gene product, HLA-G (Elliott, 2016). Taken together, the alleles of the pseudogenes confirmed that the alpha block haplotypes consist of multilocus alleles and that the recombinant XOs were mostly at the telomeric and centromeric ends of the block often within the telomeric F segment and/or the centromeric W and J segments (Table 2), but also in *MICF* fragment of the G segment close to the biallelic *AluHG* insertion site (Figure 2) and in proximity of *HERVK9* of the *HLA-H* segment associated with the 54-kb deletion of the HLA-A^*^24 lineage (Supplementary Figure 5). Thus, the haplotypic *HERVK9, AluHF, AluHG*, and *AluHJ* insertion loci are located within 2 to 15 kb of these SNP XO junctions that might have co-evolved as a consequence of recombination events between AH. In previous population and homologous cell line genotyping studies, the *AluHF* insertion was associated with HLA-A^*^26, AluHJ was associated with HLA-A^*^01, -A^*^24 and -A^*^32 (Dunn et al., 2002; Kulski et al., 2019) and *AluHG* was associated with HLA-A^*^02 (Kulski et al., 2001) and HLA-G^*^01:01 (Santos et al., 2013) allelic lineages. The present study not only confirms these Alu haplospecificities and HLA associations but also has linked them to HLA pseudogene alleles and the non-classical *HLA-F* and *HLA-G* alleles (Table 2). Thus, the *AluHF* insertion, which is 12.4 kb telomeric of the *HLA-F* genes and within an *ERV3-16A3_int* sequence, is linked to HLA-F^*^01:01:01:08 and a number of different *HLA-A* alleles (A^*^02, A^*^03, A^*^26, A^*^29, and A^*^67) revealing the occurrence of frequent recombination events in the proximity of the *HLA-F* gene. The *AluHG* insertion is linked consistently to the HLA-G^*^01:01/HLA-H^*^01:01 haplotypic alleles and mostly to HLA-A^*^02. However, the occasional linkage of the AluHG/HLA-G^*^01:01/HLA-H^*^01:01 haplotype with HLA-A^*^30 and HLA-A^*^31 suggests a recombination site within a region somewhere nearer the *HLA-A* locus and probably within the A segment. Similarly, the *AluHJ* insertion at the centromeric terminal end of the alpha block is linked mainly to HLA-A^*^01/HLA-J^*^01:01:01:02, HLA-A^*^24/HLA-J^*^01:01:01:02 and HLA-A^*^33/HLA-J^*^01:01:01:06 of the 25 analysed haplotype sequences. The two exceptions were the HLA-J^*^01:01:01:02/AluHJ linkage in the heterologous A^*^03/A^*^24 sample and the linkage of HLA-J^*^01:01:01:02/AluHJ to the HLA-A^*^02 that again provides different examples of recombination activity within and at the borders of the alpha block.

The alpha block *HERVK9* insertion in chimpanzee (Kulski et al., 2005), gorilla (Wilming et al., 2013) and human (Kulski et al., 2008) heralds its ancient hominid lineage. The loss and replacement of the *HERVK9* sequence with a solitary *MER9* sequence presumably generated the most recent alpha block haplotypes. In contrast, the absence of the *SVA-HB* from the beta block is the ancestral lineage for the *SVA-HB* indel (Kulski et al., 2010). The ancient *HERVK9* insertion allele occurs frequently in the Caucasian, African American and Japanese ranging between 34 and 59% (Kulski et al., 2008) as does the *SVA-HB* insertion that was genotyped at 65% in Caucasians, 64% in African Americans and 25% in Japanese (Kulski et al., 2010). Both the alpha block *HERVK9* insertion and the beta block *SVA-HB* insertion separate the *HLA-A/B/C* haplotypes into four distinct ancestral lineages, *HERVK9*+*/SVA-B*+, *HERVK9*+*/SVA-B-, HERVK9-/SVA-B*+ and *HERVK9-/SVA-HB-*. Other TE alleles and indels could be added to the TE classification system as more data evolve to assess MHC class I segmental exchanges and recombinants (Kulski et al., 2011), but this would require more annotated data on polymorphic TE and indels than presently available. Also, the question arises whether the nine different alpha-beta block haplotypes (Tables 2, 3) with the *HERVK9* insertion and no *SVA-HB* insertion that includes A^*^03:01/C^*^07:02/B^*^07:02 are older than those with the alpha block *HERVK9* deletion and the beta block *SVA-HB* insertion (28 different haplotypes) (Supplementary Table 5). Although the *7.1AH* with the *HERVK9*+*/SVA-B-* alleles might be older than *8.1AH, 27.1AH, 57.1AH, 60.1AH*, and *62.1AH* that are *HERVK9-/SVA-HB-*, classifying the evolutionary age of the haplotypes according to the presence and absence of *HERVK9* and *SVA-HB* is probably unreliable because of segmental conversions and exchanges between genomic sequences. In addition, the *7.1AH* and the *8.1AH* are among the most common European haplotypes with population frequencies of *8.1AH* up to at least 18% (Sanchez-Velasco et al., 2003; Kiszel et al., 2007; Sanchez-Mazas et al., 2013; Robinson et al., 2019). In addition, seven of the other nine *HERVK9*+*/SVA-B-* haplotypes (Supplementary Table 5) are common in Europeans (Suslova et al., 2012; Robinson et al., 2019) or Japanese (Ikeda et al., 2015), whereas A^*^31/C01:02/B^*^15:01 is from the cell line of a Warao South American Indian, which may be moderately common in some Amerindians (Watkins et al., 1992; Cadavid and Watkins, 1997), but not in others (Barquera et al., 2020). Taken together, these observations suggest that the human MHC *ERVK9*^+^*/SVA-HB*^−^ haplotype might be broadly spread at relatively high frequency within many different worldwide populations.

The most commonly inferred haplotype blocks that are free of genetic recombination are supposedly those that are identified by LD statistical tests of SNP associations (Daly et al., 2001; Ahmad et al., 2003; Miretti et al., 2005; Blomhoff et al., 2006, Traherne, 2008). However, SNP densities vary markedly throughout the human genome (Sachidanandam et al., 2001) and their transition between SNP-rich and SNP-poor regions can be used to identify recombination free haplotypes without the need for LD tests (Myers, 2005; Kauppi et al., 2007; Bairagya et al., 2008). In the present study, we identified the junctions of haplotype blocks on the basis of SNP densities using homozygous haplotype sequences without applying LD statistical tests. We classified the haplotype junctions as XOs between SNP poor and SNP rich regions of haploidentical and haplodiverse genomic sequence comparisons (Tables 5–8). The SNP XOs between rich and poor SNP regions revealed a clear block structure both for haplotype mapping potential ancestral recombination sites and for objective analysis of haplotypes in different populations for disease associations. Since block patterns vary between common haplotypes, it will be important to construct maps for each different haplotype from a variety of populations to assess the degree of haplotype diversity within and between populations (Goodin et al., 2018). Many of the XOs that we identified are in close vicinity of recombination hotspots (Figure 1) that were previously identified in studies of MHC recombination sites using homozygous sperm DNA (Cullen et al., 2002) and HapMap populations (Lam et al., 2013).

SNP densities in our haplotype alignments were counted manually while avoiding obvious assembly errors, polynucleotides, simple microsatellite repeats, and indels by scanning overlapping windows of 50 to 500 kb of sequence. SNP-poor regions were easy to count manually because of small SNP numbers (<5 SNPs/100 kb), but SNP-rich regions were more difficult because of large SNP numbers at an average of 7 SNPs/kb. In comparison to our findings, Lam et al. (2015) reported an average of 7 SNPs/100 kb for the *A33-B58-DR3* haplotype for 4136 kb and 8.7 SNPs/100 kb for the *A2-B46-DR9* for 2721 kb, which are much higher than our comparative counts. This difference is possibly due to spikes of diversity in their localised regions of sequence. The SNP counts by Lam et al. (2015) within homologous haplotypes represented as nucleotide diversity values were still at least 38× less than that found between *7.1CEH/AH* and *8.1CEH/AH*, the two different common European MHC heterologous haplotypes of the cell lines PGF and COX, respectively. The count of 2240 SNPs per 320.8 kb (7 SNPs/kb) for PGF (LAB ID_4) and COX (LAB ID_11) in our study when their alpha blocks were aligned (Table 6) further demonstrates the large SNP diversity between them. In comparison, the average SNP density of the human genome is one SNP per 1.9 kb or between 5 and 9 SNPs per 10 kb (Sachidanandam et al., 2001; Zhao et al., 2003).

The two longest MHC haplotypes in the human genome are considered to be the European *7.1AH* and *8.1AH* (Horton et al., 2004, 2008) with CPS extending into the telomeric *OR* gene cluster region beyond the *GPX5* gene that is 1.2 Mb telomeric of *HLA-F* (Figure 1). However, the CPS of at least four other MHC class I haplotype pairs also extended beyond the *GPX5* gene: *18.2AH, 51.xAH, 57.1AH, 62.xAH*, and *62.1AH* (Table 5, Supplementary Table 8). The CPS of another three *HLA-A* haplotype pairs also extend beyond the *GPX5* gene: HLA-A^*^24/C^*^03:04, HLA-A^*^31/C^*^15:02 and HLA-A^*^32/C^*^12:03 presented in Supplementary Table 8. The SNP XO junctions could not be determined for these haplotypes because they are telomeric of the *GPX5* gene (NCBI ID:2880) and located somewhere beyond the region of the available genomic sequences provided for these cell lines (Norman et al., 2017). In comparison, the SNP XO junctions for two other HLA-A^*^01/C^*^06 and HLA-A^*^01/C^*^07 pairs and two HLA-A^*^03/C^*^07:02 and HLA-A^*^03/C^*^06:02 pairs were closer to the *MOG* and *OR2H2* genes (Table 5) that are 51 kb and 134 kb from the *HLA-F* gene, respectively (Figure 1). It seems that the recombinant breakpoint of different haplotype segments or blocks generates the SNP XO site even if the classical HLA alleles are the same. For example, a number of HLA-A^*^02 allelic lineages were represented by distinctly different alpha block haplotypes (Table 2) that evolved from various shuffling events as well as segmental conversions (Table 6). Thus, the SNP XO within the alpha block F segment was strongly correlated with different local *HLA-F* alleles even if the downstream *HLA-A* allele within the A segment of the alpha block was the same; that is, the XO between HLA-F^*^01:01:01:01 of *18.xAH* or *38.a2AH* and F^*^01:04:01:02 of *60.1AH* are both linked to HLA-A^*^02. The SNP XO defines the junction of the haplotype XO, which in turn points towards a putative ancestral recombination site. Whether this relationship that is based on a small number of comparative examples can be confirmed in the future will depend on the empirical findings of a much greater number of haplotypes with different *HLA-A* allelic and haplotypic lineages.

During mammalian and primate evolution, the MHC region transitioned through various genomic rearrangements including recombinant XO events of *MIC, HERV16* and *HLA* class I genes, which resulted in the current structural organisation of the human MHC class I genomic region (Kulski et al., 1999a,b; Kulski et al., 2004). The *HERV16*, now classified as *ERV3-16A3* (repeated throughout the MHC class I and class II regions), along with HLA class I coding and non-coding sequences, seems to have been a recombination site for many of the duplication events by way of unequal XOs (Kulski et al., 1999a,b; Kulski et al., 2004). Ancient hominoid haplotypes undoubtedly were the progenitors to the modern human CEH/AH, but when, how and where is unknown. In addition, each AH is a unique integrated genetic module consisting of many immunologically related protein-coding genes with gene copy number variations, segmental duplications and fragmented or relatively intact transposons and REs that contribute to more than 50% of the genomic content. In this regard, the MHC haplotype comprising a cluster of multilocus, monocistronic expression units is analogous to the polycistronic bacterial operon, which is a functional unit of DNA containing a cluster of genes under the control of a single promoter (Lee and Sonnhammer, 2003; Blumenthal, 2004). However, the MHC haplotype structures are far more complex with their regulatory network of cis-acting multilocus expression units known as haplotype-specific expression quantitative trait loci (eQTL) (Lamontagne et al., 2016; Lam et al., 2017) that are largely controlled by an array of virus-derived REs and DNA transposons, both as binding sites for transcription factors and as sources of regulatory non-coding RNAs (Kulski, 2019; Sznarkowska et al., 2020). Random mutations, methylations and recombinations can generate considerable haplotype diversity that is part of the MHC immune system's response to highly prevalent infectious (Gao et al., 2019; Sanchez-Mazas, 2020) and chemical agents, including those responsible for drug hypersensitivities (Alfirevic and Pirmohamed, 2010).

Various factors have been hypothesised to elucidate the generation and maintenance of MHC CEHs/AHs including recombination suppression, balancing selection and demographic factors such as population bottlenecks, genetic drift, migration and admixture (Aly et al., 2006; van Oosterhout, 2009; Prohaska et al., 2019). If CEHs/AHs were generated in recent population history, for example, during the last 21,000 to 26,000 years as estimated from the mutation rates of the Caucasian *A1-B8-DR3-DQ2* haplotype (Smith et al., 2006) and the Asian *A2-B46-DR9* and *A33-B58-DR3* haplotypes (Lam et al., 2013), then there may have been insufficient time over a period of a few thousand generations to have disrupted the LRHs by recombination. It seems that there is a time-associated equilibrium between the population amplification of the LRH and its meiotic recombinational breakage over periods of human population coalescent times (Song et al., 2017; Wang et al., 2020). Polymorphism and sequence heterozygosity also might suppress crossing over and recombination (Ohta, 1999; Dluzewska et al., 2018) and it is well-known that an excess of heterozygosity (overdominance) contributes to MHC diversity as one form of balancing selection (van Oosterhout, 2009; Lenz et al., 2016; Lobkovsky et al., 2019). However, in the context of molecular mechanisms and our study, the connection between the silencing of TE/REs and recombination suppression warrants greater consideration (Campos-Sánchez et al., 2016). TEs are known to affect recombination rates by acting as recombination modifiers, activators and suppressors in mice and humans (McVean, 2010; Altemose et al., 2017; Yamada et al., 2017). Although TEs can directly modulate the local recombination environment either through silencing-mediated suppression or the recruitment of recombination hotspots, the silencing of TEs and other repetitive sequences may also contribute directly to recombination suppression. The densities of DNA methylation and repressive chromatin marks associated with the silencing of TEs and other repeats are often negatively correlated with recombination rates (Myers, 2005; Kent et al., 2017). A recombination suppression mechanism discovered and studied in recent years involves the PRDM9-mediated recombination machinery that initiates at specific sequence motifs and alters chromatin structure (Myers et al., 2010; Parvanov et al., 2017). In humans, PRDM9 determines the locations of meiotic recombination hotspots and binds multiple motifs including the *ATCCATG/CATGGAT* motif of the *THE1B* repeat for both dependent and independent recombination suppression (Myers et al., 2008; Altemose et al., 2017). The active PRDM9 binding sites are also enriched with other classes of human repeat sequences including *L2* LINEs and *AluY* elements (Myers et al., 2008; McVean, 2010). Recent studies with B6 mice demonstrated that a third of meiotic DNA strand breaks occurred within repetitive sequences of different classes, especially within the DNA transposons like *TcMar-Mariner* and *hAT-Charlie*, that resulted in their depletion from the PRDM9 binding sites (Yamada et al., 2017). This finding led the authors to hypothesise that the PRDM9 coevolved with meiotic recombination in order to target active transposons and limit their spread by inactivating or eliminating them by creating mutations or deletions at the PRDM9 binding site. Moreover, a proportion of the duplicated MHC *ERV3-16A3_I (HERV16 int*) sequences (Kulski and Dawkins, 1999; Kulski et al., 1999a,b) have the PRDM9 binding motif *ATCCATG/CATGGAT* at one or two of its nucleotide positions (Supplementary Figure 7).

Although TEs, methylations and recombinations can influence each other markedly (Myers et al., 2008; McVean, 2010; Moolhuijzen et al., 2010; Jones, 2012; Zamudio et al., 2015; Altemose et al., 2017; Kent et al., 2017), there is a surprising paucity of such studies in the MHC genomic region of different haplotypes (Rakyan et al., 2004; Jongsma et al., 2019). The genomic PRDM9 binding motif *ATCCATG/CATGGAT* (Altemose et al., 2017) is spread broadly throughout the MHC class I region and can be found in various fragmented TEs: *L1, L2, MER5, HUERS-P3-int* (2×), *HERVK9-int* (2×), *HERVK14* (2×), *LTR73, MER2, MER8, MER41, MER84, Charlie9, MLT1, MLT2B, Tigger1, ERV3-16A3_I, four* of ~1450 *Alu* elements and various coding regions including the *MICB* sequence (present study, data not shown). The 38 SNP XOs at the borders of the haplotype blocks that are described in our study appear to reflect regions of historical recombination sites that currently might be involved with recombination suppression and haplotype maintenance (Figure 1). Some TEs are likely to have provided the recombination sequence motifs that Cullen et al. (2002) considered could be due to microsatellites and particularly to those with long tracts of *GT* repeats. Many of the SNP XO junctions are within 10 kb of TEs that are commonly repeated throughout the MHC genomic region including *LTR16B/ERV3-16A3_I, L1, L2, MLT1, THE1, Charlie, Tigger, MST*, and *MER5* sequences. The presence of the *LTR16B/ERV3-16A3_I* sequences at the XO and recombination sites is not surprising since this RE was associated with various genomic rearrangements including XO events of *MIC, HERV16*, and *HLA* class I genes, which influenced the structural organisation of the MHC locus during primate evolution (Kulski et al., 1997, 1999a,b, 2002b, 2004, 2005). The *LTR16B/ERV3-16A3_I* and *THE1* elements often are located together in close proximity to the XOs. Moreover, there are *ATCCATG/CATGGAT* motifs in the *MER2* and *L2* that flank the *ERV3-16A3-int* extension of the *HCP5* gene (Kulski, 2019) that is within a recombination hotspot located between the *MICA* and *MICB* genes (Table 8, Figure 4). On the other hand, the *THE1* elements in the MHC genomic region are represented by different subfamilies, and most of them lack the *ATCCATG* motif and therefore might not interact with PRDM9. Some active or young TE insertions are found in regions close to meiotic recombination sites. Therefore, the TE insertions in the MHC class I region that have yet to reach fixation such as the structurally polymorphic *Alu* and *SVA* elements (Table 1) are regional markers of insertion bias, and they are in close proximity to SNP XOs that could be active recombination hotspots (Figure 1). This implies that young TE insertion polymorphisms are relatively recent ancestral recombination hotspots; that is, the younger and more haplospecific TE insertions represent the integration and recombination sites of younger haplotype segments (Campos-Sánchez et al., 2016), whereas the fixed TE insertions, such as *SVA-16, -T26, -ER, -EG, -M21*, and *-M22* (Table 1, Figure 1), reveal the older haplotype recombination XO spots of our primate progenitors (Anzai et al., 2003, Wilming et al., 2013). The presence of SNP XOs near to or within the *HLA-B* and *HLA-C* genes (Tables 7, 8) suggests that these genes and/or neighbouring TE's *L1, Alu, L2, MLT1, MST, MER21, MER41, LTR9, MER1*, and *MER4* (Kulski et al., 1997) have had a role in recombination (Cullen et al., 2002, Lam et al., 2013) along with the occasional gene conversions (Madrigal et al., 1993, Adamek et al., 2015). Many of these old elements may now contribute to recombination suppression. Further detailed sequence multiple alignment studies at SNP XOs using *HLA-B/HLA-C* recombinant haplotypes such as previously described by Nair et al. (2006) in their psoriasis association studies may help to resolve this consideration.

The genomic sequences that we analysed in this study have important implications in medical research and treatment (Lokki and Paakkanen, 2019). Genotyping SNPs of the five major HLA class I and class II loci for cross matching provides most of the haplotype information needed for successful transplantation outcomes. However, ignoring SNPs outside the five loci when comparing the same or similar haplotypes may be problematic and misleading and result in choosing the wrong SNP markers for GWAS of disease or phenotypes, although GWAS need to be correlated to the MHC haplotype and not to the SNP *per se*. Nevertheless, particular haplospecific segments can be used to identify likely “disease” genes or regions (Lokki and Paakkanen, 2019) in comparative haplomics as previously performed for the psoriasis gene (Nair et al., 2006). Haplotypic or haplospecific markers like the well-defined HLA alleles and dimorphic RE markers may assist in narrowing genomic segments and loci towards MHC disease regions. Also, the haplospecific and haplotypic regions of the non-HLA coding regions such as the ~39 non-HLA genes between *HLA-A* and *HLA-C* are still poorly defined and need to be better characterised as essential components of haplotype regulatory modules. We found some SNP XO junctions in the non-HLA gene region between *HLA-E* and *HLA-C* that might have important implications in affecting disease. The systematic comparison of various recombinant haplotypes (Nair et al., 2006) is a promising approach that has been little utilised in GWAS (Alper and Larsen, 2017; Lokki and Paakkanen, 2019). Imputing haplotypes from 3D genome structures of single diploid human cells (Tan et al., 2018) along with tagging regulatory TEs using chromosome conformation capture techniques (Raviram et al., 2018) might be new and productive technical approaches to investigate haplotype regulatory modules.

## Conclusion

Our study confirms that the genomic sequences of MHC homozygous cell lines are useful for analysing MHC haplotypic landscapes and characterising unique CPS, haplotypic markers, and XO zones without a need to use LD or other probabilistic statistical imputations. Comparative sequence analyses confirmed the identity of 12 haplotypic RE markers and revealed that the *HERVK9* indel within the alpha block and a *SVA* indel within the beta block divided the *HLA-A/B/C* haplotypes into a series of distinctly interrelated historical lineages, and we identified numerous ancestral segmental XOs between different haplotypes within various REs, lncRNA, *MUC22, C6orf15*, and *HLA-C* and/or *HLA–B* genes extending over 2 Mb from the *HLA-A* to the *MICB* loci. It is evident from this study and previous studies that there is a vast MHC haplotype and allelic diversity in the human and that we have captured only a fraction of the complexity. Here, we analysed and characterised the polymorphic REs and the duplicated copies of MHC class I genes within genomic sequences of 95 haplotypes that were sequenced and assembled previously by Norman et al. (2017) in order to broaden the framework of the reference sequences so that they can be further improved and utilised to interrogate the human MHC in greater detail. More attention than usual was given to the polymorphic TE and RE at the SNP-density XOs as potential recombination hotspots. A greater emphasis on the commonality and differences of MHC class I recombinants may set the scene for better functional studies involving the described MHC alleles and haplotypes and their role in immunity, transplantation and overall health and well-being.

## Data Availability Statement

All datasets generated for this study are included in the article/Supplementary Material.

## Ethics Statement

Ethical review and approval was not required for the study on human participants in accordance with the local legislation and institutional requirements. Written informed consent for participation was not required for this study in accordance with the national legislation and the institutional requirements.

## Author Contributions

JK carried out the analyses of the repeat elements, SNP-density XOs and interpretation of the data, and wrote the manuscript. SS and TS analyzed and interpreted parts of the data and provided the alleles for the non-classical HLA class I genes, HLA pseudogenes, and the *MICA* and *MICB* genes. All authors checked the final version of the paper.

## Conflict of Interest

The authors declare that the research was conducted in the absence of any commercial or financial relationships that could be construed as a potential conflict of interest.
