# Supplementary material for: SNP-Density Crossover Maps of Polymorphic Transposable Elements and HLA Genes Within MHC Class I Haplotype Blocks and Junction
Source: Front Genet. 2021 Jan 18;11:594318. doi: 10.3389/fgene.2020.594318 (PMC7848197; doi:10.3389/fgene.2020.594318)
Supplement: Supplementary file 3 [file Table_3.DOCX]

**Table S3.** GRch38 MHC haplotype with HLA and MIC alleles, gene locations and number of alleles at each gene locus

| HLA gene or | HLA-allele | genomic location Chr6, NCBI* | Gene ID | Number of alleles |
| --- | --- | --- | --- | --- |
|  | in GRch38 |  |  |  |
| pseudogene [P] |  |  |  | for each gene** |
| HLA-F | F*01:03:01:01 | 29723340-29740355 | 3134 | 44 |
| HLA-V [P] | V*01:01:01:01 | 29791906-29797807 | 352962 | 3 |
| HLA-P [P] | P*02:01:01:02 | 29800044-29803079 | 352963 | 5 |
| HLA-G | G*01:01:01:05 | 29826979-29831130 | 3135 | 69 |
| HLA-H [P] | H*02:04 | 29887573-29891079 | 3136 | 25 |
| HLA-T [P] | T*03:01 | 29896443-29898947 | 352964 | 8 |
| HLA-K [P] | K*01:01:01:01 | 29926659-29929825 | 3138 | 6 |
| HLA-U [P] | U*01:04 | 29933764-29934880 | 352965 | 5 |
| HLA-A | A*03:01:01:01 | 29942532-29945870 | 3105 | 5.907 |
| HLA-W [P] | W*01:01:01:05 | 29955834-29959058 | 352966 | 11 |
| HLA-J [P] | J*01:01:01:04 | 30005971-30009956 | 3137 | 9 |
| HLA-E | E*01:03:02:01 | 30489508-30494194 | 3133 | 84 |
| HLA-C | C*07:02:01:03 | 31268749-31272092, comp | 3107 | 5,709 |
| HLA-B | B*07:02:01:01 | 31353875-31357179, comp | 3106 | 7,126 |
| MICA | MICA*008:04 | 31400711-31415315 | 100507436 | 159 |
| MICB | MICB*004:01:01 | 31494881-31511124 | 4277 | 109 |
| HLA-DRB5 | DRB5*01:01:01:01 | 32517343-32530316, comp | 3127 | 123 |
| HLA-DRB1 | DRB1*15:01:01:01 | 32578769-32589836, comp | 3123 | 2,690 |
| HLA-DQA1 | DQA1*01:02:01:01 | 32637406-32654846 | 3117 | 229 |
| HLA-DQB1 | DQB1*06:02:01:01 | 32659467-32666657, comp | 3119 | 1,795 |
| HLA-DPA1 | DPA1*01:03:01:02 | 33064569-33080748 | 3113 | 168 |
| HLA-DPB1 | DPB1*04:01:01:01 | 33075990-33089696 | 3115 | 1,537 |

*Assembly: GRch38p13 version, NC_000006.12 (https://www.ncbi.nlm.nih.gov/grc/human/regions/MHC?asm=GRCh38.p10).

** https://www.ebi.ac.uk/ipd/imgt/hla/stats.html, 7th April 2020.
