# Supplementary material for: SNP-Density Crossover Maps of Polymorphic Transposable Elements and HLA Genes Within MHC Class I Haplotype Blocks and Junction
Source: Front Genet. 2021 Jan 18;11:594318. doi: 10.3389/fgene.2020.594318 (PMC7848197; doi:10.3389/fgene.2020.594318)
Supplement: Supplementary file 6 [file Table_6.DOCX]

**Table S6.** Division of Beta block haplotypes into SVA-HB positive and negative lineages

|  | | HERVK9 |  | |  | |  | |  | |  | | MER5B | |  |  |  |  |  |  |  |  |  |  |
| --- | --- | --- | --- | --- | --- | --- | --- | --- | --- | --- | --- | --- | --- | --- | --- | --- | --- | --- | --- | --- | --- | --- | --- | --- |
|  | | MER9 | CR1 M11 | CF3 M9 | |  | | |  | |  | | LTR33 | | BF2AN |  |  |  |  |  |  |  |  |  |
| LAB | | (TTTC)n | (ACA)n | (TTCC)n | | SVA-HC | | *HLA-C* | | SVA-BC | | 9.5 kb | | (TTAT)n | | SVA-HB | *HLA-B* | (CTG)n | (TGT)n | SVA-MIC | MICA | AluP5 | MICB | AH |
| ID # | | M13 | M11 | M9 | | indel | | allele | | indel | | indel | | MSx | | indel | allele | MSa | MSb | indel | allele | indel | allele | hap |
| 3 |  | |  |  | |  | | *C*01:02:01* | | 2 | | 2 | | 42 | | 1 | *B*46:01:01* | 23 | 63 | 2 | MICA*010:01 | 1 | MICB*005:02 | 46 |
| 70 |  | |  |  | |  | | *C*01:02:01* | | 2 | | 2 | | 42 | | 1 | *B*46:01:01* | 23 | 63 | 2 | MICA*010:01 | 1 | MICB*005:02 | 46 |
| 92 | 36 | | 25 | 60/30 | | 1 | | *C*01:02:01* | | 2 | | 2 | | 42 | | 1 | *B*51:01:01* | 23 | 63 | 2 | MICA*010:01 | 1 | MICB*005:02 | 51 |
| 89 | 44 | | 25 | 54/30 | | 1 | | *C*01:02:01* | | 2 | | 2 | | 42 | | 1 | *B*54:01:01* | 20 | 60 | 1 | MICA*012:01 | 1 | MICB*005:02 | 54 |
| 59 | 36 | | 25 | 54/30 | | 1 | | *C*01:02:01* | | 2 | | 2 | | 42 | | 1 | *B*56:01:01* | 20 | 60 | 1 | MICA*012:01 | 1 | MICB*005:02 |  |
| 73 | 36 | | 25 | 46/30 | | 1 | | *C*01:02:30* | | 2 | | 2 | | 42 | | 1 | *B*15:01:01:01* | 23 | 63 | 2 | MICA*010:01 | 1 | MICB*006 |  |
| 34 | 44 | | 25 | 62/30 | | 1 | | *C*03:04:01:01* | | 1 | | 2 | | 34 | | 1 | *B*40:01:02* | 23 | 0 | 2 | MICA*008:04 | 2 | MICB*002:01 |  |
| 5 | 44 | | 40 | 50 | | 1 | | *C*05:01:01:01* | | 1 | | 2 | | 34 | | 1 | *B*18:01:01:01* | 20 | 60 | 2 | MICA*001 | 1 | MICB*005:02 | 18.2 |
| 15 | 40 | | 28 | 50 | | 1 | | *C*05:01:01:01* | | 1 | | 2 | | 34 | | 1 | *B*18:01:01:01* | 20 | 60 | 1 | MICA*001 | 1 | MICB*005:02 | 18.2 |
| 18 | 40 | | 40 | 50 | | 1 | | *C*05:01:01:01* | | 1 | | 2 | | 34 | | 1 | *B*18:01:01:01* | 20 | 60 | 2 | MICA*001 | 1 | MICB*005:02 | 18.2 |
| 25 | 44 | | 40 | 50 | | 1 | | *C*05:01:01:01* | | 1 | | 2 | | 34 | | 1 | *B*18:01:01:01* | 20 | 60 | 1 | MICA*001 | 1 | MICB*005:02 | 18.1 |
| 26 | 52 | | 40 | 38 | | 1 | | *C*05:01:01:01* | | 1 | | 2 | | 34 | | 1 | *B*18:01:01:01* | 20 | 60 | 2 | MICA*001 | 1 | MICB*005:02 | 18.1 |
| 9 | 64 | | 0 | 58 | | 1 | | *C*05:01:01:02* | | 1 | | 2 | | 50 | | 1 | *B*44:02:01:01* | 23 | 0 | 1 | MICA*008:01 | 1 | MICB*005:02 | 44.1 |
| 24 | 18 | | 28/59 | 50 | | 1 | | *C*05:01:01:02* | | 1 | | 2 | | 50 | | 1 | *B*44:02:01:01* | 23 | 0 | 1 | MICA*008:01 | 1 | MICB*005:02 | 44.1 |
| 60 | 60 | | 28/59 | 58 | | 1 | | *C*05:01:01:02* | | 1 | | 2 | | 50 | | 1 | *B*44:02:01:01* | 23 | 0 | 1 | MICA*008:01 | 1 | MICB*005:02 | 44.1 |
| 72 | 60 | | 28/59 | 62 | | 1 | | *C*05:01:01:02* | | 1 | | 2 | | 50 | | 1 | *B*44:02:01:01* | 23 | 0 | 1 | MICA*008:01 | 1 | MICB*005:02 | 44.1 |
| 74 | 56 | | 28/59 | 50 | | 1 | | *C*05:01:01:02* | | 1 | | 2 | | 50 | | 1 | *B*44:02:01:01* | 23 | 0 | 1 | MICA*008:01 | 1 | MICB*005:02 | 44.1 |
| 30 | 76 | | 34 | 70 | | 1 | | *C*07:01:01:01* | | 2 | | 2 | | 50 | | 1 | *B*18:01:01:02* | 20 | 60 | 2 | MICA*018:01 | 2 | MICB*002:01 | 18.1 |
| 33 | 40 | | 28 | 66 | | 1 | | *C*07:01:01:01* | | 1 | | 2 | | 50 | | 1 | *B*18:01:01:02* | 20 | 60 | 1 | MICA*018:01 | 1 | MICB*002:01 | 18.2 |
| 42 | 48 | | 34 | 46 | | 1 | | *C*07:01:01:01* | | 2 | | 2 | | 42 | | 1 | *B*49:01:01* | 26 | 66 | 1 | MICA*004 | 1 | MICB*005:02 | 49.x |
| 95 | 36 | | 25 | 66/30 | | 1 | | *C*07:01:01:01* | | 2 | | 2 | | 50 | | 1 | *B*57:01:01* | 35 | 0 | 2 | MICA*017 | 1 | MICB*003 | 57.1 |
| 4 | 60 | | 34 | 38 | | 2 | | *C*07:02:01:03* | | 1 | | 2 | | 42 | | 1 | *B*07:02:01* | 23 | 0 | deletion | MICA*008:04 | 1 | MICB*004:01 | 7.1 |
| 6 | 64 | | 34 | 38 | | 2 | | *C*07:02:01:03* | | 1 | | 2 | | 42 | | 1 | *B*07:02:01* | 23 | 0 | 2 | MICA*008:04 | 1 | MICB*004:01 | 7.1 |
| 44 | 36 | | 31 | 38 | | 1 | | *C*07:02:01:03* | | 1 | | 2 | | 42 | | 1 | *B*07:02:01* | 23 | 0 | 2 | MICA*008:04 | 1 | MICB*004:01 | 7.1 |
| 51 | 60 | | 34 | 38 | | 2 | | *C*07:02:01:03* | | 1 | | 2 | | 42 | | 1 | *B*07:02:01* | 23 | 0 | 2 | MICA*008:04 | 1 | MICB*004:01 | 7.1 |
| 52 | 60 | | 34 | 38 | | 2 | | *C*07:02:01:03* | | 1 | | 2 | | 42 | | 1 | *B*07:02:01* | 23 | 0 | 2 | MICA*008:04 | 1 | MICB*004:01 | 7.1 |
| 63 | 56 | | 31 | 38 | | 2 | | *C*07:02:01:03* | | 1 | | 2 | | 42 | | 1 | *B*07:02:01* | 23 | 0 | 2 | MICA*008:04 | 1 | MICB*004:01 | 7.1 |
| 75 | 36 | | 34 | 38 | | 2 | | *C*07:02:01:03* | | 1 | | 2 | | 42 | | 1 | *B*07:02:01* | 23 | 0 | 2 | MICA*008:04 | 1 | MICB*004:01 | 7.1 |
| 90 | 64 | | 34 | 38 | | 2 | | *C*07:02:01:03* | | 1 | | 2 | | 42 | | 1 | *B*07:02:01* | 23 | 0 | 2 | MICA*008:04 | 1 | MICB*004:01 | 7.1 |
| 65 | 68 | | 38 | 58 | | 1 | | *C*08:02:01:01* | | 1 | | 2 | | 42 | | 1 | *B*14:01:01* | 23 | 63 | 2 | MICA*019:01 | 1 | MICB*005:02 | 14.x |
| 49 | 32 | | 25 | 62/30 | | 1 | | *C*08:02:01:01* | | 1 | | 2 | | 30 | | 1 | *B*14:02:01* | 26 | 66 | 1 | MICA*011 | 1 | MICB*005:02 | 14.y |
| 87 | 32 | | 25 | 38/30 | | 1 | | *C*08:02:01:01* | | 1 | | 2 | | 40 | | 1 | *B*14:02:01* | 26 | 66 | 1 | MICA*011 | 2 | MICB*005:02 | 14.y |
| 56 | 28 | | 28/52 | 66 | | 1 | | *C*12:02:02* | | 1 | | 2 | | 42 | | 0 | *B*52:01:01:01* | 26 | 66 | 2 | MICA*009:01 | 1 | MICB*002:01 | 52.1 |
| 88 | 32 | | 28 | 62/31 | | 1 | | *C*14:02:01* | | 2 | | 2 | | 42 | | 1 | *B*51:01:01* | 26 | 66 | 2 | MICA*049 | 1 | MICB*005:02 | 51 |
| 50 | 32 | | 28 | 78/31 | | 1 | | *C*14:03* | | 2 | | 2 | | 42 | | 1 | *B*44:03:01* | 23 | 0 | 2 | MICA*004 | 2 | MICB*005:02 | 44 |
| 46 | 28 | | 28/52 | 62 | | 1 | | *C*15:02:01* | | 1 | | 2 | | 42 | | 1 | *B*51:01:01* | 26 | 66 | 2 | MICA*009:01 | 2 | MICB*002:01 | 51.y |
| 67 | 28 | | 28/52 | 62 | | 1 | | *C*15:02:01* | | 1 | | 2 | | 42 | | 1 | *B*51:01:01* | 26 | 66 | 2 | MICA*009:01 | 1 | MICB*005:02 | 51.x |
| 76 | 28 | | 28/52 | 62 | | 1 | | *C*15:02:01* | | 1 | | 2 | | 42 | | 1 | *B*51:01:01* | 26 | 66 | 2 | MICA*009:01 | 1 | MICB*005:02 | 51.x |
|  |  | |  |  | |  | |  | |  | |  | |  | |  |  |  |  |  |  |  |  |  |
| 39 | 28 | | 28/52 | 58 | | 1 | | *C*01:02:01* | | 1 | | 2 | | 0 | | 2* | *B*27:05:02* | 20 | 60 | 1 | MICA*007:01 | 1 | MICB*005:02 | 27.1 |
| 47 | 28 | | 28/52 | 66 | | 1 | | *C*01:02:01* | | 1 | | 2 | | 0 | | 2* | *B*27:05:02* | 20 | 60 | 1 | MICA*007:01 | 1 | MICB*005:02 | 27.1 |
| 66 | 36 | | 28/52 | 58 | | 1 | | *C*02:02:02:01* | | 1 | | 2 | | 0 | | 2 | *B*27:05:02* | 20 | 60 | 1 | MICA*007:01 | 1 | MICB*005:02 | 27.1 |
| 38 | 36 | | 28/52 | 26 | | 1 | | *C*02:02:02:01* | | 1 | | 2 | | 0 | | 2 | *B*40:02:01* | 23 | 63 | 2 | MICA*027 | 1 | MICB*005:02 | 40 |
| 71 | 32 | | 28/52 | 50 | | 1 | | *C*02:02:02:01* | | 1 | | 2 | | 0 | | 2 | *B*40:02:01* | 23 | 63 | 2 | MICA*027 | 1 | MICB*013 | 40 |
| 17 | 48 | | 25 | 30/30 | | 1 | | *C*03:03:01* | | 1 | | 2 | | 43/26 | | 2 | *B*15:01:01:01* | 23 | 63 | 2 | MICA*010:01 | 1 | MICB*002:01 | 15 |
| 32 | 48 | | 25 | 70/30 | | 1 | | *C*03:03:01* | | 1 | | 2 | | 34 | | 2 | *B*15:01:01:01* | 20 | 60 | 1 | MICA*010:01 | 1 | MICB*002:01 | 15 |
| 28 | 48 | | 25 | 54/30 | | 1 | | *C*03:03:01* | | 1 | | 2 | | 34 | | 2 | *B*15:01:01:01* | 23 | 63 | 2 | MICA*010:01 | 1 | MICB*005:02 | 15 |
| 40 | 52 | | 25 | 70/30 | | 1 | | *C*03:04:01:01* | | 1 | | 2 | | 34 | | 2* | *B*15:01:01:01* | 23 | 63 | 2 | MICA*010:01 | 1 | MICB*002:01 | 62.1 |
| 41 | 52 | | 25 | 50/30 | | 1 | | *C*03:04:01:01* | | 1 | | 2 | | 34 | | 2 | *B*15:01:01:01* | 23 | 63 | 2 | MICA*010:01 | 1 | MICB*002:01 | 62.1 |
| 85 | 52 | | 25 | 70/30 | | 1 | | *C*03:04:01:01* | | 1 | | 2 | | 34 | | 2* | *B*15:01:01:01* | 23 | 63 | 2 | MICA*010:01 | 1 | MICB*002:01 | 62.1 |
| 86 | 48 | | 25 | 58/30 | | 1 | | *C*03:04:01:01* | | 1 | | 2 | | 34 | | 2 | *B*40:01:02* | 23 | 0 | 2 | MICA*008:04 | 2 | MICB*002:01 | 60.x |
| 82 | 48 | | 25 | 70/30 | | 1 | | *C*03:04:01:01* | | 1 | | 2 | | 34 | | 2 | *B*40:01:02* | 23 | 0 | 2 | MICA*008:04 | 2 | MICB*004:01 | 60.y |
| 13 | 48 | | 25 | 54/30 | | 1 | | *C*03:04:01:01* | | 1 | | 2 | | 34 | | 2 | *B*40:01:02* | 23 | 0 | 2 | MICA*008:04 | 1 | MICB*014 | 60.z |
| 48 | 28 | | 28/52 | 58 | | 1 | | *C*04:01:01:01* | | 1 | | 2 | | 34 | | 2* | *B*15:26N* | 23 | 63 | 2 | MICA*010:01 | 1 | MICB*005:02 | 15.x |
| 54 | 28 | | 28/52 | 50 | | 1 | | *C*04:01:01:01* | | 1 | | 2 | | 34 | | 2 | *B*35:01:01:01* | 35 | 0 | 0 | MICA*002:01 | 1 | MICB*005:02 | 35.x |
| 35 | 32 | | 25 | 23/44 | | 1 | | *C*04:01:01:01* | | 1 | | 0 | | 34 | | 2 | *B*35:01:01:01* | 35 | 0 | 2 | MICA*017 | 1 | MICB*003 | 35.y |
| 1 | 28 | | 28/52 | 62 | | 1 | | *C*04:01:01:01* | | 0 | | 2 | | 34 | | 2* | *B*35:01:01:02* | 35 | 0 | 1 | MICA*002:01 | 2 | MICB*002:01 | 35.2 |
| 23 | 28 | | 28/52 | 66 | | 1 | | *C*04:01:01:01* | | 1 | | 2 | | 34 | | 2 | *B*35:02:01* | 23 | 63 | 2 | MICA*016 | 1 | MICB*005:01 | 35.z |
| 45 | 28 | | 28/52 | 66 | | 1 | | *C*04:01:01:01* | | 1 | | 2 | | 34 | | 2 | *B*35:02:01* | 23 | 63 | 2 | MICA*016 | 1 | MICB*005:01 | 35.z |
| 21 | 28 | | 28/52 | 58 | | 1 | | *C*04:01:01:01* | | 1 | | 2 | | 34 | | 2 | *B*35:03:01* | 35 | 0 | 1 | MICA*002:01 | 1 | MICB*005:02 | 35.w |
| 68 | 28 | | 0 | 46 | | 1 | | *C*04:01:01:01* | | 1 | | 2 | | 34 | | 2* | *B*35:08:01* | 23 | 63 | 2 | MICA*016 | 2 | MICB*002:01 | 35.v |
| 61 | 28 | | 28/52 | 62 | | 1 | | *C*04:01:01:01* | | 1 | | 2 | | 34 | | 2* | *B*53:01:01* | 26 | 66 | 1 | MICA*002:01 | 2 | MICB*006 | 53.x |
| 55 | 32 | | 25 | 50/30 | | 1 | | *C*06:02:01:01* | | 1 | | 0 | | 34 | | 2 | *B*13:02:01* | 23 | 0 | 2 | MICA*008:01 | 2 | MICB*005:02 | 13.1 |
| 57 | 32 | | 25 | 70/30 | | 1 | | *C*06:02:01:01* | | 1 | | 0 | | 34 | | 2 | *B*13:02:01* | 23 | 0 | 2 | MICA*008:01 | 2 | MICB*005:02 | 13.1 |
| 91 | 32 | | 25 | 58/30 | | 1 | | *C*06:02:01:01* | | 1 | | 0 | | 34 | | 2 | *B*13:02:01* | 23 | 0 | 2 | MICA*008:01 | 2 | MICB*005:02 | 13.1 |
| 43 | 32 | | 25 | 54/30 | | 1 | | *C*06:02:01:01* | | 1 | | 0 | | 34 | | 2* | *B*37:01:01* | 23 | 63 | 2 | MICA*010:01 | 2 | MICB*002:01 | 37.x |
| 31 | 32 | | 25 | 54/30 | | 1 | | *C*06:02:01:01* | | 1 | | 0 | | 34 | | 2 | *B*40:01:02* | 23 | 0 | 2 | MICA*008:04 | 2 | MICB*004:01 | 40.x |
| 77 | 32 | | 25 | 62/30 | | 1 | | *C*06:02:01:01* | | 1 | | 0 | | 34 | | 2 | *B*47:01:01:01* | 23 | 0 | 1 | MICA*008:01 | 1 | MICB*004:01 | 47.1 |
| 2 | 32 | | 25 | 66/30 | | 1 | | *C*06:02:01:01* | | 1 | | 0 | | 42 | | 2* | *B*57:01:01* | 35 | 0 | 2 | MICA*017 | 1 | MICB*003 | 57.1 |
| 36 | 32 | | 25 | 23/44 | | 1 | | *C*06:02:01:01* | | 0 | | 0 | | 34 | | 2 | *B*57:01:01* | 35 | 0 | 2 | MICA*017 | 1 | MICB*003 | 57.1 |
| 37 | 32 | | 25 | 66/30 | | 1 | | *C*06:02:01:01* | | 1 | | 0 | | 34 | | 2 | *B*57:01:01* | 35 | 0 | 2 | MICA*017 | 1 | MICB*003 | 57.1 |
| 58 | 32 | | 25 | 66/30 | | 1 | | *C*06:02:01:01* | | 1 | | 0 | | 34 | | 2 | *B*57:01:01* | 35 | 0 | 2 | MICA*017 | 1 | MICB*003 | 57.1 |
| 7 | 32 | | 25 | 58/30 | | 1 | | *C*06:02:01:02* | | 1 | | 0 | | 34 | | 2 | *B*50:01:01* | 26 | 66 | 1 | MICA*009:02 | 1 | MICB*005:06 | 50.1 |
| 11 | 40 | | 25 | 70/30 | | 1 | | *C*07:01:01:01* | | 1 | | 2 | | 34 | | 2* | *B*08:01:01* | 23 | 0 | 1 | MICA*008:01 | 1 | MICB*008 | 8.1 |
| 12 | 40 | | 25 | 70/30 | | 1 | | *C*07:01:01:01* | | 1 | | 2 | | 34 | | 2 | *B*08:01:01* | 23 | 0 | 1 | MICA*008:01 | 1 | MICB*008 | 8.1 |
| 16 | 40 | | 25 | 50/30 | | 1 | | *C*07:01:01:01* | | 1 | | 2 | | 34 | | 2* | *B*08:01:01* | 23 | 0 | 1 | MICA*008:01 | 1 | MICB*008 | 8.1 |
| 19 | 40 | | 25 | 46/30 | | 1 | | *C*07:01:01:01* | | 1 | | 2 | | 34 | | 2* | *B*08:01:01* | 23 | 0 | 1 | MICA*008:01 | 1 | MICB*008 | 8.1 |
| 27 | 40 | | 25 | 70/30 | | 1 | | *C*07:01:01:01* | | 1 | | 2 | | 34 | | 2 | *B*08:01:01* | 23 | 0 | 1 | MICA*008:01 | 1 | MICB*008 | 8.1 |
| 84 | 40 | | 25 | 58/30 | | 1 | | *C*07:01:01:01* | | 1 | | 2 | | 34 | | 2* | *B*08:01:01* | 23 | 0 | 2 | MICA*008:04 | 1 | MICB*004:01 | 8.x |
| 8 | 68 | | 32 | 66 | | 1 | | *C*07:18* | | 1 | | 2 | | 34 | | 2* | *B*58:01:01* | 35 | 0 | 1 | MICA*002:01 | 2 | MICB*008 | 58.1 |
| 53 | 28 | | 0 | 54 | | 1 | | *C*12:02:02* | | 1 | | 2 | | 0 | | 2* | *B*52:01:01:01* | 26 | 66 | 2 | MICA*009:01 | 1 | MICB*005:03 | 52.1 |
| 62 | 28 | | 35 | 58 | | 1 | | *C*12:02:02* | | 1 | | 2 | | 0 | | 2 | *B*52:01:01:01* | 26 | 66 | 2 | MICA*009:01 | 1 | MICB*005:03 | 52.1 |
| 93 | 28 | | 0 | 0 | | 1 | | *C*12:02:02* | | 1 | | 2 | | 0 | | 2 | *B*52:01:01:01* | 26 | 66 | 2 | MICA*009:01 | 1 | MICB*005:03 | 52.1 |
| 20 | 40 | | 28 | 42 | | 1 | | *C*12:03:01:01* | | 1 | | 0 | | 0 | | 2 | *B*35:03:01* | 35 | 0 | 1 | MICA*002:01 | 1 | MICB*005:02 | 35.2 |
| 10 | 48 | | 28 | 42 | | 1 | | *C*12:03:01:01* | | 1 | | 0 | | 34 | | 2 | *B*38:01:01* | 35 | 0 | 1 | MICA*002:01 | 2 | MICB*002:01 | 38.x |
| 22 | 0 | | 28/59 | 42 | | 1 | | *C*12:03:01:01* | | 1 | | 0 | | 34 | | 2* | *B*38:01:01* | 35 | 0 | 1 | MICA*002:01 | 2 | MICB*002:01 | 38.x |
| 64 | 64 | | 28/59 | 42 | | 1 | | *C*12:03:01:01* | | 1 | | 0 | | 34 | | 2 | *B*38:01:01* | 35 | 0 | 1 | MICA*002:01 | 2 | MICB*002:01 | 38.x |
| 69 | 53 | | 28/59 | 42 | | 1 | | *C*12:03:01:01* | | 1 | | 0 | | 34 | | 2 | *B*38:01:01* | 35 | 0 | 1 | MICA*002:01 | 2 | MICB*002:01 | 38.x |
| 94 | 52 | | 28/59 | 46 | | 1 | | *C*12:03:01:01* | | 1 | | 0 | | 32 | | 2* | *B*51:01:01* | 26 | 66 | 2 | MICA*006 | 1 | MICB*005:02 | 51.x |
| 78 | 56 | | 25 | 70/30 | | 1 | | *C*16:01:01* | | 1 | | 2 | | 0 | | 2 | *B*44:03:01* | 26 | 66 | 1 | MICA*004 | 2 | MICB*005:02 | 44.2 |
| 83 | 48 | | 25 | 70/30 | | 1 | | *C*16:01:01* | | 1 | | 2 | | 0 | | 2 | *B*44:03:01* | 26 | 66 | 1 | MICA*004 | 2 | MICB*005:02 | 44.2 |
| 79 | 48 | | 25 | 54/30 | | 1 | | *C*16:01:01* | | 1 | | 2 | | 0 | | 2 | *B*44:03:01* | 26 | 66 | 1 | MICA*004 |  | Not included | 44.2 |
| 80 | 52 | | 25 | 78/30 | | 1 | | *C*16:01:01* | | 1 | | 2 | | 0 | | 2 | *B*45:01:01* | 35 | 0 | 2 | MICA*015 | 2 | MICB*002:01 | 45.x |
| 29 | 28 | | 37 | 34/55 | | 1 | | *C*17:01:01:02* | | 1 | | 2 | | 0 | | 2 | *B*41:01:01* | 26 | 66 | 1 | MICA*004 | 2 | MICB*005:02 | 41.x |
| 14 | 28 | | 37 | 34/55 | | 1 | | *C*17:01:01:02* | | 1 | | 2 | | 0 | | 2 | *B*42:01:01* | 26 | 66 | 1 | MICA*004 | 1 | MICB*002:01 | 42.1 |

SVA-HB2* are SVA-HB duplicated sequences within LTR10/HERVI/LTR10 rearrangements that do not correlate with any particular HLA-C lineage and therefore may be sequence assembly errors.

For SVA, Alu, and indels, allele 1 is the absence of the element and allele 2 is the presence of the element. The lengths of tandem repeats are taken from the RepeatMasker output.
