# Supplementary material for: SNP-Density Crossover Maps of Polymorphic Transposable Elements and HLA Genes Within MHC Class I Haplotype Blocks and Junction
Source: Front Genet. 2021 Jan 18;11:594318. doi: 10.3389/fgene.2020.594318 (PMC7848197; doi:10.3389/fgene.2020.594318)
Supplement: Supplementary file 7 [file Table_7.DOCX]

|  |  |  |  | Haplotypes HLA-A to MICB | |  |  |  |  |  |  | RE Haplotypes | | | | |  |
| --- | --- | --- | --- | --- | --- | --- | --- | --- | --- | --- | --- | --- | --- | --- | --- | --- | --- |
| Ancestral |  |  |  |  |  |  |  |  |  |  |  |  | | |  | |  |
| Haplotype | *HLA-A* | *HLA-J* | *HLA-E* | *HLA-C* | *HLA-B anchor* | *MIC-A* | *MIC-B* | Cell line | Lab ID | Ethnicity | *AluHG* | | *HERK9-alpha* | *AluHJ* | | *SVA-HB* | |
| AH | *allele* | allele | allele | *allele* | *allele* | allele | allele |  |  |  | *allele* | | *allele* | *allele* | | *allele* | |
| *7.1AH* | *A*03:01:01:01* | *J*01:01:01:04* | *E*01:03:02:01* | *C*07:02:01:03* | *B*07:02:01* | *MICA*008:04* | *MICB*004:01* | BM14 | 4 | CE | absent | | present | absent | | absent | |
| *7.a2AH* | *A*02:01:01:01* |  |  | *C*07:02:01:03* | *B*07:02:01* | *MICA*008:04* | *MICB*004:01* | JY | 44 | CNA |  | |  |  | | absent | |
| *7.2AH* | *A*24:02:01:01* | *J*01:01:01:02* | *E*01:01:02* | *C*07:02:01:03* | *B*07:02:01* | *MICA*008:04* | *MICB*004:01* | SA | 63 | O | absent | | present | present | | absent | |
|  |  |  |  |  |  |  |  |  |  |  |  | |  |  | |  | |
| *8.1AH* | *A*01:01:01:01* | *J*01:01:01:02* | *E*01:01:01:01* | *C*07:01:01:01* | *B*08:01:01* | *MICA*008:01* | *MICB*008* | VAVY | 11 | CE | absent | | absent | present | | present | |
| *8.a26AH* | *A*26:01:01* | *J*01:01:01:08* | *E*01:03:02:01* | *C*07:01:01:01* | *B*08:01:01* | *MICA*008:04* | *MICB*004:01* | MGAR | 84 | H | absent | | absent | present | | present | |
|  |  |  |  |  |  |  |  |  |  |  |  | |  |  | |  | |
| *13.1AH* | *A*02:01:01:01* | *Not included* | *Not included* | *C*06:02:01:01* | *B*13:02:01* | *MICA*008:01* | *MICB*005:02* | BH | 55 | CNA |  | | absent | absent | | present | |
| *13.1AH* | *A*02:01:01:01* | *J*01:01:01:05* | *E*01:01:01:01* | *C*06:02:01:01* | *B*13:02:01* | *MICA*008:01* | *MICB*005:02* | BER | 57 | CE | present | | absent | absent | | present | |
| *13.a30AH* | *A*30:01:01* | *New2* | *E*01:01:01:01* | *C*06:02:01:01* | *B*13:02:01* | *MICA*008:01* | *MICB*005:02* | LBF | 91 | CE | absent | | absent | absent | | present | |
|  |  |  |  |  |  |  |  |  |  |  |  | |  |  | |  | |
| *14.a23AH* | *A*23:01:01* | *J*01:01:01:04* | *E*01:01:01:01* | *C*08:02:01:01* | *B*14:01:01* | *MICA*019:01* | *MICB*005:02* | WT51 | 65 | CE | absent | | present | absent | | absent | |
| *14.a33AH* | *A*33:01:01* | *new* | *E*01:01:01:01* | *C*08:02:01:01* | *B*14:01:01* | *MICA*011* | *MICB*005:02* | IBW9 | 49 | CE | absent | | present | absent | | absent | |
| *14.a33AH* | *A*33:01:01* | *new* | *E*01:01:01:01* | *C*08:02:01:01* | *B*14:01:01* | *MICA*011* | *MICB*005:02* | LWAGS | 87 | CE | absent | | present | absent | | absent | |
|  |  |  |  |  |  |  |  |  |  |  |  | |  |  | |  | |
| *62.a1AH* | *A*01:01:01:01* | *J*01:01:01:02* | *E*01:01:01:01* | *C*03:03:01* | *B*15:01:01:01* | *MICA*010:01* | *MICB*005:02* | CB6B | 28 | CA |  | | absent | present | | present | |
| *62.a2:17AH* | *A*02:17:02* | *J*01:01:01:05* | *E*01:03:02:01* | *C*03:03:01* | *B*15:01:01:01* | *MICA*010:01* | *MICB*002:01* | LZL | 17 | WSAI | present | | absent | absent | | present | |
| *62.a2:17AH* | *A*02:17:02* | *J*01:01:01:05* | *E*01:03:02:01* | *C*03:03:01* | *B*15:01:01:01* | *MICA*010:01* | *MICB*002:01* | AMALA | 32 | WSAI | absent | | absent | absent | | present | |
| *62.a2AH* | *A*02:01:01:01* | *J*01:01:01:05* | *E*01:01:01:01* | *C*03:04:01:01* | *B*15:01:01:01* | *MICA*010:01* | *MICB*002:01* | BOLETH | 41 | CE | present | | absent | absent | | present | |
| *62.a2AH* | *A*02:01:01:01* | *J*01:01:01:05* | *E*01:03:02:02* | *C*03:04:01:01* | *B*15:01:01:01* | *MICA*010:01* | *MICB*002:01* | BSM | 40 | CE | present | | absent | absent | | present | |
| *62.a2AH* | *A*02:01:01:01* | *J*01:01:01:05* | *E*01:03:02:02* | *C*03:04:01:01* | *B*15:01:01:01* | *MICA*010:01* | *MICB*002:01* | MCF/MLF | 85 | CE | present | | absent | absent | | present | |
| *62.a31AH* | *A*31:01:02* | *New1* | *E*01:01:01:03* | *C*01:02:30* | *B*15:01:01:01* | *MICA*010:01* | *MICB*006* | SPL | 73 | WSAI | absent | | present | absent | | absent | |
| *62.a34AH* | *A*24:02:01:01* | *J*01:01:01:04* | *E*01:01:01:03* | *C*04:01:01:01* | *B*15:26N* | *MICA*010:01* | *MICB*005:02* | ISH3 | 48 | O | absent | | present | absent | | present | |
|  |  |  |  |  |  |  |  |  |  |  |  | |  |  | |  | |
| *18.a2c7AH* | *A*02:01:01:01* | *J*01:01:01:05* | *E*01:03:02:01* | *C*07:01:01:01* | *B*18:01:01:02* | *MICA*018:01* | *MICB*002:01* | BM16 | 30 | CE | present | | absent | absent | | absent | |
| *18.a2c7AH* | *A*02:01:01:01* | *J*01:01:01:05* | *E*01:03:02:01* | *C*07:01:01:01* | *B*18:01:01:02* | *MICA*018:01* | *MICB*002:01* | 31227ABO | 33 | CE | absent | | absent | absent | | absent | |
| *18.a2c5AH* | *A*02:01:01:01* | *J*01:01:01:05* | *E*01:03:02:01* | *C*05:01:01:01* | *B*18:01:01:01* | *MICA*001* | *MICB*005:02* | JVM | 5 | CE | present | | absent | absent | | absent | |
| *18.a3AH* | *A*03:01:01:01* | *J*01:01:01:02* | *E*01:03:01:01* | *C*05:01:01:01* | *B*18:01:01:01* | *MICA*001* | *MICB*005:02* | LO081785 | 18 | CA | absent | | absent | present | | absent | |
| *18.a26AH* | *A*26:01:01* | *J*01:01:01:08* | *E*01:01:01:01* | *C*05:01:01:01* | *B*18:01:01:01* | *MICA*001* | *MICB*005:02* | QBL | 15 | CE | absent | | absent | absent | | absent | |
| *18.2AH* | *A*30:02:01:01* | *J*01:01:01:04* | *E*01:03:02:01* | *C*05:01:01:01* | *B*18:01:01:01* | *MICA*001* | *MICB*005:02* | EJ32B | 25 | CA | present | | absent | absent | | absent | |
| *18.2AH* | *A*30:02:01:01* | *J*01:01:01:04* | *E*01:03:02:01* | *C*05:01:01:01* | *B*18:01:01:01* | *MICA*001* | *MICB*005:02* | DUCAF | 26 | CE | present | | absent | absent | | absent | |
|  |  |  |  |  |  |  |  |  |  |  |  | |  |  | |  | |
| *27.1AH* | *A*02:01:01:01* | *J*01:01:01:05* | *E*01:03:02:01* | *C*01:02:01* | *B*27:05:02* | *MICA*007:01* | *MICB*005:02* | BTB | 39 | CE | present | | absent | absent | | present | |
| *27.1AH* | *A*02:01:01:01* | *J*01:01:01:05* | *E*01:01:01:01* | *C*01:02:01* | *B*27:05:02* | *MICA*007:01* | *MICB*005:02* | JESTHOM | 47 | CE | present | | absent | absent | | present | |
| *27.c2AH* | *A*02:01:01:01* | *J*01:01:01:06* | *E*01:01:01:02/11* | *C*02:02:02:01* | *B*27:05:02* | *MICA*007:01* | *MICB*005:02* | WT24 | 66 | CE | present | | absent | absent | | present | |
|  |  |  |  |  |  |  |  |  |  |  |  | |  |  | |  | |
| *35.a31AH* | *A*31:01:02* | *J*01:01:01:05* | *E*01:03:02:01* | *C*04:01:01:01* | *B*35:01:01:01* | *MICA*017* | *MICB*003* | DEU | 35 | CE | present | | absent | absent | | present | |
| *35.a2AH* | *A*02:01:01:01* | *J*01:01:01:05* | *E*01:01:01:01* | *C*12:03:01:01* | *B*35:03:01* | *MICA*002:01* | *MICB*005:02* | KOSE | 20 | CE | deletion | | absent | absent | | present | |
| *35.a2AH* | *A*02:01:01:01* | *J*01:01:01:04* | *E*01:01:01:01* | *C*04:01:01:01* | *B*35:01:01:01* | *MICA*002:01* | *MICB*005:02* | BM9 | 54 | CE | present | | absent | absent | | present | |
| *35.2AH* | *A*11:01:01* | *J*01:01:01:04* | *E*01:01:01:01* | *C*04:01:01:01* | *B*35:03:01* | *MICA*002:01* | *MICB*005:02* | KGU | 21 | CE | absent | | present | absent | | present | |
| *35.2AH* | *A*11:01:01* | *J*01:01:01:04* | *E*01:01:01:01* | *C*04:01:01:01* | *B*35:01:01:02* | *MICA*002:01* | *MICB*002:01* | WT100BIS | 1 | CE | absent | | present | absent | | present | |
| *35.5AH* | *A*01:01:01:01* | *J*01:01:01:02* | *E*01:01:01:01* | *C*04:01:01:01* | *B*35:02:01* | *MICA*016* | *MICB*005:01* | FPAF | 23 | CE | absent | | absent | present | | present | |
| *35.5AH* | *A*01:01:01:01* | *J*01:01:01:02* | *E*01:01:01:01* | *C*04:01:01:01* | *B*35:02:01* | *MICA*016* | *MICB*005:01* | J0528239 | 45 | CE | absent | | absent | present | | present | |
| *35.4AH* | *A*24:02:01:01* | *J*01:01:01:02* | *E*01:01:01:01* | *C*04:01:01:01* | *B*35:08:01* | *MICA*016* | *MICB*002:01* | TISI | 68 | CE | absent | | present | present | | present | |
|  |  |  |  |  |  |  |  |  |  |  |  | |  |  | |  | |
| *44.a32AH* | *A*32:01:01* | *J*01:01:01:06* | *E*01:01:01:09* | *C*05:01:01:02* | *B*44:02:01:01* | *MICA*008:01* | *MICB*005:02* | WT47 | 9 | CE | absent | | absent | present | | absent | |
| *44.a32AH* | *A*32:01:01* | *J*01:01:01:06* | *E*01:01:01:01* | *C*05:01:01:02* | *B*44:02:01:01* | *MICA*008:01* | *MICB*005:02* | SSTO | 72 | CNA | absent | | absent | present | | absent | |
| *44.1AH* | *A*02:01:01:01* | *J*01:01:01:05* | *E*01:01:01:01* | *C*05:01:01:02* | *B*44:02:01:01* | *MICA*008:01* | *MICB*005:02* | EK | 24 | CE | present | | absent | absent | | absent | |
| *44.1AH* | *A*02:01:01:01* | *J*01:01:01:05* | *E*01:01:01:01* | *C*05:01:01:02* | *B*44:02:01:01* | *MICA*008:01* | *MICB*005:02* | AWELLS | 60 | CA | present | | absent | absent | | absent | |
| *44.1AH* | *A*02:01:01:01* | *J*01:01:01:05* | *E*01:01:01:01/11* | *C*05:01:01:02* | *B*44:02:01:01* | *MICA*008:01* | *MICB*005:02* | SPO010 | 74 | CE | present | | absent | absent | | absent | |
| *44.2AH* | *A*29:02:01:01* | *J*01:01:01:01* | *E*01:03:02:01* | *C*16:01:01* | *B*44:03:01* | *MICA*004* | *MICB*005:02* | PITOUT | 78 | CSA | absent | | absent | absent | | present | |
| *44.2AH* | *A*29:02:01:01* | *J*01:01:01:01* | *E*01:03:02:01* | *C*16:01:01* | *B*44:03:01* | *MICA*004* | *Not included* | PF97387 | 79 | CE | absent | | absent | absent | | present | |
| *44.2AH* | *A*29:02:01:01* | *J*01:01:01:01* | *E*01:03:02:01* | *C*16:01:01* | *B*44:03:01* | *MICA*004* | *MICB*005:02* | MOU/MANN | 83 | CE | absent | | absent | absent | | present | |
| *44.4AH* | *A*33:03:01* | *New1* | *E*01:03:01:02* | *C*14:03* | *B*44:03:01* | *MICA*004* | *MICB*005:02* | HOR | 50 | O | absent | | present | absent | | absent | |
|  |  |  |  |  |  |  |  |  |  |  |  | |  |  | |  | |
| *51.a2:04.AH* | *A*02:04* | *J*01:01:01:05* | *E*01:01:01:03* | *C*15:02:01* | *B*51:01:01* | *MICA*009:01* | *MICB*005:02* | WAR | 67 |  | present | | absent | absent | | absent | |
| *51.a2:04.AH* | *A*02:04* | *J*01:01:01:05* | *E*01:01:01:03* | *C*15:02:01* | *B*51:01:01* | *MICA*009:01* | *MICB*005:02* | RML | 76 | WSAI | present | | absent | absent | | absent | |
| *51.a2:12AH* | *A*02:12* | *J*01:01:01:05* | *E*01:01:01:01* | *C*01:02:01* | *B*51:01:01* | *MICA*010:01* | *MICB*005:02* | KRC005 | 92 | SAI | present | | absent | absent | | absent | |
| *51.a24AH* | *A*24:02:01:01* | *J*01:01:01:02* | *E*01:03:02:01* | *C*12:03:01:01* | *B*51:01:01* | *MICA*006* | *MICB*005:02* | KAS116 | 94 | CE | absent | | present | present | | present | |
| *51.a31* | *A*31:01:02* | *New1* | *E*01:03:01:01* | *C*15:02:01* | *B*51:01:01* | *MICA*009:01* | *MICB*002:01* | JHAF | 46 | CE | absent | | present | absent | | absent | |
|  |  |  |  |  |  |  |  |  |  |  |  | |  |  | |  | |
| *52.1AH* | *A*24:02:01:01* | *J*01:01:01:02* | *E*01:03:01:01* | *C*12:02:02* | *B*52:01:01:01* | *MICA*009:01* | *MICB*005:03* | AKIBA | 62 | O | absent | | present | present | | present | |
| *52.1AH* | *A*24:02:01:01* | *J*01:01:01:02* | *E*01:03:01:01* | *C*12:02:02* | *B*52:01:01:01* | *MICA*009:01* | *MICB*005:03* | KAWASAKI | 93 | O | absent | | present | present | | present | |
| *52.a1AH* | *A*01:01:01:01* | *J*01:01:01:02* | *E*01:01:01:03* | *C*12:02:02* | *B*52:01:01:01* | *MICA*009:01* | *MICB*005:03* | E4181324 | 53 | CA | absent | | absent | present | | present | |
|  |  |  |  |  |  |  |  |  |  |  |  | |  |  | |  | |
| *57.a1AH* | *A*01:01:01:01* | *J*01:01:01:02* | *E*01:01:01:01* | *C*06:02:01:01* | *B*57:01:01* | *MICA*017* | *MICB*003* | WIN | 2 | CE | absent | | absent | present | | present | |
| *57.1AH* | *A*02:01:01:01* | *Not included* | *Not included* | *C*06:02:01:01* | *B*57:01:01* | *MICA*017* | *MICB*003* | DEM | 36 | CE |  | |  |  | | present | |
| *57.1AH* | *A*02:01:01:01* | *J*01:01:01:02* | *E*01:01:01:01* | *C*06:02:01:01* | *B*57:01:01* | *MICA*017* | *MICB*003* | DBB | 37 | CNA | present | | absent | present | | present | |
| *57.1AH* | *A*02:01:01:01* | *J*01:01:01:02* | *E*01:01:01:01* | *C*06:02:01:01* | *B*57:01:01* | *MICA*017* | *MICB*003* | BEI | 58 | U | present | | absent | present | | present | |
| *57.c7AH* | *A*02:01:01:01* | *J*01:01:01:05* | *E*01:01:01:01* | *C*07:01:01:01* | *B*57:01:01* | *MICA*017* | *MICB*003* | WJR076 | 95 | CNA | present | | absent | absent | | absent | |
|  |  |  |  |  |  |  |  |  |  |  |  | |  |  | |  | |
| AH of cell lines taken from Dorak et al (2006) and the AH nomenclature using B allele from Degli-Esposti et al (1992). | | | | | | |  |  |  |  |  |  | | |  | |  |
| 14.1AH? Is not an officially accepted AH designation, nor are the AH subsclass numbers labelled with the lower case letter and number (.a2) as in 7.a2AH or 8.a26AH. | | | | | | | | | |  |  |  | | |  | |  |
| The ethnicity of the cell lines is taken from EMBL-EBI at the IPD- IMGT/HLA cell query site at https://www.ebi.ac.uk/ipd/imgt/hla/ihw_cell_query.html. | | | | | | | | |  |  |  |  | | |  | |  |
| The ethnicity abbreviations are Caucasoid European (CE), Caucasoid Australian (CA), Caucasoid North American (CNA), Caucasoid South African (CSA), Hispanic North American (H), Oriental (O), South American Indian (SAI), | | | | | | | | | | | | | | | | |  |
| Warao South American Indian (WSAI) and unknown (U). | | | |  |  |  |  |  |  |  |  |  | | |  | |  |

**Supplementary Table S7.** Examples of segmental shuffling between HLA class I genes A, B, C and E, pseudogene HLA-J, and MIC genes of different ancestral haplotypes (AH) and subtypes using *HLA-B*07, -B*08, -B*13, -B*14, -B*15, -B*18, -B*27, -B*35, -B*44, -B*51, -B*52* and *-B*57* alleles as AH anchor points.
