# Supplementary material for: SNP-Density Crossover Maps of Polymorphic Transposable Elements and HLA Genes Within MHC Class I Haplotype Blocks and Junction
Source: Front Genet. 2021 Jan 18;11:594318. doi: 10.3389/fgene.2020.594318 (PMC7848197; doi:10.3389/fgene.2020.594318)
Supplement: Supplementary file 8 [file Table_8.DOCX]

**Supplementary Table S8.** Homologous haplotypes *HLA-A* to *MICB* (1.8 Mb) or *GPX5* to *MICB* (3 Mb).

|  |  | | |  | | **Chr6 position (Mb)** | | | | | **28.52** | |  | | **29.72** | | | | **30.48** | | | | **31.27** | | **31.55** |  |
| --- | --- | --- | --- | --- | --- | --- | --- | --- | --- | --- | --- | --- | --- | --- | --- | --- | --- | --- | --- | --- | --- | --- | --- | --- | --- | --- |
|  |  | | |  | | **Block type** | | | |  | **OR block** | |  | |  | | **Alpha bock** | | **Trim Cluster** | | | **Regulatory genes** | | **Beta block** |  |  |
|  |  | | |  | | **Block ID** | | | |  | **A** | | **B** | | **C** | | **D** | | **E** | | | **F** | **G** | **H** |  |  |
|  |  | | |  | | **Gene Range** | | | | | ***GPX5* to** | | ***ZNF311* to** | | ***MASiF* to** | | ***HLA-F* to** | | ***HLA-J* to** | | | ***HLA-E* to** | ***MUC21* to** | ***HLA-C* to** |  |  |
|  |  | | |  | |  |  |  |  |  | ***ZNF311*** | | ***MASiF*** | | ***HLA-F*** | | ***HLA-J*** | | ***HLA-E*** | | | ***MUC21*** | ***HLA-C*** | ***MICB*** |  |  |
|  |  | | |  | | **Block size** | | | |  | **500 kb** | | **500 kb** | | **201 kb** | | **300 kb** | | **470 kb** | | | **490 kb** | **286 kb** | **250 kb** |  |  |
|  |  | | |  | | **Range kb** | | | |  | **1 - 500** | | **500-1000** | | **1000-1201** | | **1201-1501** | | **1501-1971** | | | **1971-2461** | **2461-2747** | **2747-2997** |  |  |
| **Lab** | **Haplotype Alleles at** | | | | | | | | | ***CEH*** | **Number of SNPs in each block from A to H, crossovers (XO) between SNP poor regions (SPR) and SNP-rich regions (SRR)** | | | | | | | | | | | | | | **Total** |  |
| **ID** | ***HLA-A*** | | ***HLA-C*** | | | | ***HLA-B*** | | |  |  |  |  |  |  |  |  |  |  |  |  |  |  |  | **sequence** |  |
| 4 | *A*03:01:01:01* | | *C*07:02:01:03* | | | | *B*07:02:01* | | | *7.1* |  | |  | |  | |  | |  | | |  |  |  | 2939 kb |  |
| 6 | *A*03:01:01:01* | | *C*07:02:01:03* | | | | *B*07:02:01* | | | *7.1* |  | |  | | 4 | | 1 | | 2 | | | 3 | 3 | 3 | 1962 kb | Ps1* |
| 51 | *A*03:01:01:01* | | *C*07:02:01:03* | | | | *B*07:02:01* | | | *7.1* | 6 | | 3 | | 1 | | 1 | | 3 | | | 2 | 4 | 2 | 2938 kb | Ps1 |
| 75 | *A*03:01:01:01* | | *C*07:02:01:03* | | | | *B*07:02:01* | | | *7.1* | 6 | | 4 | | 2 | | 0 | | 2 | | | 2 | 8 | 9 | 2937 kb | Ps1 |
| 90 | *A*03:01:01:01* | | *C*07:02:01:03* | | | | *B*07:02:01* | | | *7.1* | 4 | | 2 | | 2 | | 2 | | 1 | | | 2 | 9 | 9 | 2942 kb | Ps1 |
| 27 | *A*01:01:01:01* | | *C*07:01:01:01* | | | | *B*08:01:01* | | | *8.1* |  | |  | |  | |  | |  | | |  |  |  | 2996 kb |  |
| 11 | *A*01:01:01:01* | | *C*07:01:01:01* | | | | *B*08:01:01* | | | *8.1* | 6 | | 6 | | 1 | | 0 | | 2 | | | 2 | 2 | 2 | 2933 kb | Ps1 |
| 12 | *A*01:01:01:01* | | *C*07:01:01:01* | | | | *B*08:01:01* | | | *8.1* | 6 | | 7 | | 2 | | 1 | | 3 | | | 1 | 3 | 3 | 3023 kb | Ps1 |
| 16 | *A*01:01:01:01* | | *C*07:01:01:01* | | | | *B*08:01:01* | | | *8.1* | 9 | | 10 | | 3 | | 1 | | 2 | | | 2 | 10 | 12 | 2948 kb | Ps1 |
| 19 | *A*01:01:01:01* | | *C*07:01:01:01* | | | | *B*08:01:01* | | | *8.1* | 7 | | 10 | | 1 | | 0 | | 2 | | | 1 | 7 | 7 | 2963 kb | Ps1 |
| 25 | *A*30:02:01:01* | | *C*05:01:01:01* | | | | *B*18:01:01:01* | | | *18.2* |  | |  | |  | |  | |  | | |  |  |  | 2988 kb |  |
| 26 | *A*30:02:01:01* | | *C*05:01:01:01* | | | | *B*18:01:01:01* | | | *18.2* | 0 | | 0 | | 0 | | 1 | | 5 | | | 3 | 2 | 9 | 2940 kb | Ps1 |
| 67 | *A*02:04* | | *C*15:02:01* | | | | *B*51:01:01* | | | *51.x* |  | |  | |  | |  | |  | | |  |  |  | 2952 kb |  |
| 76 | *A*02:04* | | *C*15:02:01* | | | | *B*51:01:01* | | | *51.x* | 0 | | 3 | | 0 | | 0 | | 1 | | | 2 | 2 | 5 | 2937 kb | Ps1 |
| 37 | *A*02:01:01:01* | | *C*06:02:01:01* | | | | *B*57:01:01* | | | *57.1* |  | |  | |  | |  | |  | | |  |  |  | 2984 kb |  |
| 58 | *A*02:01:01:01* | | *C*06:02:01:01* | | | | *B*57:01:01* | | | *57.1* | 0 | | 7 | | 2 | | 2 | | 4 | | | 1 | 0 | 0 | 2932 kb | Ps1 |
| 17 | *A*02:17:02* | | *C*03:03:01* | | | | *B*15:01:01:01* | | | *62.x* |  | |  | |  | |  | |  | | |  |  |  | 2944 kb |  |
| 32 | *A*02:17:02* | | *C*03:03:01* | | | | *B*15:01:01:01* | | | *62.x* | 2 | | 0 | | 0 | | 2 | | 0 | | | 1 | 1 | 1 | 2921 kb | Ps1 |
| 40 | *A*02:01:01:01* | | *C*03:04:01:01* | | | | *B*15:01:01:01* | | | *62.1* |  | |  | |  | |  | |  | | |  |  |  | 2941 kb |  |
| 85 | *A*02:01:01:01* | | *C*03:04:01:01* | | | | *B*15:01:01:01* | | | *62.1* | 0 | | 3 | | 1 | | 1 | | 1 | | | 6 | 6 | 5 | 2990 kb | Ps1 |
| 41 | *A*02:01:01:01* | | *C*03:04:01:01* | | | | *B*15:01:01:01* | | | *62.1* | 300 | | SRR | | SRR | | **80 XO 21** | | **XO >500** | | | **SRR XO SPR** | 6 | 6 | 2922 kb | Ps2 |
| 49 | *A*33:01:01* | | *C*08:02:01:01* | | | | *B*14:02:01* | | | *65.1* |  | |  | |  | |  | |  | | |  |  |  | 2940 kb |  |
| 87 | *A*33:01:01* | | *C*08:02:01:01* | | | | *B*14:02:01* | | | *65.1* |  | |  | | **3** | | 1 | | 3 | | | 2 | 0 | **3 XO 163** | 1923 kb | Ps3 |
| 78 | *A*29:02:01:01* | | *C*16:01:01* | | | | *B*44:03:01* | | | *44.2* |  | |  | |  | |  | |  | | |  |  |  | 2920 kb |  |
| 79 | *A*29:02:01:01* | | *C*16:01:01* | | | | *B*44:03:01* | | | *44.2* |  | |  | | 1 | | 1 | | 1 | | | 4 | 0 | 8 | 1772 kb |  |
| 83 | *A*29:02:01:01* | | *C*16:01:01* | | | | *B*44:03:01* | | | *44.2* | SRR | | SRR | | **108 XO** | | 2 | | 1 | | | 6 | 1 | 9 | 2975 kb | Ps4 |
| 23 | *A*01:01:01:01* | | *C*04:01:01:01* | | | | *B*35:02:01* | | | *35.5* |  | |  | |  | |  | |  | | |  |  |  | 2938 kb |  |
| 45 | *A*01:01:01:01* | | *C*04:01:01:01* | | | | *B*35:02:01* | | | *35.5* | 0 | | **XO SRR** | | **SRR XO** | | 0 | | 79 | | | 9 | 7 | 5 | 2938 kb | Ps5 |
| 39 | *A*02:01:01:01* | | *C*01:02:01* | | | | *B*27:05:02* | | | *27.1* |  | |  | |  | |  | |  | | |  |  |  | 2943 kb |  |
| 47 | *A*02:01:01:01* | | *C*01:02:01* | | | | *B*27:05:02* | | | *27.1* | 393 | | 365 | | **128 XO 15** | | 30 | | **XO 242** | | | >500 | 5 | 4 | 2944 kb | Ps6 |
| 24 | *A*02:01:01:01* | | *C*05:01:01:02* | | | | *B*44:02:01:01* | | | *44.1* |  | |  | |  | |  | |  | | |  |  |  | 2921 kb |  |
| 60 | *A*02:01:01:01* | | *C*05:01:01:02* | | | | *B*44:02:01:01* | | | *44.1* |  | |  | |  | | 19 | | **XO SRR** | | | SRR | **SRR XO** | 37 | 1792 kb | Ps7a |
| 74 | *A*02:01:01:01* | | *C*05:01:01:02* | | | | *B*44:02:01:01* | | | *44.1* | **SRR XO SPR** | | **SPR XO SRR** | | SRR XO | | **7** | | 6 | | | 2 | 3 | 3 | 2937 kb | Ps7b |
| 30 | *A*02:01:01:01* | | *C*07:01:01:01* | | | | *B*18:01:01:02* | | | *18.x* |  | |  | |  | |  | |  | | |  |  |  | 1912 kb |  |
| 33 | *A*02:01:01:01* | | *C*07:01:01:01* | | | | *B*18:01:01:02* | | | *18.x* |  | |  | | SRR | | **215 XO 26** | | **XO SRR XO SPR** | | | 4 | 3 | 4 | 2929 kb | Ps8 |
| 62 | *A*24:02:01:01* | | *C*12:02:02* | | | | *B*52:01:01:01* | | | *52.1* |  | |  | |  | |  | |  | | |  |  |  | 2893 kb |  |
| 93 | *A*24:02:01:01* | | *C*12:02:02* | | | | *B*52:01:01:01* | | | *52.1* | SRR | | SRR | | SRR | | **144 XO 1** | | **XO SRR XO SPR** | | | **5** | 0 | 2 | 2887 kb | Ps9 |
| 13 | *A*02:01:01:01* | | *C*03:04:01:01* | | | | *B*40:01:02* | | | *60.1* |  | |  | |  | |  | |  | | |  |  |  | 2749 kb |  |
| 86 | *A*02:01:01:01* | | *C*03:04:01:01* | | | | *B*40:01:02* | | | *60.1* | SRR | | SRR | | SRR | | **193 XO 7** | | 16 | | | **SPR XO SRR** | **XO 5** | **2 XO 117** | 2947 kb | Ps10 |
| 9 | *A*32:01:01* | | *C*05:01:01:02* | | | | *B*44:02:01:01* | | | *44.x* |  | |  | |  | |  | |  | | |  |  |  | 2937 kb |  |
| 72 | *A*32:01:01* | | *C*05:01:01:02* | | | | *B*44:02:01:01* | | | *44.x* | SRR | | SRR | | **157 XO** | | 8 | | **SPR XO SRR** | | | **SRR XO SPR** | SPR | **SPR XO SRR** | 2976 kb | Ps11 |
|  | |  | | |  | | |  |  | | |  | |  | |  | |  | |  |  |  |  |  |  |  |

*Ps1. SNP-poor homologous nucleotide sequence pairs with no XO across ~3 Mb from GPX5 to MICB.

Ps2. XO in Block D from SRR to SPR at 1235407 G/T within Charlie20 at the telomeric end of the F segment and 1.2 kb centromeric of LTR16b at the start of the V segment.

XO in block F from SRR to SPR at 2269096 T/C within a Dnase cluster (UCSC browser) between L1 and the LTR MER50 and within or near to LINC00243.

Ps3. XO in block H from SPR to SRR at 2893301 T/C within ERV3-16A3_I (HCP5) between MICA and MICB.

Ps4. XO in block C SRR to SPR at 1091424 T/A within MER20B between GABBR1 and MOG genes. MICB in block H not included in analysis.

Ps5. XO in block C SRR to SNP at 645798 C/T within AluSx adjoining MER20B between GABR1 and MOG genes.

Ps6. XO in block C SRR to SNP at 1064633 GA within AluY adjoining LTR39 between OR2H2 and GABBR1 genes.

Ps7a. XO from SPR to SRR at 1532538 T/C near TRIM31 and then a transition to SPR at 2548584 C/T near PSORORS1C1.

Ps7b. Three XO in the OR blocks: 166359 A/G in HERK-int between ZBED and TRIM27 genes, 844903 A/G between OR12D3 and OR12D2 genes, and 1045240 C/A in MER47 at 3' OR2H2.

Ps8. XO in block D SRR to SPR at 198913 G/T within Charlie20 of F segment and near the boundary of the V segment. XO in block E SPR to SRR at 513915 G/C in MLT1C2 until XO at 760342 G/A.

Ps9. XO in block D SRR to SPR at 1220877 C/G within Tigger1 at the boundary of the F and V segments. XO from SPR to SRR at 1643220 A/G within THE1 element near HLA-L/TRIM39, and XO from SRR to SPR within SVA-ER

Ps10. XO in block D SRR to SPR at 1037893 T/G within Charlie20 of F segment. XO in block F SPR to SRR at 2020192 A/C between L1ME1 and MamGypLTR near HCG20.

near HLA-E gene. XO in block G SRR to SPR at 2273704 A/G between L2 and AluY near MUC22. XO in block H at 2693882 T/C within SVA-HC insertion (6.8 kb from LTR16B and 2.6 kb from THE1C) located between MICA and MICB.

Ps11. XO in block C SRR to SPR at 1106547 T/C between GABR1 and MOG genes. XO in block E at 1786326 T/C in the region of HLA-N & UBQLN1P1/MICC; XO in block F at 2383487 A/G in the region between DPCR and MUC21.

XO in block H SPR to SRR at 2864012 G/A between L1ME and MER5A and telomeric of MICA.

CEH, conserved extended haplotypes adapted from Dorak et al (2006); XO is crossover, SPR is SNP poor region and SRR is SNP rich region. Sequence alignments between different LabID are blocked, eg, LabID4 was aligned against 6, 51, 75, 90 in the top block.
