# Supplementary material for: SNP-Density Crossover Maps of Polymorphic Transposable Elements and HLA Genes Within MHC Class I Haplotype Blocks and Junction
Source: Front Genet. 2021 Jan 18;11:594318. doi: 10.3389/fgene.2020.594318 (PMC7848197; doi:10.3389/fgene.2020.594318)
Supplement: Supplementary file 9 [file Table_9.DOCX]

| **Analysis** | **Alignments between haplotypes** | | **Number of SNPs per section (manual count)/SRR or SFR or mix with a XO** | | | |  | **XO** | **SNP or** | **Closest** | **Nearest gene** |
| --- | --- | --- | --- | --- | --- | --- | --- | --- | --- | --- | --- |
| **Number** | **Lab ID numbers precede haplotypes** | | **Blocks A to E** |  |  |  |  | **Location** | **indel** | **repeat** | **or genes at XO** |
|  | **Haplotype 1** | **Haplotype 2** | **HLA-A/HLA-J** | **HLA-J/HLA-E** | **HLA-E/MUC21** | **MUC21/PSORS** | **PSORS/HLA-C** | **bp/section** | **at XO** | **at XO** |  |
|  |  |  | **97k** | **486k** | **484-493k** | **202.8k** | **88k** |  |  |  |  |
|  |  |  | **L2/MER90** | **MER30/LTR67** | **LTR67/MER5** | **MER5/HARLE** | **MER5/L1** |  |  |  |  |
|  |  |  | **A** | **B** | **C** | **D** | **E** |  |  |  |  |
| 1 | 10_A*02-C*12:03 | 54_A*02-C*04 | **XO** | **SRR (729)** | SRR | SRR | SRR | HLA-A/-J |  |  | Seg A/W |
| 2 | 10_A*02-C*12:03 | 8_A*0205-C*07 | **XO** | **SRR XO SPR XO SRR** | SRR | SRR | SRR | HLA-A/-J |  |  | Seg A/W |
| 3 | 10_A*02-C*12:03 | 17_A*02:17:02-C*03:03 | SPR (3) | **SPR XO SRR XO SPR** | SRR | SRR | SRR | 118295/B | G/C | MIR | SNORA48/TRIM40 |
| 4 | 10_A*02-C*12:03 | 67_A*02:04-C*15:02 | SPR (0) | **SPR (15) XO (456)** | SRR | SRR | SRR | 222246/B | T/C | AluY/L1 | TRIM26BP |
| 5 | 2_A*01-C*06 | 11_A*01-C*07 | SPR | **SPR XO1 SRR** | SRR/SFR/SRR | SRR/SFR | SFR/SRR | 76366/B | C/G | (CTCC)n | ZNDR1/TRIM31 |
| 6 | 11_A*01-C*07 | 2_A*01-C*06 | SPR (4) | **SPR (3) XO1 SRR (676)** | SRR/SFR/SRR | SRR/SFR | SFR/SRR | 78018/B | G/C | (CTCC)n | ZNDR1/TRIM31 |
| 7 | 11_A*01-C*07 | 28_A*01-C*03:03 | SPR | **SPR XO1 SRR** | SRR/SFR/SRR | SRR/SFR | SFR/SRR | 78018/B | G/C | (CTCC)n | ZNDR1/TRIM31 |
| 8 | 28_A*01-C*03:03 | 11_A*01-C*07 | SPR | **SPR XO1 SRR** | SRR/SFR/SRR | SRR/SFR | SFR/SRR | 76366/B | C/G | (CTCC)n | ZNDR1/TRIM31 |
| 9 | 10_A*02-C*12:03 | 30_A*02-C*07 | SPR (0) | SPR (6) | **XO + SRR** | SRR | SRR | 250643/C | C/G | L2/L2 | IER3/HCG20 |
| 10 | 10_A*02-C*12:03 | 13_A*02-C*03:04 | SPR (0) | SPR | **XO + SRR** | SRR | SRR | 250889/C | C/A | L2/L2 | IER3/HCG20 |
| 11 | 2_A*01-C*06 | 28_A*01-C*03:03 | SPR | SPR | **XO + SRR** | SRR | SRR | 251599/C | C/A | L2/L2 | IER3/HCG20 |
| 12 | 28_A*01-C*03:03 | 2_A*01-C*06 | SPR | SPR | **XO + SRR** | SRR | SRR | 251275/C | A/C | L2/L2 | IER3/HCG20 |
| 13 | 2_A*01-C*06 | 23_A*01-C*04 | SPR | SPR | **XO + SRR** | SRR | SRR | 300807/C | A/G | L1MC5 | 3' of HCG20 |
| 14 | 23_A*01-C*04 | 2_A*01-C*06 | SPR (0) | SPR | **XO + SRR** | SRR | SRR | 301686/C | G/A | L1MC5 | 3' of HCG20 |
| 15 | 23_A*01-C*04 | 31_A*01-C*06 | SPR (0) | SPR (0) | **XO + SRR** | SRR | SRR | 301686/C | G/A | L1MC5 | 3' of HCG20 |
| 16 | 2_A*01-C*06 | 31_A*01-C*06 | SPR (0) | SPR (6) | SFR (2) | SFR (12) | SFR ( 0) |  |  |  | HLA-C/HLA-B |
| 6* | 11_A*01-C*07-B*08 | 2_A*01-C*06-B*57 | SPR (4) | SPR (3) XO1 SRR (676) |  |  |  | 78018/B | G/C | CTCC)n | ZNDR1/TRIM31 |
|  |  |  |  |  | SRR(3) XO2 SPR (21) |  |  | 7999/C | G/A | AluY/AluS | HLA-E/lncRNA 2569 |
|  |  |  |  |  | SPR (21) X03 SRR (560) |  |  | 220859/C | G/C | CCCG)n | TUBB/FLOT1 |
|  |  |  |  |  |  | SRR XO4 SPR (5) |  | 155071/D | T/A | AluY/L2 | ~PSORORS1C2 |
|  |  |  |  |  |  |  | SPR (8) XO5 (137) | 81686/E | T/C | L2/L1 | 410bp 3' HLA-C |

**Supplementary Table S9.** SNP variations and crossover (XO) loci between *HLA-A* and *HLA-C* using different haplotype DNA sequence pairs with the same *HLA-A* alleles, but different *HLA-C* alleles.

6* is expanded version of 6.

SRR is SNP rich region estimated to be >100 SNP/100k, SPR is SNP poor region (<10 SNP/100k).

XO in columns is crossover and numbers in brackets are the number of SNPs after the crossover.
