# Supplementary material for: SNP-Density Crossover Maps of Polymorphic Transposable Elements and HLA Genes Within MHC Class I Haplotype Blocks and Junction
Source: Front Genet. 2021 Jan 18;11:594318. doi: 10.3389/fgene.2020.594318 (PMC7848197; doi:10.3389/fgene.2020.594318)
Supplement: Supplementary file 10 [file Table_10.DOCX]

| **Analysis** | **Alignments between haplotypes** | | **Number of SNPs per section (manual count)/SR or SP region or mix with a XO in blocks A to E** | | | | | **Xover** | **SNP or** | **Nearest** | **Nearest** | **Breakpoint** |  |
| --- | --- | --- | --- | --- | --- | --- | --- | --- | --- | --- | --- | --- | --- |
| **Number** | **Lab ID numbers precede haplotypes** | | **HLA-A to** | **HLA-J to** | **HLA-E to** | **MUC21 to** | **PSORS to** | **Location** | **indel** | **repeat** | **gene** | **nt distance to** | **HLA-C** |
|  | **Haplotype 1** | **Haplotype 2** | **HLA-J (97k)** | **HLA-E (486k)** | **MUC21 (490k)** | **PSORS (203k)** | **HLA-C (88k)** | **bp/section** | **at XO** | **at XO** | **to XO** | **3'end of HLA-C** | **exon 8 - 3'end** |
|  |  |  | **A** | **B** | **C** | **D** | **E** |  |  |  |  |  |  |
|  | **Different HLA-A allele and same HLA-C allele** | |  |  |  |  |  |  |  |  |  |  |  |
| 1 | 95_A02-C07 | 8_A*02:05-C*07:18 | SR | SR | SR | SR | **SR (505) XO SP** | 84011/E | A/G | L2/L1 | HLA-C | (-)1345 | 82666 |
| 2 | 11_A*01-C*07 | 8_A*02:05-C*07:18 | SR | SR | SR | SR | **SR XO SP** | 83442/E | A/G | L2/L1 | HLA-C | -1345 | 82097 |
| 3 | 30_A*02-C*07 | 8_A*02:05-C*07:18 | SR | SR | SR | SR | **SR XO SP** | 81822/E | A/G | L2/L1 | HLA-C | -1335 | 80487 |
| 4 | 8_A*02:05-C*07:18 | 4_A*03-C*07 | SR | SR | SR | SR | **SR XO SP** | 83408/E | G/A | L2/L1 | HLA-C | -749 | 82659 |
| 5 | 95_A*02-C*07 | 4_A*03-C*07 | SR | SR | SR | SR | **SR XO SP** | 83138/E | C/T | L2/L1 | HLA-C | -572 | 82666 |
| 6 | 11_A*01-C*07 | 4_A*03-C*07 | SR (193) | SR | SR | SR | **SR XO SP** | 82425/E | T/C | L2/L1 | HLA-C | -329 | 82096 |
| 7 | 23_A*01-C*04 | 35_A*31-C*04 | SR | SR | SR | SR | **SR (>200*) XO SP** | 81483/E | A/G | L2/L1 | HLA-C | -162 | 81321 |
| 8 | 39_A*02-C*01 | 89_A*24-C*01:02 | SR | SR | SR | SR | **SR XO SP** | 80722/E | T/C | L2/L1 | 3'NCR HLA-C | 276 | 80998-81421 |
| 9 | 73_A*31-C*01 | 89_A*24-C*01:02 | SR | SR | SR | SR | **SR (327) XO SP** | 81303/E | C/T | L2/L1 | 3'NCR HLA-C | 276 | 81579 |
| 10 | 10_A*02-C*12 | 53_A*01-C*12 | SR | SR | SR | SR | **SR XO SP** | 81653/E | G/A | L2/L1 | 3'NCR HLA-C | 363 | 82016 |
| 11 | 11_A*01-C*07 | 30_A*02-C*07 | SR | SR | SR | SR | **SR XO SP** | 81605/E | T/C | L2/L1 | 3'NCR HLA-C | 491 | 82096 |
| 12 | 92_A*02:12-C*01 | 89_A*24-C01:02 | SR | SR | SR | SR | **SR XO SP** | 80344/E | A/G | L2/L1 | 3'NCR HLA-C | 654 | 80998 |
| 13 | 30_A*02-C*07 | 4_A*03-C*07 | SR | SR | SR | SR | **SR XO SP** | 79103/E | C/T | L2 | 3'NCR HLA-C | 1384 | 80487 |
| 14 | 23_A*01-C*04 | 61_A*68-C*04 | SR | SR | SR | SR | **SR XO SP** | 74139/E | C/A | MLT1A1 | 7kb-3' HLA-C | 7128 | 81321 |
| 15 | 39_A*02-C*01 | 92_A*02:12-C*01:02 | SP (0) | **SP XO SR** | SR | SR | **SR XO SP** | 69544/E | A/G | AluY | 11.4kb-3' HLA-C | 11454 | 80998 |
| 16 | 39_A*02-C*01 | 59_A*24-C*01:02 | SR | SR | SR | SR | **SR XO SP** | 69544/E | A/G | AluY | 11.4kb-3' HLA-C | 11454 | 80998 |
| 17 | 39_A*02-C*01 | 73_A*31-C*01:02 | SR | SR | SR | SR | **SR XO SP** | 69544/E | A/G | AluY | 11.4kb-3' HLA-C | 11454 | 80998 |
| 18 | 73_A*31-C*01 | 39_A*02-C*01:02 | SR | SR | SR | SR | **SR XO SP** | 69544/E | G/A | AluY | 11.4kb-3' HLA-C | 11454 | 81579 |
| 19 | 65_A*23-C*08 | 49_A*33-C*08 | SR | SR | SR | SR | **SR XO SP** | 70523/E | G/C | MER21-int | 11.5kb-3' HLA-C | 11493 | 81607-82029 |
| 20 | 5_A*02-C*05 | 9_A*32-C*05 | SR | SR | SR | SR | **SR (474) XO SP** | 69150/E | G/A | MER21-int | 12kb-3' HLA-C | 12093 | 81243 |
| 21 | 88_A*02-C*14 | 50_A*33-C*14 | SR | SR | SR | SR | **SR (29) XO SP** | 65401/E | C/T | L1PA13 | 9kb-3'HLA-C | 8896 | 74297-74720 |
| 22 | 28_A*01-C*03:03 | 17_A*02:17-C*03:03 | SR | SR | SR | **SR XO SP** | SP (18) | 192386/D | A/C | MIR3/AluSx | PSORS1C3 | ~95000 | 81322 |
| 23 | 28_A*01-C*03:03 | 13_A*02-C*03:04 | SR | SR | SR | **SR XO SP** | SP (21) | 192386/D | A/C | MIR3/AluSx | PSORS1C3 | ~95000 | 81322 |
| 24 | 28_A*01-C*03:03 | 34_A*24-C*03:04 | SR | SR | SR | **SR XO SP** | SP (21) | 192386/D | A/C | MIR3/AluSx | PSORS1C3 | ~95000 | 81322 |
| 25 | 13_A*02-C*03:04 | 34_A*24-C*03:04 | SR | SR | SR | **SR XO SP** | SP (0) | 192386/D | A/C | MIR3/AluSx | PSORS1C3 | ~95000 | 81322 |
| 26 | 17_A*02:17-C*03:03 | 28_A*01-C*03:03 | SR | SR | SR | **SR XO SP** | SP (8) | 188587/D | C/A | MIR3/AluSx | PSORS1C3 | ~95000 | 82943 |
| 27 | 29_A*01-C*17 | 14_A*30-C*17 | SR | SR | SR | **SR XO SP (0)** | SP (6) | 185258/D | G/A | AluJb/MIR3 | NCR of PSORS1C3 | ~100000 | 73508 |
| 28 | 92_A*02:12-C*01:02 | 73_A*31-C*01:02 | SR | SR | SR | **SR XO SP (6)** | SP (0) | 126710/D | T/A | MLT1D/L1M | NCR of C6orf15 | ~156200 | 80998 |
| 29 | 73_A*31-C*01:02 | 92_A*02:12-C*01:02 | SR | SR | SR | **SR XO SP 4)** | SP (0) | 129587/D | T/C | MLT1D | NCR of C6orf15 | ~156200 | 81579 |
| 30 | 73_A*31-C*01:02 | 59_A*24-C*01:02 | SR | SR | SR | **SR XO SP (7)** | SP (0) | 129916/D | C/T | MLT1D/L1M | NCR of C6orf15 | ~156200 | 81579 |
| 31 | 11_A*01-C*07-B*08 | 95_A*02-C*07-B*57 | SR | SR | SR | **SR (102) XO SP (3)** | SP (4) | 70836/D | C/T | THE1A/AluJ | HCG22 | ~215000 | 82097 |
| 32 | 2_A*01-C*06-B*57 | 91_A*30-C*06--B*13:02 | SR | SR | SR | **SR (173) XO SP (24)** | SP (5) | 70835/D | G/C | THE1A/AluJb | HCG22 | ~215000 | 82906 |
| 33 | 2_A*01-C*06-B*57 | 77_A*03-C*06-B*47 | SR | SR | SR | **SR XO SP (20)** | SP (7) | 70053/D | G/A | THE1A | HCG22 | ~215000 | 82906 |
| 34 | 2_A*01-C*06-B*57 | 55_A*02-C*06-B*13:02 | SR | SR | SR | **SR XO SP (26)** | SP (5) | 70835/D | G/C | THE1A/AluJb | HCG22 | ~215000 | 82906 |
| 35 | 17_A*02:17-C*03:03 | 13_A*02-C*03:04 | SP (0) | **SP XO SR** | SR | **SRXO SP (8)** | SP (2) | 49319/D | T/C | MIRb/MIRb | MUC22 | ~215000 | 82943 |
| 36 | 10_A*02-C*12:03 | 94_A*24-C*12 | SR | SR | SR | **SR (227) XO SP (4)** | SP (0) | 42687/D | T/C | L2b/AluY | MUC22 | ~215000 | 82016-82400 |
| 37 | 80_A*02-C*16 | 78_A*29-C*16 | SR | SR | **SP XO SR XO SP** | SP (13) | SP (1) | 470123/C | A/G | AluSx/HERVL-int | NCR of MUC21 | ~279000 | 86743 |
| 38 | 67_A*02:04-C*15 | 46_A*31-C*15 | SR | SR | **SR XO SP** | SP (9) | SP (0) | 469599/C | A/C | AluSx/HERVL-int | NCR of MUC21 | ~279000 |  |
| 39 | 23_A*01-C*04 | 1_A*11-C*04 | SR | SR | **SR XO SP** | SP (16) | SP (9) | 467180/C | T/C | AluSx/HERVL-int | NCR of MUC21 | ~279000 | 81321-81745 |
| 40 | 23_A*01-C*04 | 54_A*02-C*04 | SR (389) | SR | **SP XO SR XO SP** | SP (5) | SP (7) | 467104/C | A/G | AluSx/HERVL-int | NCR of MUC21 | ~279000 | 81321-81745 |
| 41 | 2_A*01-C*06 | 36_A*02-C*06 | SR | SR | **SR XO SP** | SP (0) | SP (0) | 466251/C | A/C | AluSx/HERVL-int | NCR of MUC21 | ~279000 | 82906 |
| 42 | 92_A*02:12-C*01:02 | 59_A*24-C*01 | SR | SR | **SR XO SP** | SP (9) | SP (1) | 464610/C | C/T | AluSx/HERVL-int | NCR of MUC21 | ~279000 | 80998 |
| 43 | 17_A*02:17-C*03:03 | 34_A*24-C*03:04 | SR | SR | **SP XO SR XO** | SP (14) | SP (0) | 463976/C | G/A | AluSx/HERVL-int | NCR of MUC21 | ~279000 | 82943 |
| 44 | 10_A*02-C*12:03 | 22_A*32-C*12:03 | SP (0) | SR | **SR (25) + XO + SP (27)** | SP (2) | SP (0) | 36880/C | A/G | MLT2D/L2 | LINC02569/GNL1 | ~730000 | 82016-82400 |
| 45 | 2_A*01-C*06-B*57 | 31_A*01-C*06-B*40 | SP (0) | SP (6) | SP (2) | SP (12) | SP ( 0) | SNP poor across alpha block and no XO detected | | |  |  | 82906 |
| SR is SNP rich region estimated to be >100 SNP/100k, SP is SNP poor region ( (<10 SNP/100k). XO is crossover and numbers in brackets are the number of SNPs after the crossover. NCR is non coding region. | | | | | | | | |  |  |  |  |  |

**Supplementary Table S10.** SNP variations and crossover (XO) loci between *HLA-A* and *HLA-C* within different haplotype DNA sequence pairs with the same *HLA-C* alleles, but different *HLA-A* alleles.
