# Supplementary material for: SNP-Density Crossover Maps of Polymorphic Transposable Elements and HLA Genes Within MHC Class I Haplotype Blocks and Junction
Source: Front Genet. 2021 Jan 18;11:594318. doi: 10.3389/fgene.2020.594318 (PMC7848197; doi:10.3389/fgene.2020.594318)
Supplement: Supplementary file 12 [file Data_Sheet_1.PDF]

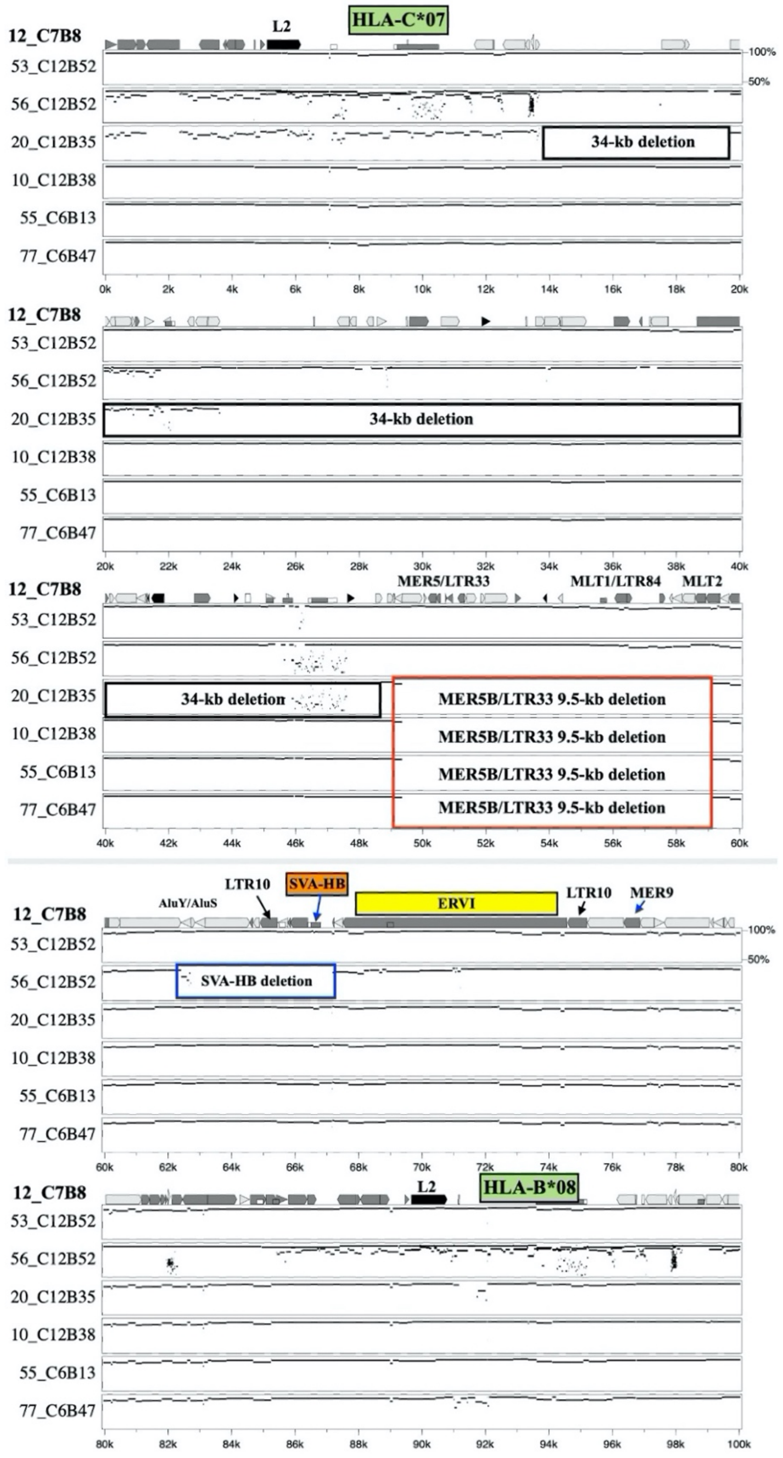

Supplementary Fig. 1. Percent Identity Plot (PIP) highlights three indels: a 34-kb deletion, a 9.5-kb *MER5/LTR33* indel and a deletion of the *SVA-HB* insertion location between the *HLA-C* and *HLA-B* loci in the alignment of 7 different haplotypes. The upper sequence represents haplotype ID12_*C*07-B*52* and the other six haplotypes are 10_*C*12-B*38* and the haplotype Lab ID numbers 53, 56, 20, 10, 55 and 77 listed in Table 7. The deletion of the *SVA-HB* insertion location is in haplotype *C*12:02:02-B*52:01*. The interspersed repeats in the upper sequence such as the *Alu, MIR, LTR* and *L1* fragments are indicated with the symbols used by Schwartz et al., (2000). The locations of *HLA-C*07* and *HLA-B*08* are indicated by the green labelled boxes and some of the noteworthy TEs are labelled.

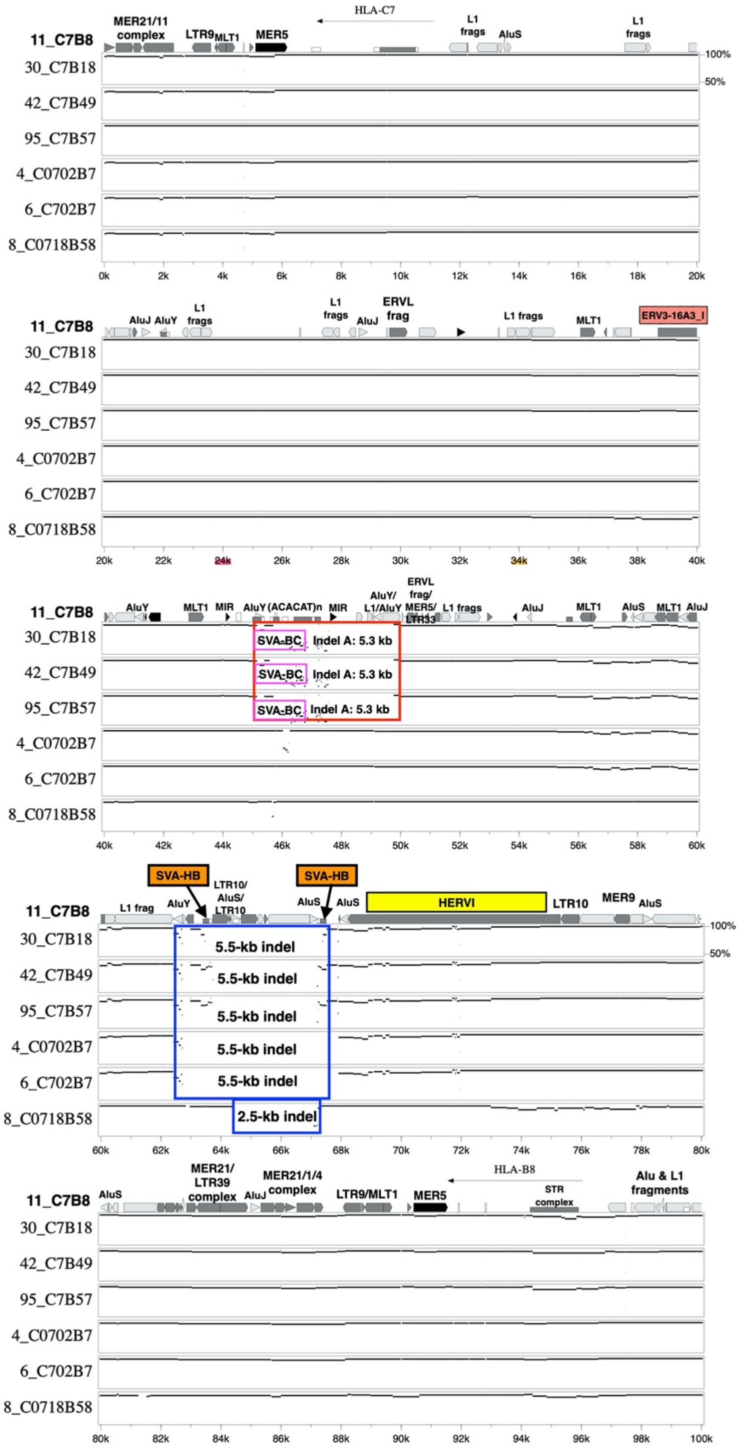

Supplementary Fig. 2. PIP highlights three types of indel > 2-kb in sequence alignments from telomeric of *HLA-C* to centromeric of *HLA-B* of seven haplotypes listed on the left side of the figure. The haplotype ID numbers 11, 30, 42, 95, 4, 6, and 8 correspond to those in Table 7. The presence of two *SVA-HB* outside the *HERVI* sequence suggests a possible sequence assembly error for 11_*C*07-B*08*. The interspersed repeats in the upper sequence such as the *Alu, MIR, LTR* and *L1* fragments are indicated with the symbols used by Schwartz et al., (2000). The locations of *HLA-C*07* and *HLA-B*08* are indicated by the labelled horizontal arrows and some noteworthy TEs are labelled. The boxed *SVA-BC* sequence is present in all of the *C*07:01* haplotypes except for ID 33_*C*07:01* and it is not part of the 5.3-kb indel A deletion in haplotype IDs 30, 42 and 95 (Table S2).

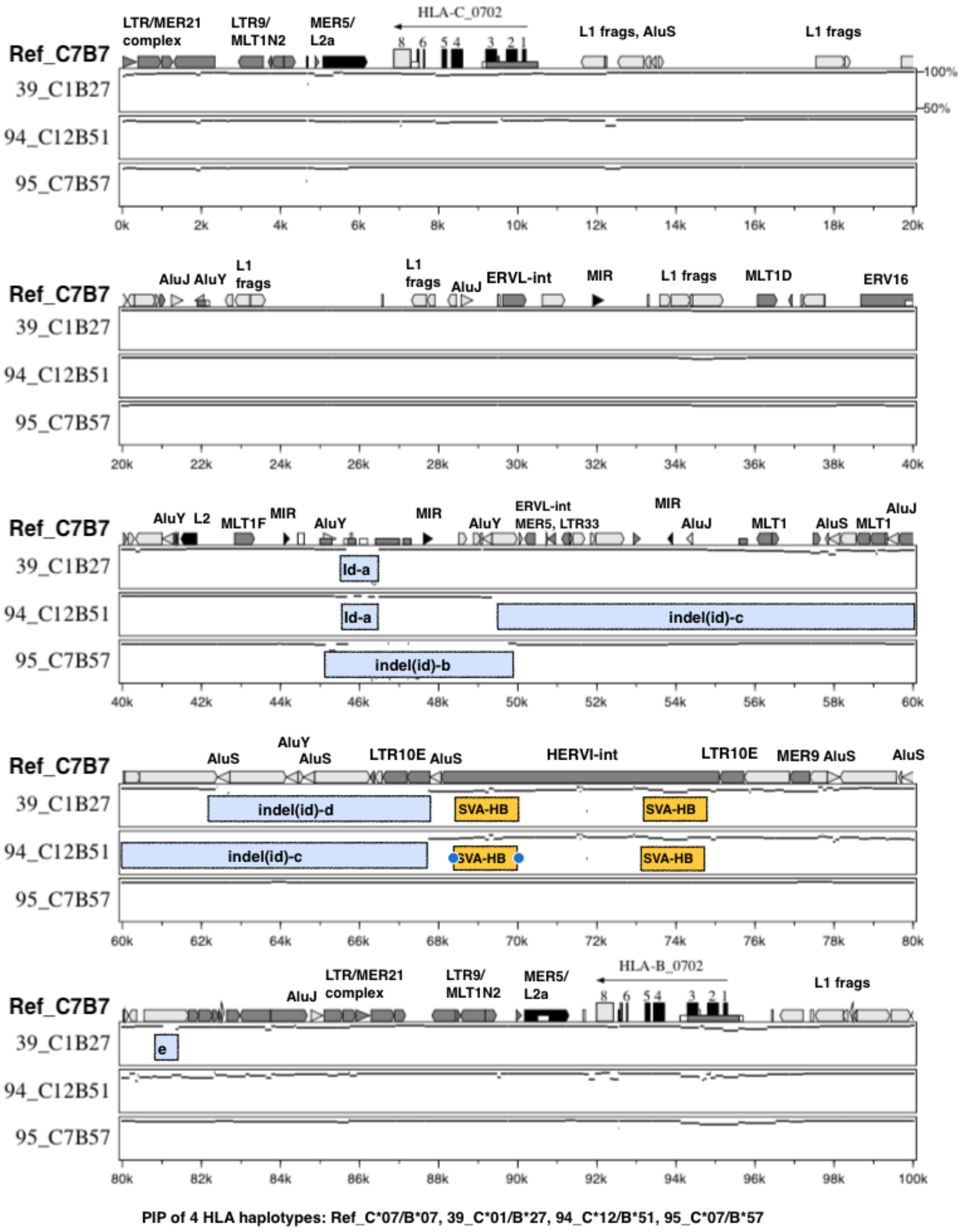

Supplementary Fig. 3. PIP highlights five types of indels (blue boxes 'a' to 'e') in sequence alignments from telomeric of *HLA-C* to centromeric of *HLA-B* of four haplotypes listed on the left side of the figure. The presence of two *SVA-HB* within the labelled *HERVI* sequence may be an assembly error for haplotypes 39_*C*01-B*12* and 94_*C*01-B*51* (Table 3 and Table S2). The interspersed repeats in the upper sequence such as the *Alu, MIR, LTR* and *L1* fragments are indicated with the symbols used by Schwartz et al., (2000). The locations of *HLA-C*07* and *HLA-B*07* are indicated by the labelled horizontal arrows. Noteworthy TEs are labelled.

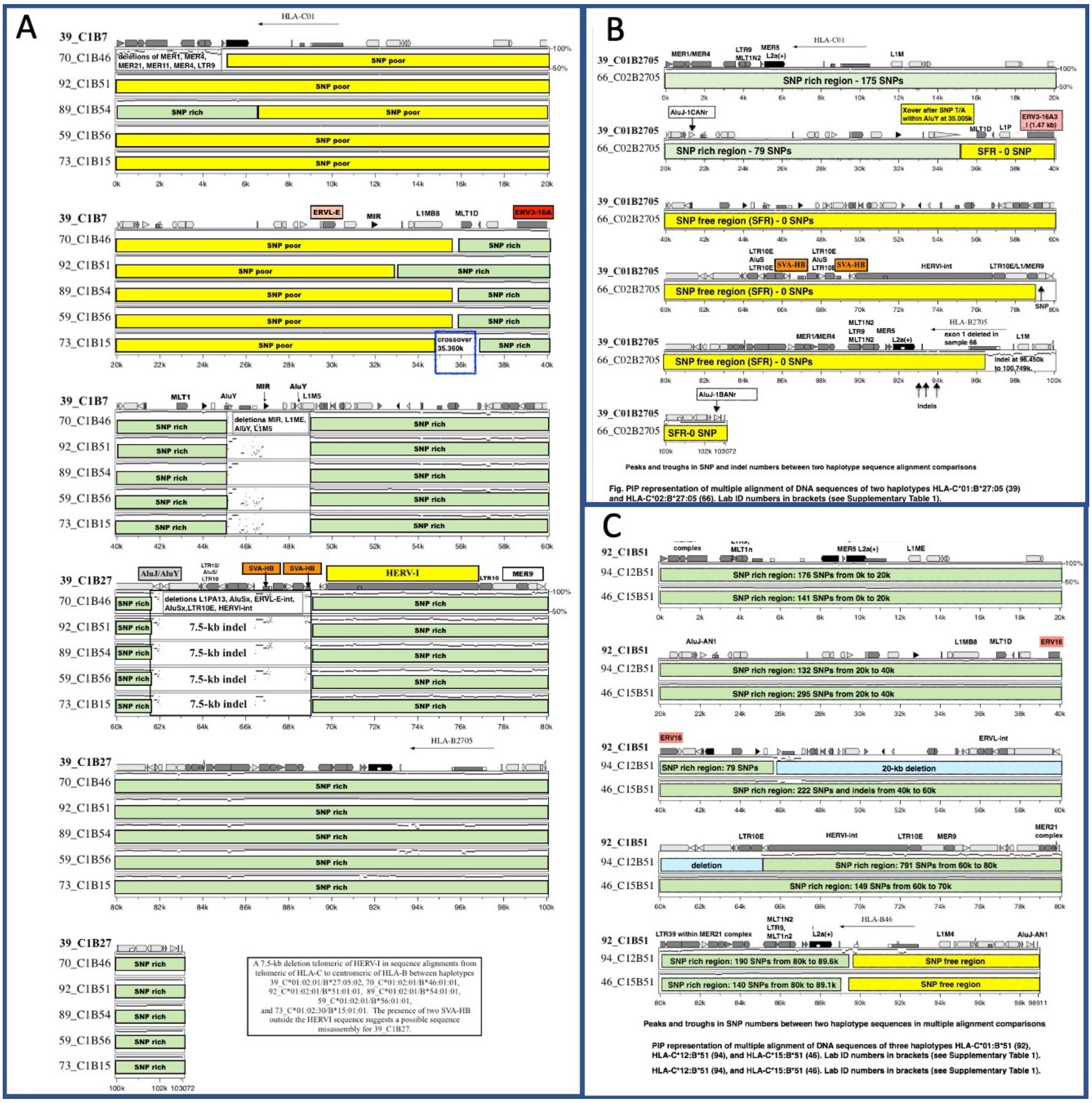

Supplementary Fig. 4. Three PIP outputs A, B and C show the locations of SNP crossovers and an additional number of different types of indels in sequence alignments from telomeric of *HLA-C* to centromeric of *HLA-B* of various haplotypes listed on the left side of each figure. (A), a 7.5-kb deletion telomeric of *HERV-I* in 5 haplotypes, 70, 92, 89, 59, 73, aligned with the upper haplotype sequence 39_*C*01-B*12*. The presence of two *SVA-HB* within the labelled *HERVI* sequence may be an assembly error for haplotypes 39_*C*01-B*12* and 94_*C*01-B*51* (Table S2). The SNP crossovers are indicated at the boundaries of the SNP-poor region (yellow boxes) and SNP-rich region (green boxes). (B), the SNP crossover indicated at the boundaries of the SNP-free (poor) region (yellow boxes) and SNP-rich region (green boxes) in a sequence alignment of 39_*C*01-B*2705* and 66_*C*02-B*2705* (Table 7). (C), the SNP crossover within HLA-B (labelled horizontal arrow) indicated at the boundaries of the SNP-rich region (green boxes) and SNP-free (poor) region (yellow boxes) in a sequence alignment of 92_*C*01-B*51* with 94_*C*12-B*51* and 46_*C*15-B*51* (Table 7). Sequence 94 has a 20-kb deletion (blue box) compared to sequences 92 and 46. The interspersed repeats in the upper sequence are indicated with the symbols used by Schwartz et al., (2000), whereas some of the noteworthy TEs are labelled.

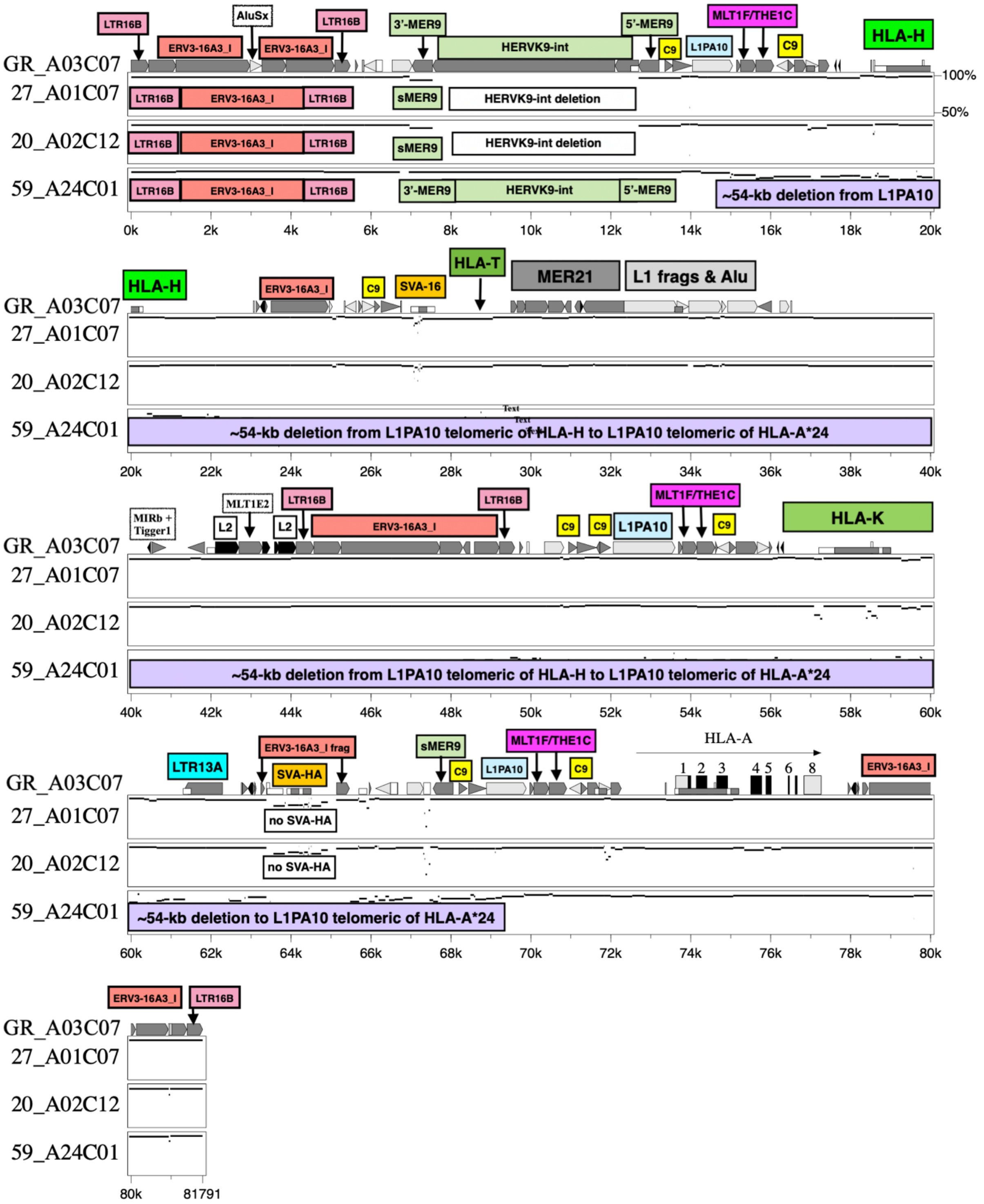

Supplementary Fig. 5. PIP output shows the location of the 54-kb deletion (purple box) between *HLA-G* and *HLA-A* in the 59_*HLA*24* haplotype compared to the aligned sequences of the *HLA-A*03* (GR), *-A*01* (27) and *-A*02* (20) haplotypes listed on the left side of the Figure. The locations of *HLA-H, HLA-T, HLA-K* pseudogenes (labelled green boxes) *HLA-A* (horizontal arrow) and some TE are indicated on the GR_*A*03-C*07* sequence. The yellow box labelled C9 represents *Charlie9*. The location of the telomeric *HLA-G* gene is not shown. All interspersed repeats in the upper sequence are indicated with the symbols used by Schwartz et al., (2000).

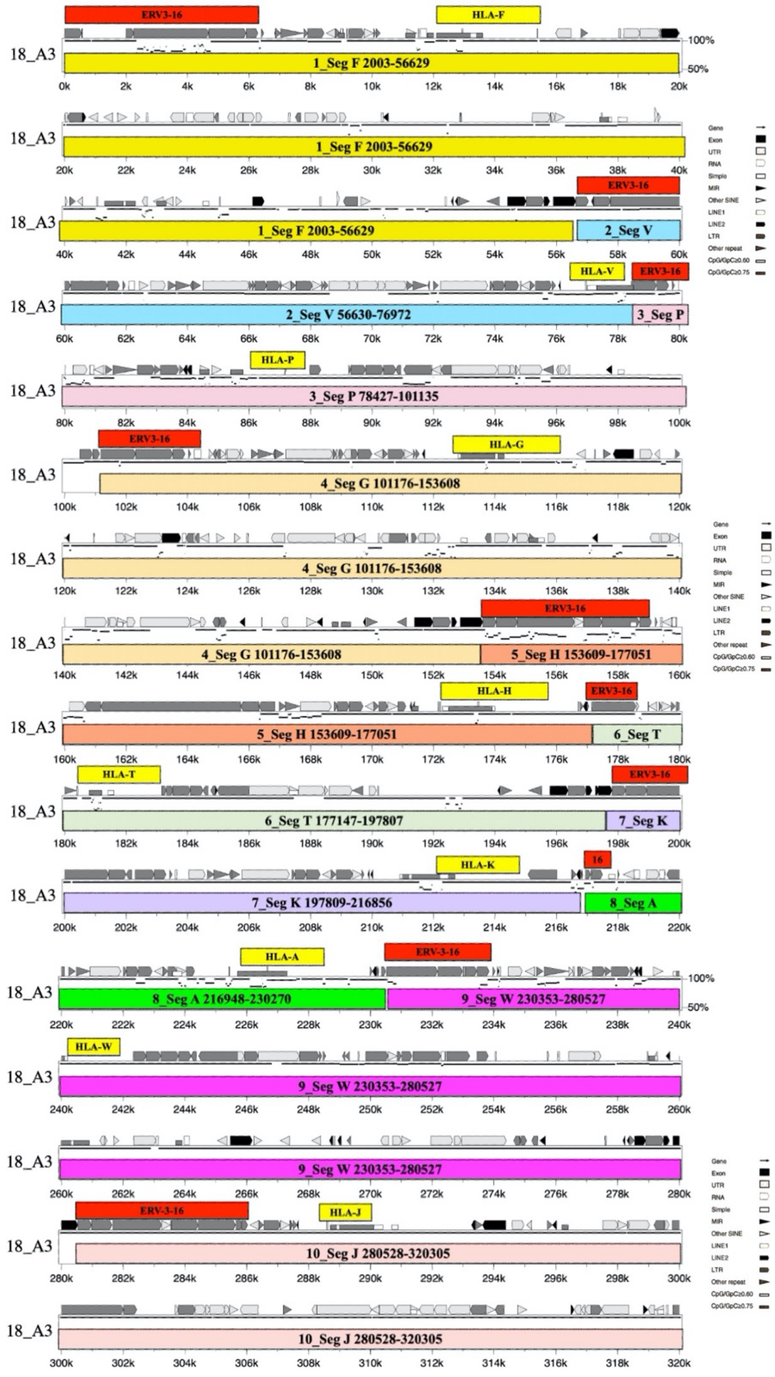

Supplementary Fig. 6. PIP output of the sequence alignment between the *4_HLA-A*03/HLA-C*07:02* haplotype (upper sequence) and the *18_HLA-A*03/A*24/C*05* haplotype (lower sequence) shows the division of the MHC class I alpha block into ten separate duplicated segments (Table 6) carrying *ERV3-16A3* (red boxes) and an HLA gene (yellow boxes) according to the evolutionary classification scheme of Kulski et al., (1999). Each imperfectly duplicated segment is labelled 1 to 10 in an extended horizontal box below the upper unlabelled sequence of the *18_HLA-A*03* haplotype from *HLA-F* in yellow segment 1_Seg F to *HLA-J* in the peach coloured segment 10_Seg J. Also, there are SNP crossovers in segment 4_Seg G at 150337 C/T and segment 8_Seg A at 229198 G/T (Table 6). All interspersed repeats in the upper sequence are indicated with the symbols used by Schwartz et al., (2000).

CLUSTAL W (1.83) multiple sequence alignment motif ATCCATG/CATGGAT in three of eight ERV3-16-A3-int elements in the alpha block.

```
1.ERV3-16/HLA-F  CCC-----------------------------------------------
2.ERV3-16/HLA-V  CAC-----------------------------------------------
3.ERV3-16/HLA-G  TCACCATGAG-------------------AGAG------------CTGAA
4.ERV3-16/HLA-H  AGGG--TGATCCATGAAAACAGGCAAAAATGGGAATTTGAAATAAGCTTC
5.ERV3-16/HLA-K  TTAG------GGGG------------------------------------
6.ERV3-16/HLA-A  AA------------------------------------------------
7.ERV3-16/HLA-W  GCA-----------------------------------------------
8.ERV3-16/HLA-J  ACC-----------------------------------------------
ERV3-16A3seq   16--------------------------------------------------65

1.ERV3-16/HLA-F  TTAAAAAAAAACACCTTTGGCTTGGGAGCACTTTCTCAGGGTATGGGTTT
2.ERV3-16/HLA-V  TAAA-----AA-TCCTTTGGCTTGGAGGCACTTTCTCAGGACATGGGTTT
3.ERV3-16/HLA-G  --------------------------------------------------
4.ERV3-16/HLA-H  TAT------AA-TCCTTTGGCTTGAAGACACTTTCTCAGGGCATGGATTT
5.ERV3-16/HLA-K  TAAA-----AA-TCCTTTGGCTTGGAGGCACTTTCTCAAGGCATGGGTTT
6.ERV3-16/HLA-A  TGT------AC----T---GCT----GCC---ATCTCG--GC------T-
7.ERV3-16/HLA-W  ------------------------------CCTT--C-------------
8.ERV3-16/HLA-J  TAAA-----AA-TCCTTTGGCTTGGAGGCACTTTCTCAAGGCATGGGTTT
ERV3-16A3seq  951--------------------------------------------------1000

1.ERV3-16/HLA-F  -----------GAAGTGA------TTCATGTGGGATCCACAACACATTTA
2.ERV3-16/HLA-V  -----------GGAGTGATTCACATTCATGTGGGATCCACAACACATTGA
3.ERV3-16/HLA-G  -----------GGAATGATTCACATTCATGTGGGATCCACAACACATTTA
4.ERV3-16/HLA-H  -----------GGAGTGATTCACATTCATGTGGGATCCTCAAAACATTGA
5.ERV3-16/HLA-K  -----------GGAGCGATTCACATCCATGTGGGATCCACAACACATTTA
6.ERV3-16/HLA-A  -----------GGGGGG--TCAGCCCCCG---------------------
7.ERV3-16/HLA-W  -----------GGAATGATTCACATTCACATGGGATTTATAATACATTTA
8.ERV3-16/HLA-J  GAAGAGGATATGGAGTGATTCACATTCATGTGGAATCAACGACACATTTA
ERV3-16A3seq              2421---------------------------------------2460

1.ERV3-16/HLA-F  AAATTGAAGTTAGCCAGCCCTCACCATCGGGCAGCCCAGGCCTGGCAGGA
2.ERV3-16/HLA-V  AAATGGAAGTTAGCCAGCCTTCACTATGGGTCAATGCAGGCCTGGTAGGA
3.ERV3-16/HLA-G  AAATGGCAGTTAGCCAGCCTTCACCATGGGTCAGCCCAGGCCTGGTAGGA
4.ERV3-16/HLA-H  AAATGGAAGTTAGCCAGCCTTCACCATGGGTCAGCCCTGGTCTGGTAGGA
5.ERV3-16/HLA-K  AAATGGAAGTTAGCCAGCCTTCACCATGGGTCAGCCCAGGCCTGGTAGGA
6.ERV3-16/HLA-A  ATGT--------G-CTGT-----------GTCCACTCAGG---------G
7.ERV3-16/HLA-W  AAATTGAAGTTAGCCAGTCTGCATCATGGATCAGCCCAGGCCTGATAGGA
8.ERV3-16/HLA-J  AAATGGCAGTTAGCCAGCTTTCACCATGGGTCAGCCCAGGCCTGGTAGGA
ERV3-16A3seq 3843--------------------------------------------------3892
```

Supplementary Figure 7. CLUSTAL W (1.83) multiple sequence alignment motif ATCCATG/CATGGAT in three of eight ERV3-16-A3-int elements in the alpha block. The T-Coffee multiple sequence alignment tool at EMBL-EBI was used to construct multiple sequences of ERV3-16A3-int in the CLUSTALW (1.83) format.
